# Slower environmental change hinders adaptation from standing genetic variation

**DOI:** 10.1371/journal.pgen.1007731

**Published:** 2018-11-01

**Authors:** Thiago S. Guzella, Snigdhadip Dey, Ivo M. Chelo, Ania Pino-Querido, Veronica F. Pereira, Stephen R. Proulx, Henrique Teotónio

**Affiliations:** 1 Institut de Biologie de l’ École Normale Supérieure (IBENS), École Normale Supérieure, CNRS, Inserm, PSL Research University, Paris, France; 2 Instituto Gulbenkian de Ciência, Oeiras, Portugal; 3 Department of Ecology, Evolution, and Marine Biology, University of California Santa Barbara, Santa Barbara, CA, United States of America; Stanford University School of Medicine, UNITED STATES

## Abstract

Evolutionary responses to environmental change depend on the time available for adaptation before environmental degradation leads to extinction. Explicit tests of this relationship are limited to microbes where adaptation usually depends on the sequential fixation of *de novo* mutations, excluding standing variation for genotype-by-environment fitness interactions that should be key for most natural species. For natural species evolving from standing genetic variation, adaptation at slower rates of environmental change may be impeded since the best genotypes at the most extreme environments can be lost during evolution due to genetic drift or founder effects. To address this hypothesis, we perform experimental evolution with self-fertilizing populations of the nematode *Caenorhabditis elegans* and develop an inference model to describe natural selection on extant genotypes under environmental change. Under a sudden environmental change, we find that selection rapidly increases the frequency of genotypes with high fitness in the most extreme environment. In contrast, under a gradual environmental change selection first favors genotypes that are worse at the most extreme environment. We demonstrate with a second set of evolution experiments that, as a consequence of slower environmental change and thus longer periods to reach the most extreme environments, genetic drift and founder effects can lead to the loss of the most beneficial genotypes. We further find that maintenance of standing genetic variation can retard the fixation of the best genotypes in the most extreme environment because of interference between them. Taken together, these results show that slower environmental change can hamper adaptation from standing genetic variation and they support theoretical models indicating that standing variation for genotype-by-environment fitness interactions critically alters the pace and outcome of adaptation under environmental change.

## Author summary

Adaptation under environmental change is expected to depend on the time available for the sequential fixation of mutations, but also on standing variation in genotype-by-environment fitness interactions. In the later circumstances, some genotypes might be initially favored but then disfavored and overtaken by other genotypes that are better at more extreme environments if they were not lost by genetic drift or founder effects in the meantime. We addressed this idea with experimental evolution in *Caenorhabditis elegans* populations with standing variation for genotype-by-environment fitness interactions and by developing a model to describe natural selection on extant genotypes during experimental evolution. We find that under slower environmental change, the genotypes that are initially selected are not the best at the most extreme environments, and as a consequence, that these best genotypes can be lost due to genetic drift and founder effects. We further find that the longer polymorphism is maintained the more likely that selective interference will reduce the best genotypes to low frequencies and increase the chances for their loss through genetic drift. We conclude that under slower environmental change adaptation will be deterred if populations can only rely on standing genetic variation.

## Introduction

With human activities contributing to climate change [1], it has become urgent to pinpoint the ecological and evolutionary mechanisms by which natural populations adapt at different rates of environmental change. It is generally accepted that lower rates of environmental change allow more time for beneficial mutations to appear, to be selected, and, as a consequence, to promote adaptation and rescue populations before environmental degradation leads to their extinction [2–6]. Experimental evolution results from studies with microbes that depend on *de novo* mutation support the idea that slower environmental change facilitates adaptation [7–10]. Unlike microbial experimental evolution, however, most species in nature have small populations, are genetically structured by geography, breeding mode or reproduction system, and might have long generation times. In all these cases, adaptation to changing environments will likely depend on standing genetic variation, and less so on *de novo* mutation [11, 12].

**Fig 1:**
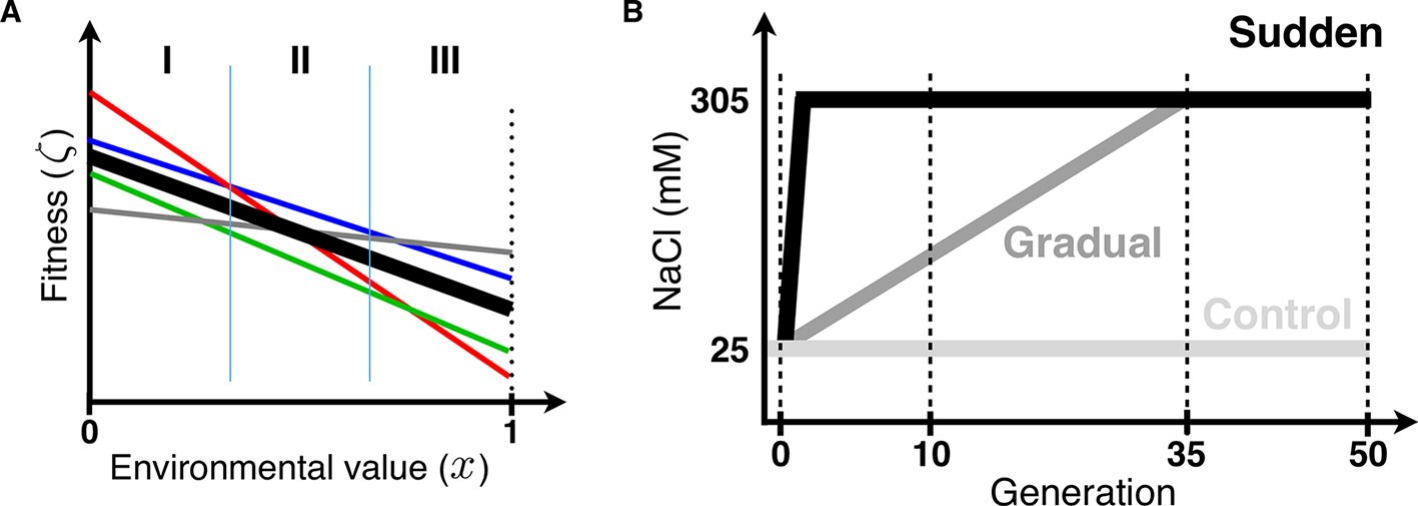
Fitness reaction norms and experimental evolution design. (A) Heritable genotype-by-environment (GxE) fitness variance implies that genotypes (colored lines) have different growth rates along the value of the environmental factor(s) considered [3, 13]. The function of each genotype’s growth rate with environmental value can be denominated its “fitness reaction norm”. Under density- and frequency-independent conditions, the relative magnitude of genotypic growth rates to the average population growth rate (thick line) will determine its deterministic frequency trajectory during evolution [25]. In general, fitness reaction norms of genotypes present in standing genetic variation will cross each other somewhere along the environmental value. A history of evolution in variable environments, with balancing selection, or population structure, for example, will determine the extent of GxE fitness variance in the ancestral population above that expected under a mutation-drift equilibrium. Crossing of fitness reaction norms means that genotypes will be favored at some environmental values while disfavored in others. Assuming no input from mutation or recombination during evolution, with a sudden change to an environmental value of 1 (vertical dotted line), from an ancestral environment 0, selection will favor the grey genotype, while under a gradual change selection will initially favor the red genotype, then the blue one, and only at a later period the grey genotype. Vertical lines broadly define the three population genetic stages expected under a gradual environmental change. In a first stage (I), the best genotype at the most extreme environment (i.e., the grey genotype) will be selected against and kept at such low frequency that its stochastic loss by genetic drift is likely under small population sizes. In a second stage (II), around where the fitness reaction norms cross, reduced fitness variance will slow down adaptive changes in allele frequency [16], and depending on the amount of standing variation [11], the best genotypes can be kept at such low frequencies that again their stochastic loss is likely. Under slower environmental change, therefore, a population will spend more time in stages I and II, reducing the chance that the best genotypes will be present at a third stage (III), when they can be selectively favored. Assuming no *de novo* mutation or recombination during gradual evolution the loss of the best genotypes will restrict adaptation to the most extreme environment [11]. At stage III, it is also possible, particularly in large populations with limited recombination between extant genotypes, that interference between the best genotypes (the grey and the blue genotypes for example) will transiently reduce selection efficacy on the best one [28, 29]. This interference process in turn will favor stochastic loss of the best genotypes if at the start of stage III they are at low frequencies. Note that without prior knowledge of fitness reaction norms, genotypes that increase in frequency and then decrease in frequency (for example the red genotype) would suggest the presence of negative-frequency dependence. (B) Experimental evolution design reported in [18] (S1 Table), used here to address whether slower environmental change hinders adaptation. A single 140-generation lab-adapted *C*. *elegans* population with abundant genetic diversity [19, 21], reproducing only by self-fertilization, was the ancestor for experimental evolution [18]. In the sudden regime, 4 replicate populations were faced from the first generation onwards to 305 mM NaCl in their growth media (high salt, black line). In the gradual regime, 7 replicate populations were faced with an 8 mM NaCl increase each generation until generation 35, being then kept at 305 mM until generation 50 (dark grey). In the control regime, 3 replicate populations were kept at 25 mM NaCl, the conditions to which the ancestor was adapted to (light grey). Here we genotype individual hermaphrodites at several time points during experimental evolution, at single nucleotide polymorphisms (SNPs) across pairs of chromosomes (vertical dashed lines, Fig 2 and Fig 3). Analysis of this data allow us to infer the fitness reaction norms of extant genotypes and predict with a deterministic model their frequency trajectories (Fig 4 and Fig 5), which are then empirically validated (Fig 6). To address the role of genetic drift and founder effects in the loss of the best genotypes during slower rates of environmental change we perform a second set of evolution experiments, where 7 of the gradual populations at generation 35 are kept in constant high salt for an extra 30 generations (Fig 7).

Adaptation to changing environments from standing genetic variation is conditional on how each extant genotype performs within the environments that may be encountered in the near future (Fig 1A). Depending on the shape of these “fitness reaction norms” [3, 10, 13], and previous evolutionary history responsible for standing genotype frequencies [5, 14], natural selection may initially favor genotypes at intermediate challenging environments that are not necessarily the best at the more extreme environments. In other words, adaptation will depend on standing genotype-by-environment (GxE) fitness variance [11], until *de novo* mutations or new recombinant genotypes escape genetic drift and start to be selected upon [15].

Understanding the population genetic dynamics of adaptation from standing genetic variation to changing environments has only been recently formalized using a moving polygenic trait optimum model [11]. In contrast to evolution from *de novo* mutation, one of the strongest conclusions from ref. [11] was that slower environmental change can restrict adaptation when evolving populations depend on standing genetic variation. One reason for this is that the maintenance of standing genetic variation for longer periods can result in reduced fitness variance and thus reduced rates of adaptation [16, 17] (Stage II in Fig 1A). Another reason is that since all populations are finite, and may suffer bottlenecks in the novel environments, the longer it takes to reach the most extreme environments (Stages I and II in Fig 1A) the more probable it is that the best genotypes are lost by genetic drift or by founder effects and thus be unavailable when populations reach the extreme environments (Stage III in Fig 1A). Because of standing variation for GxE fitness interactions, this later process will be more pronounced if the best genotypes at the extreme environments are initially selected against in the less extreme environments (Stage I in Fig 1A). In general, whether or not a population has standing genetic variation is expected to greatly affect the tempo and mode of adaptation in changing environments.

Given a fixed amount of standing genetic variation, and assuming no input of *de novo* mutation or new recombinant genotypes during evolution, we here experimentally test how the rate of environmental change affects adaptation. We investigate whether slower environmental change can constrain adaptation because of the loss of extant genotypes that would perform best in the most extreme environments. These genotypes may be lost during the period of environmental change via genetic drift or founder effects. To this end we performed experimental evolution under a sudden or gradual environmental change (Fig 1B), using populations of the nematode *Caenorhabditis elegans* with standing genetic variation and where individuals can only reproduce by self-fertilization. In this situation, we expect that asexual population genetic dynamics will be followed and that they will depend on standing GxE fitness variance. At several periods we collected genome-wide single-nucleotide polymorphism (SNP) data and used these to infer the fitness reaction norms of the genotypes that were present in the ancestral population as well as their expected frequency changes during experimental evolution due to selection. We performed a second set of evolution experiments where we test for the repeatability of adaptation in the most extreme environment to show that genetic drift and founder effects during prior gradual evolution can lead to the loss of the best genotypes and impact selection efficacy.

## Results

### Experimental evolution in changing environments

As previously reported in ref. [18], we performed experimental evolution for 50 generations in the nematode *C*. *elegans* under different rates of change in the NaCl (salt) concentration that individuals experience from early larvae to adulthood (Fig 1B). In one regime, populations were suddenly placed in high salt concentration conditions (305 mM NaCl) and then maintained in this environment for 50 generations (see Materials and Methods and S1 Text section 1.1). In another experimental evolution regime, populations faced gradually increasing salt concentrations for 35 generations, being thereafter maintained in constant high salt for an extra 15 generations. For the “sudden” regime, 4 replicate populations undergoing independent evolution were followed, while for the “gradual” regime we followed 7 replicate populations (S1 Table). All populations were derived from a single ancestor population adapted for 140 generations to lab conditions (25 mM NaCl), after initial hybridization of several wild isolates [19], that has abundant genetic diversity (expected SNP heterozygosity of ~0.3, for 1 SNP per kbp on average, presumably maintained in excess by balancing selection at overdominant loci, see [20, 21]) but where individual hermaphrodites reproduce exclusively by self-fertilization (obtained by the introgression of a male-killing mutant to the lab adapted population, cf. [18]). Hermaphrodites are expected to be mostly homozygous throughout their genome before the start of experimental evolution in changing salt environments [18, 22]. Except for salt concentrations, the same life-cycle of discrete and non-overlapping generations at stable census population sizes of 10^4^ hermaphrodites at the time of reproduction were maintained as during lab adaptation. Lab adaptation occurred under partial self-fertilization and outcrossing, with an estimated effective population size of the order of 10^3^ [20]. With exclusive self-fertilization this number should be halved to at least 500 [22]. A control regime with 3 replicate populations was also maintained at the 25 mM NaCl conditions of lab adaptation. Given exclusive self-fertilization, the expected effective population sizes, and the time span of experimental evolution, *de novo* mutation or new recombinants from standing genetic variation should not contribute much to adaptation to high salt concentrations [11, 23].

### Observed population genetic dynamics during experimental evolution

**Fig 2:**
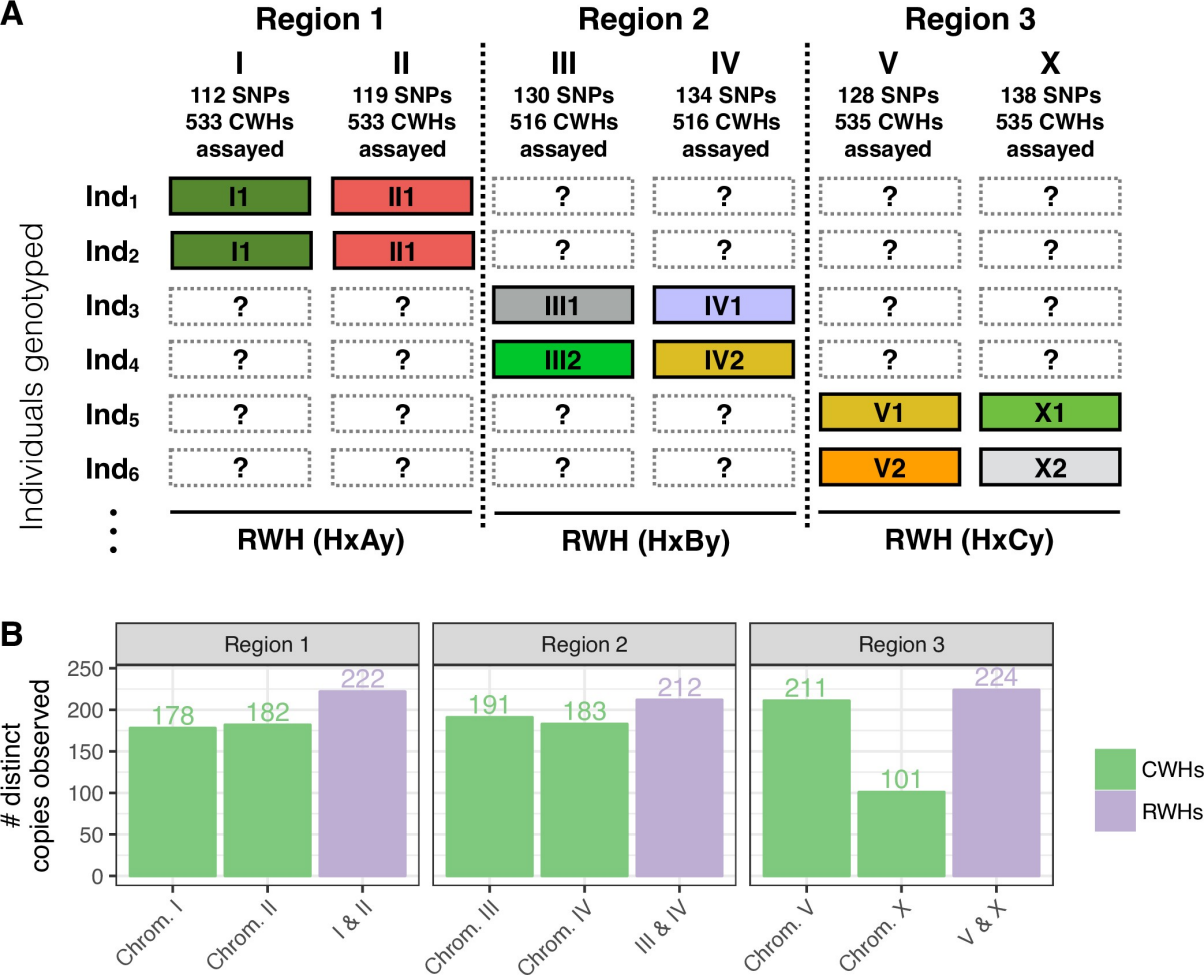
Standing genetic variation. (A) Each of the individuals sampled from the various populations during experimental evolution were genotyped for biallelic SNPs located in a pair of chromosomes. This approach was employed due to the limited amount of genomic DNA available for the genotyping method used. The pairs of chromosomes are referred to as: region 1 (chromosomes I and II), region 2 (chromosomes III and IV) and region 3 (chromosomes V and X). The haplotypes defined by the SNPs genotyped in a certain chromosome are referred to as chromosome-wide haplotypes (CWHs). In this way, the CWHs in the chromosomes that were not assayed in an individual can be conceptualized as missing data, denoted by the interrogation marks. Shown are the number of SNPs assayed in each chromosome, and the total number of CWHs that were measured, after quality control. Details on the SNPs density and number of individuals genotyped in each of the populations can be found in S1 Fig. (B) Given the genotyping data, a CWH consists of the alleles observed in each of the target sites in a single chromosome. The alleles observed in the target sites in the respective pair of chromosomes define the region-wide haplotype (RWH) for the individual, equivalent to concatenating the two corresponding CWHs. The figure shows the number of distinct CWHs and RWHs observed within each region, computed considering the full dataset (across time points and populations sampled). Since a RWH is defined by the CWHs in each of the two chromosomes in a region, the number of distinct RWHs is bounded below by the minimum of the number of distinct CWHs observed in the two respective chromosomes (reflecting the existence of linkage disequilibrium), and bounded above by the product of these two numbers (no linkage). The data indicate that linkage is strong since the number of distinct RWHs is comparable to those of distinct CWHs but not complete. Similarly, the number of lineages present in the lab-adapted ancestral population is bounded below by the product of the number of distinct RWHs in each of the three regions, indicating that the number of lineages in the ancestral population must be greater than or equal to 212. Data available from the Dryad Digital Repository: https://doi.org/10.5061/dryad.76n6f7c [54].

During experimental evolution, we measured the frequency of biallelic SNPs obtained from genotyping hermaphrodites in the ancestral population and the evolved populations at generations 10, 35 and 50 (Figs 1B and 2A). All replicate populations from the control and sudden regimes were genotyped, while from the gradual regime we genotyped 4 out of the 7 replicates (see Materials and Methods and S1 Text section 1.6 for the genotyping protocol, and S1 Fig for SNP density and sample sizes). SNPs were chosen based on the known diversity present in the 140 generation lab adapted population [21], and the expected genetic distance between them [24](S1A Fig). Given the limited amount of genomic DNA per hermaphrodite to perform whole-genome genotyping at a high density, we chose to genotype each hermaphrodite only in a pair of chromosomes (*C*. *elegans* is diploid with six similarly-sized chromosomes, for a genome of 100 Mbp), with the objective of sampling haplotypes at relatively low frequencies (Fig 2A).

With self-fertilization and complete linkage disequilibrium, the number of observed chromosome-wide haplotypes (CWH) should be similar to the number of observed “region-wide” haplotypes (RWH), each defined by a pair of homozygous chromosomes in each hermaphrodite (Fig 2A). We estimate, however, that linkage disequilibrium is not complete since when we take the data from all populations and time points into consideration between 5% and 21% more RWHs than CWHs are found, depending on the region (Fig 2B), with the majority of them being at low frequencies across the dataset (S2 Fig). We estimate that the ancestral population must have segregated at least 212 RWHs (the minimum observed number), and by extrapolation at least the same number of whole-genome haplotypes although many more are possible (see S1 Text section 1.11).

**Fig 3:**
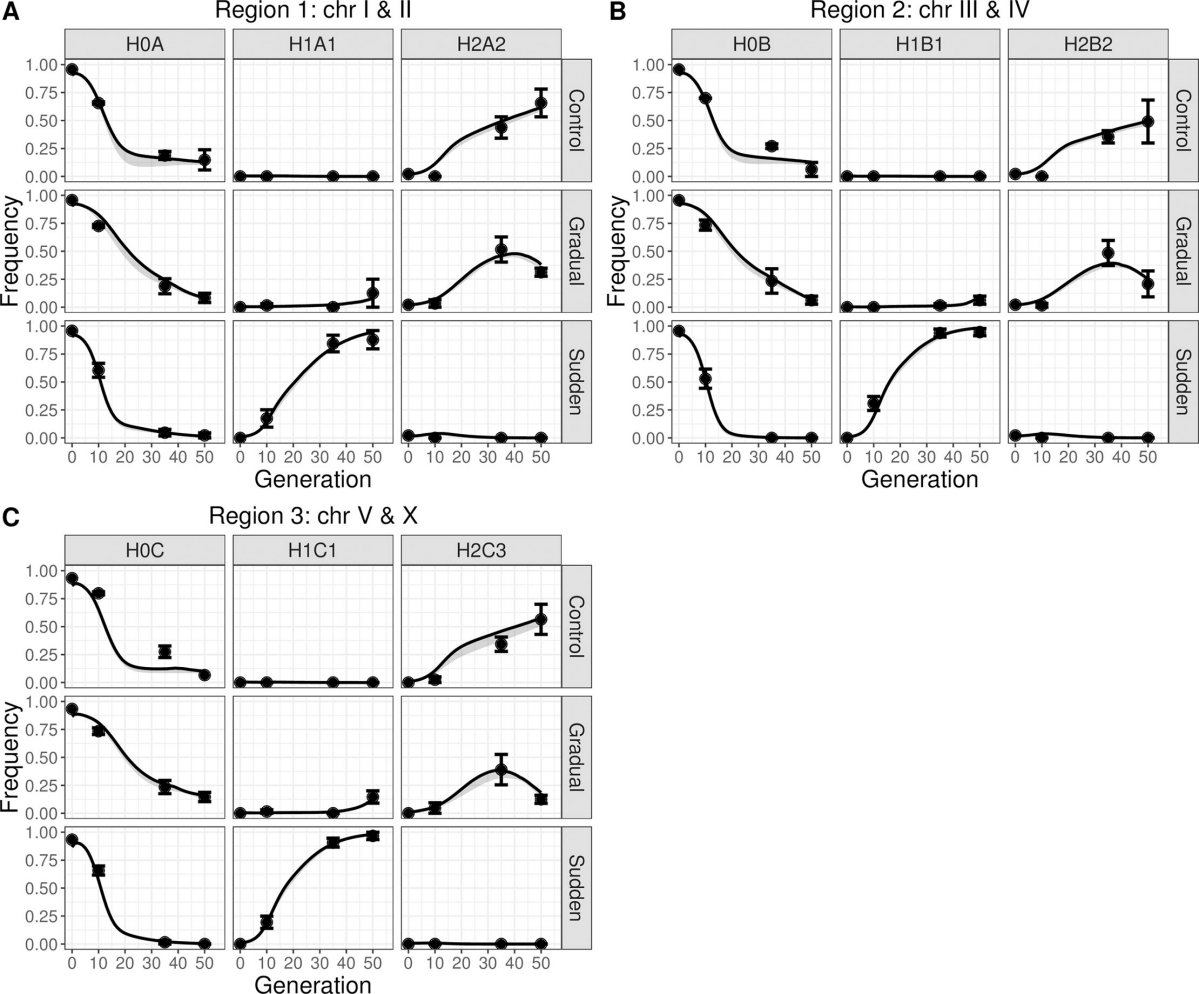
Observed experimental population genetic dynamics. (A-C) Observed frequency of region-wide haplotypes (RWH) during experimental evolution, for region 1 (A), region 2 (B) and region 3 (C). Upper rows show the responses in the control populations, middle rows for the gradual populations and bottom rows for sudden populations. Left columns show the class of minor frequency RWH that were grouped into a single class (named H0A, H0B and H0C). They indicate that the majority of haplotypes are quickly selected against under all experimental evolution regimes. Middle and right columns show the two haplotypes showing the greatest frequency change during experimental evolution. Points and error bars are the mean and one standard error of the observed haplotype frequencies among replicate populations. Line and shaded grey area are the frequency RWH trajectories inferred by modeling linear fitness reaction norms (see next Fig 4 and Fig 5). Data available from the Dryad Digital Repository: https://doi.org/10.5061/dryad.76n6f7c [54].

To facilitate computation, we grouped minor frequency region-wide haplotypes (RWHs) in each replicate population into a single class (see S1 Text section 1.10, and S2 and S3 Figs). We find that the majority of RWHs in the ancestral population are quickly selected against under all experimental evolution regimes (Fig 3). By generation 50, all populations are dominated by a single RWH in each of the two-chromosome regions. Populations faced with a sudden change in the first generation followed by constant high salt (305 mM NaCl) consistently show a single haplotype sweeping and nearing fixation by generation 50. In contrast, populations faced with a gradual increase in salt until generation 35 showed a different haplotype initially sweeping but then reverting in frequency when they were kept in the target high salt environment for another 15 generations. Control populations also show that a single RHW per genomic region sweeps through them. This is the same haplotype as that found during initial gradual evolution, suggesting continued lab adaptation under exclusive self-fertilization independently of salt [18].

### Modeling selection in changing salt environments

Since experimental evolution occurred under exclusive self-fertilization and we assume complete homozygosity, the fitness reaction norms of genome-wide haplotypes, here defined as “lineages”, are the key variables for describing selection in changing salt environments, and thus the eventual outcome of adaptation to the extreme high salt environment (Fig 1). The non-monotonic RWH frequency dynamics observed in the gradual populations in particular (Fig 3) can be explained by the crossing of fitness reaction norms somewhere along the salt gradient but, conceivably, also by negative frequency-dependent selection among segregating lineages [15].

To detect selection in changing environments, we adapted standard population genetics modeling [25] to infer the fitness reaction norms of segregating lineages and their expected frequency dynamics (S1 Text section 1.8). We model a single additive multi-allelic locus in effectively asexual populations, and thus do not specifically account for dominance or epistasis. We further modeled deterministic environmental and population genetics (i.e., there are no genotype frequency changes and no genotype extinction/fixation due to random environmental fluctuations or finite population sizes), with discrete non-overlapping generations and viability selection.

**Fig 4:**
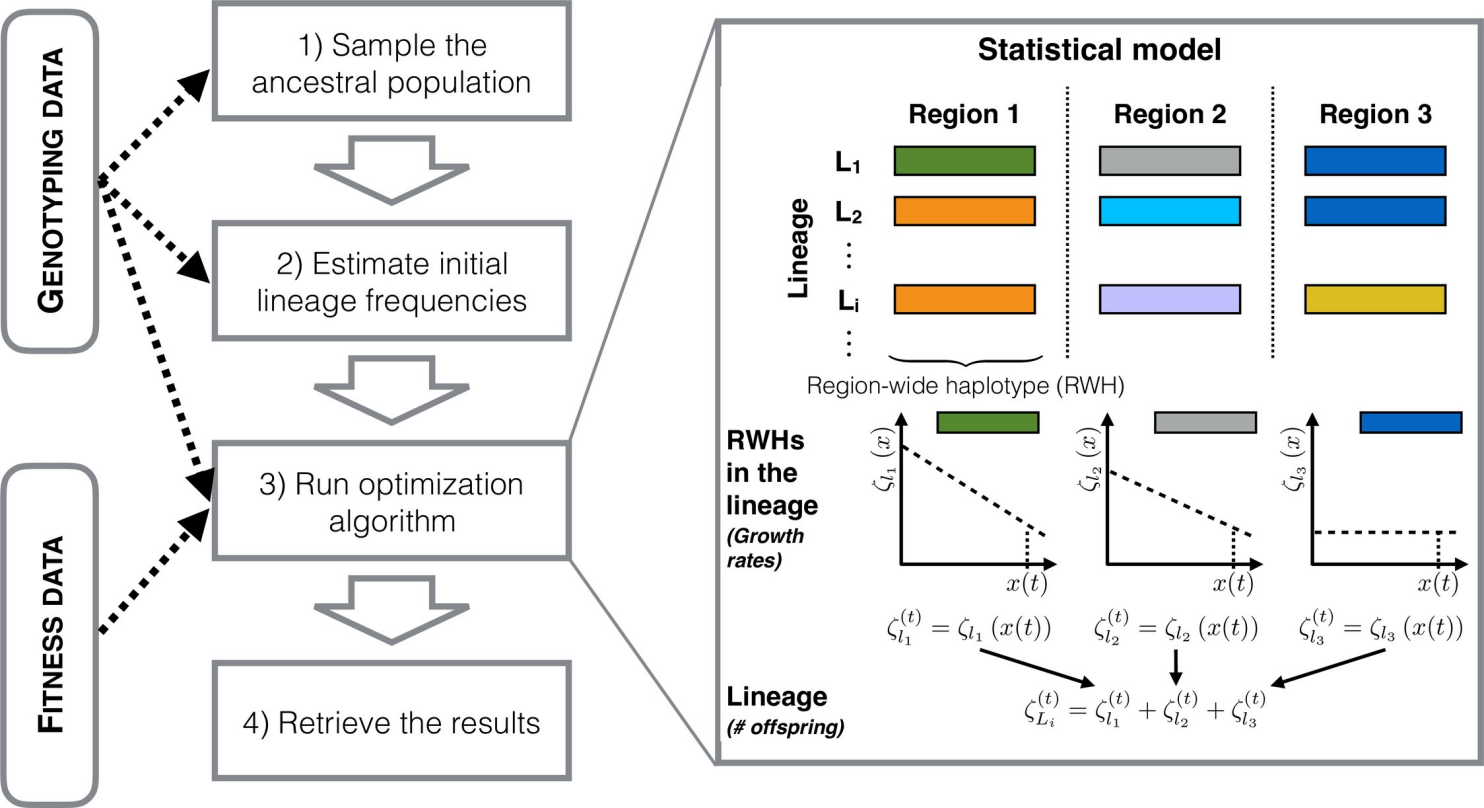
Overview of the model used for reconstructing the lineage fitness reaction norms and their frequency dynamics. In our genotyping scheme (Fig 2), the combination of region-wide haplotypes (RWHs, Fig 2 and Fig 3) define standing ancestral variation in genome-wide haplotypes or “lineages”. Fitness reaction norms are inferred from the fitness data (on the ancestral population, see Fig 6) and the genotyping data (Fig 3) assuming that the log-transformed RWH fitness reaction norms follow specific functional form, for example a linear function of the environmental value. The lineage fitness reaction norm of a lineage is the sum, in log space, of the component RWH reaction norms.

We consider that the environment faced in a given generation is represented by a single environmental value *x*, in our case corresponding to the NaCl concentration (Fig 1B). A population is composed of *G* genome-wide lineages, with the fitness reaction norm for lineage *k* being described by *λ*_*k*_(*x*) (Fig 4), corresponding to the expected absolute number of live offspring produced under environment *x*. Selection is defined by the per generation growth multiplier (growth rate) of each lineage relative to mean population fitness–with the frequency of each lineage expected to follow a deterministic logistic frequency trajectory [25, 26]. Our model allows for any parameterization of the fitness reaction norms although we only investigate linear and quadratic functions.

### Inferring the fitness reaction norms of standing genetic variation

To infer the frequency dynamics of the lineages during experimental evolution, we developed a maximum-likelihood model that estimates the parameters describing the fitness reaction norms of these lineages (S1 Text section 1.9). For this, we rely on genotyping data, consisting of the number of each RWH observed when genotyping the populations in various time-points. Since the model is parameterized on absolute fitness, we also rely on fitness data, which serves to properly scale the estimated parameters.

Inference is done in several steps, illustrated in Fig 4. We first sample the lineages that likely compose the ancestral population, taking the sample sizes and estimated RWHs frequencies into consideration, since the true lineage identities and their starting frequencies are unknown (S4 Fig and S1 Text section 1.11). We then estimate the parameters for the fitness reaction norms of the various RWHs constituting a lineage (each determined by the combination of sampled RWHs, assuming linkage equilibrium among them), and define that the lineage parameters are the sum, in log space, of their constituent RWH parameters (Fig 4). The final likelihood depends on the probability of observing the mean ancestral population fitness (in low and high salt, see below) and the observed RWH time series (for all populations and regimes), given the sampling done to identify the lineages and their frequencies in the ancestral population.

**Fig 5:**
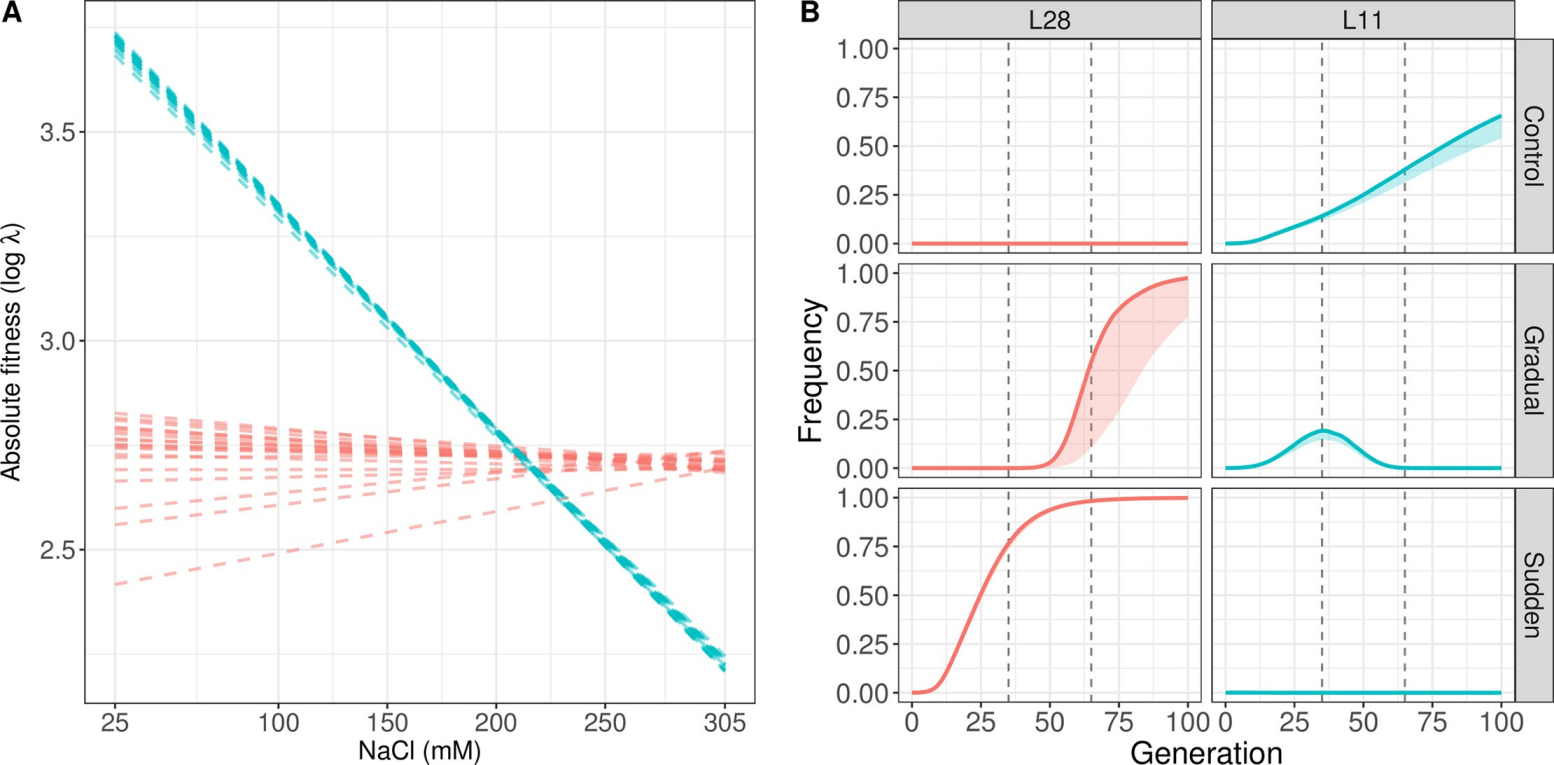
Fitness reaction norms of the two lineages explaining most population genetic dynamics. (A) From Fig 3, RWHs H1A1, H1B1 and H1C1 define lineage L28, while RWHs H2A2, H2B2 and H2C3 define lineage L11 (see also S5–S7 Figs and S2 Table). The figure shows the inferred lineage fitness reaction norms of the L28 (red) and L11 (blue) lineages, when sampling standing genetic variation of the ancestral population 20 times. (B) We modeled selection assuming infinite population sizes and that the lineage frequency dynamics were a deterministic function of the previous generation. Predicted L28 and L11 frequency trajectories under our model with the linear reaction norms of (A). Trajectories were evaluated over 100 generations, assuming that the gradual populations would be kept under constant high salt from generation 35 onwards (left vertical dashed line). The second set of evolution experiments (described below, Fig 7), were run for 30 generations from the 7 replicate gradual populations at generation 35 (right vertical dashed line). Detailed trajectories for other inferred lineages are shown in S8 Fig. Shaded colors correspond to the credible intervals obtained when sampling the ancestral population 20 times, with the line showing the median. S9 Fig shows the RWHs trajectories and S10 Fig shows lineage trajectories under quadratic fitness reaction norms. S11 and S12 Figs show the expected mean and variance in population fitness under linear and quadratic functions. Data available from the Dryad Digital Repository: https://doi.org/10.5061/dryad.76n6f7c [54].

We initially modelled linear fitness reaction norms and found that two lineages dominate the population genetic dynamics. The measured RWH frequency dynamics (Fig 3) are consistent with a single lineage sweeping through the sudden populations (Fig 5), which we label L28 (see below). In contrast to the sudden populations, the gradual populations had an initial increase of a lineage other than L28 (labeled L11), but then started to be overtaken by L28 after the 15 generations of high salt (Fig 5; see S5–S7 Figs for detailed frequency dynamics of major constituent RWHs in each genomic region in all replicate populations and regimes). L11 clearly shows a non-monotonic trajectory in the gradual populations, initially being positively selected and later being negatively selected. Under all experimental evolution regimes, a few other lineages are predicted to also explain population genetic dynamics, although these lineages do not regularly approach a frequency above 15% at any period (S8 Fig: see, for example, lineages 13 and 20 in the sudden and gradual populations, or lineage 470 in the control populations). We reach the same conclusions regarding RWHs (S9 Fig) and lineage (S10 Fig) frequency dynamics when we used a quadratic parameterization for the reaction norms.

S11 and S12 Figs show the expected dynamics of the mean and the variance in population fitness under linear and quadratic models, respectively. Under the linear model, adaptation to intermediate salt conditions in the gradual regime results in a great loss of fitness variance. At the same time, mean population fitness also decreases, a result that is consistent with the existence of an adaptive “lag load”, cf. [2, 11], since L28 is for most periods not being selected. In the sudden regime, mean population fitness strictly increases while L28 is being positively selected. Under the quadratic model, dynamics are more idiosyncratic in the gradual regime, but mean population fitness decreases to similar levels as in the linear model, and then recovers at a similar pace.

### Empirical validation of fitness reaction norms

**Fig 6:**
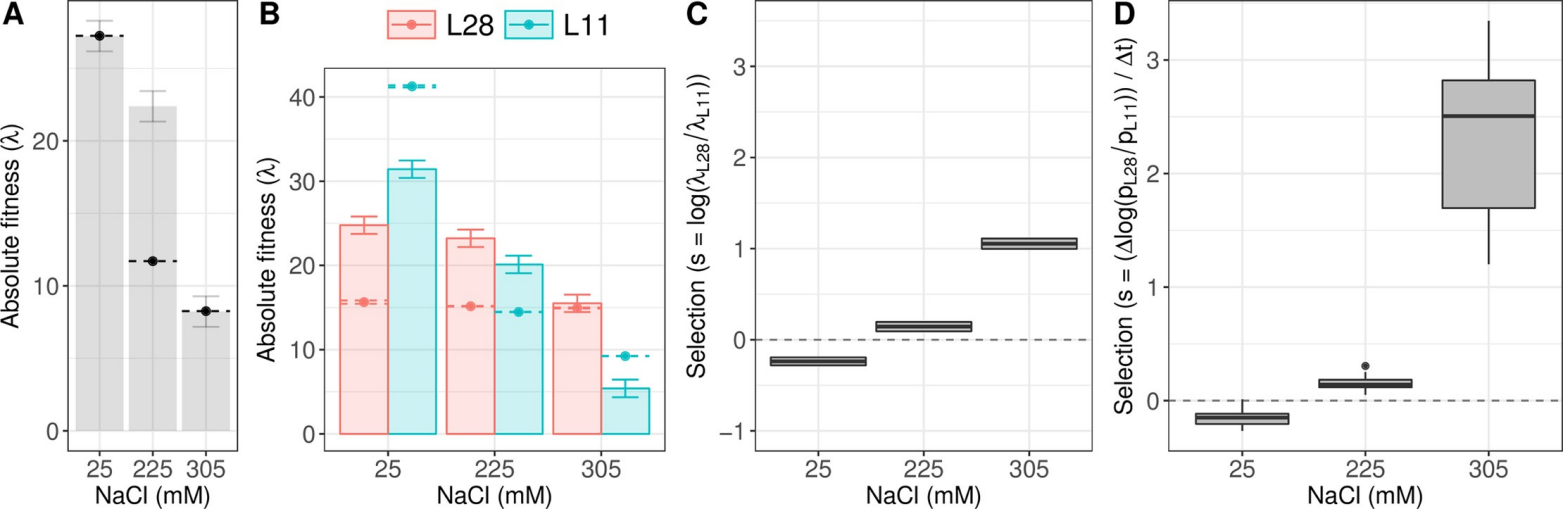
Evaluating the model predictions using fitness data. (A) Absolute fitness of the ancestor lab-adapted population at three salt concentrations (mean ± SE). The point and dashed error show the expected absolute fitness of the ancestral population, when modelling linear fitness reaction norms of all segregating lineages (from S11 Fig). At 25 mM and 305 mM NaCl the predicted values exactly match the observed values since they were used for inference. (B) Absolute fitness of the lines L28 and L11 at three salt concentrations (mean ± SE), with points and dashed lines as in (A) (from Fig 5A). (C) From (B), estimates (mean ± SE) of the expected relative fitness of L28 to L11 at three salt concentrations. (D) Similar to (C), but estimates from competitive fitness assays between L28 and L11. From ref. [21], the two inferred L28 and L11 lineages were identified after genome-wide sequencing of 100 lines derived from two gradual populations at generation 50 (S13 Fig and S2 Table). For the competitions shown in (D), frequency estimates of L28 and L11 were obtained using pooled-genotyping data on 18 SNPs that differ in L28 and L11 (see S14 Fig for calibration curves on these SNPs, and S15 Fig for our ability to differentiate L28 from L11). Data available from the Dryad Digital Repository: https://doi.org/10.5061/dryad.76n6f7c [54].

We measured the ancestral population absolute fitness as the growth rate over one generation at 25 mM and 305 mM NaCl to help with the inference of fitness reaction norms (see previous section, S1 Text section 1.3). We first sought to validate the analysis by measuring the ancestral population absolute fitness at an intermediate salt concentration (225 mM NaCl). Results show that there is a large difference between the expected and observed fitness values at 225 mM NaCl (Fig 6A), although they are intermediate to 25 mM and 305 mM NaCl. The discrepancy between observed and expected fitness values was anticipated since our inference at 225 mM NaCl was only informed by the observed RWHs frequencies in the gradual populations at generation 25 (Fig 1B and S5–S7 Figs).

More directly, we sought to validate the analysis by measuring the fitness reaction norms of the two lineages (L11 and L28) that appear to dominate the population genetic dynamics during experimental evolution (Fig 5 and S8 Fig). Using whole-genome sequencing data on 100 lineages derived from two gradual populations at generation 50 (as reported in [21]), we identified those corresponding to L28 and L11 (S13 Fig and S2 Table). Our model predicts that the linear or quadratic fitness reaction norms of these two lineages cross between 200–250 mM NaCl (Figs 5A and S10A). To test this prediction, we revived L28 and L11 from frozen stocks and assayed their absolute fitness at 25 mM, 225 mM and 305 mM NaCl. Absolute fitness was measured as the growth rate over two generations under non-competitive conditions. We find a close agreement with the model in that the lineages’ reaction norms cross at about 225 mM NaCl (Fig 6B and 6C), even if the observed values are higher than the predicted ones. At 25 mM NaCl there is a larger difference between observed and expected fitness values than at other salt concentrations. Differences between observed and expected fitness values can be explained by the low frequency of these two lineages in the ancestral and control populations (those that experienced 25 mM NaCl), and the gradual populations when at 225 mM NaCl by generation 25. Supporting our interpretation, the observed fitness values at 305 mM NaCl closely match the expected fitness values (Fig 6B and 6C), particularly for the L28 lineage. In this case the inference was mostly informed by the lineages segregating in the sudden populations, as they always experienced this salt concentration during experimental evolution (S8 Fig).

It is possible that non-transitive interactions between standing genetic variation, in particular because of negative frequency-dependence, could in part also explain the discrepancies between observed and expected absolute fitness values in the ancestral population and the L28 and L11 lineages. To test for this possibility, we conducted head-to-head competitive (relative) fitness assays between L28 and L11 (S1 Text, section 1.4). In these competition assays, performed for 2 consecutive generations, both lines were initially placed at 1:1 ratios at the usual population sizes, noting that these frequency ratios between L28 and L11 were never realized during experimental evolution (Fig 5B). The results from the competition assays are qualitatively similar to those under non-competitive conditions (Fig 6D, compare with Fig 6C). Non-transitive interactions between L28 and L11 therefore do not appear to be significant in explaining differences between observed and inferred fitness values.

Besides the uncertainty in estimating the frequency of segregating lineages, the discrepancy between observed and expected ancestral and lineage fitness can be explained by how well the parameterization of the reaction norms is done. For example, in the linear model variance in fitness as a function of salt levels must be strictly correlated while in the quadratic model the extra parameter allows the variance in fitness to differ between salt levels. Since power to infer fitness at low salt is generally weak, predictions with the quadratic model will necessary be less precise.

### Gradual environmental change hinders adaptation to high salt

So far, our experiments and modeling demonstrate that the population genetic dynamics under different rates of environmental change are contingent on the GxE fitness variance present in the ancestral population. We found that lineage L28 is the best genotype in high salt, and therefore–assuming no *de novo* mutation or recombinants–adaptation can be hindered under slow rates of environmental change if the loss of this lineage by genetic drift or founder effects is more probable than under fast rates of environmental change (see Introduction and Fig 1A). From our model, we expect the L28 frequency in the ancestral population and in the gradual populations at generation 35 to be negligible (Fig 5). The model assumes deterministic frequency dynamics and infinite population sizes and thus cannot be verified with the experimental data, since, for example, some of the replicate gradual populations could have lost L28 by the time they reached generation 35. Although some of the region-wide haplotypes constituting the L28 lineage are observed in the 4 replicate gradual populations at generation 35 (S5–S7 Figs), they not only are at low frequencies but could also be detected as part of other lineages (S8 Fig and S2 Table).

**Fig 7:**
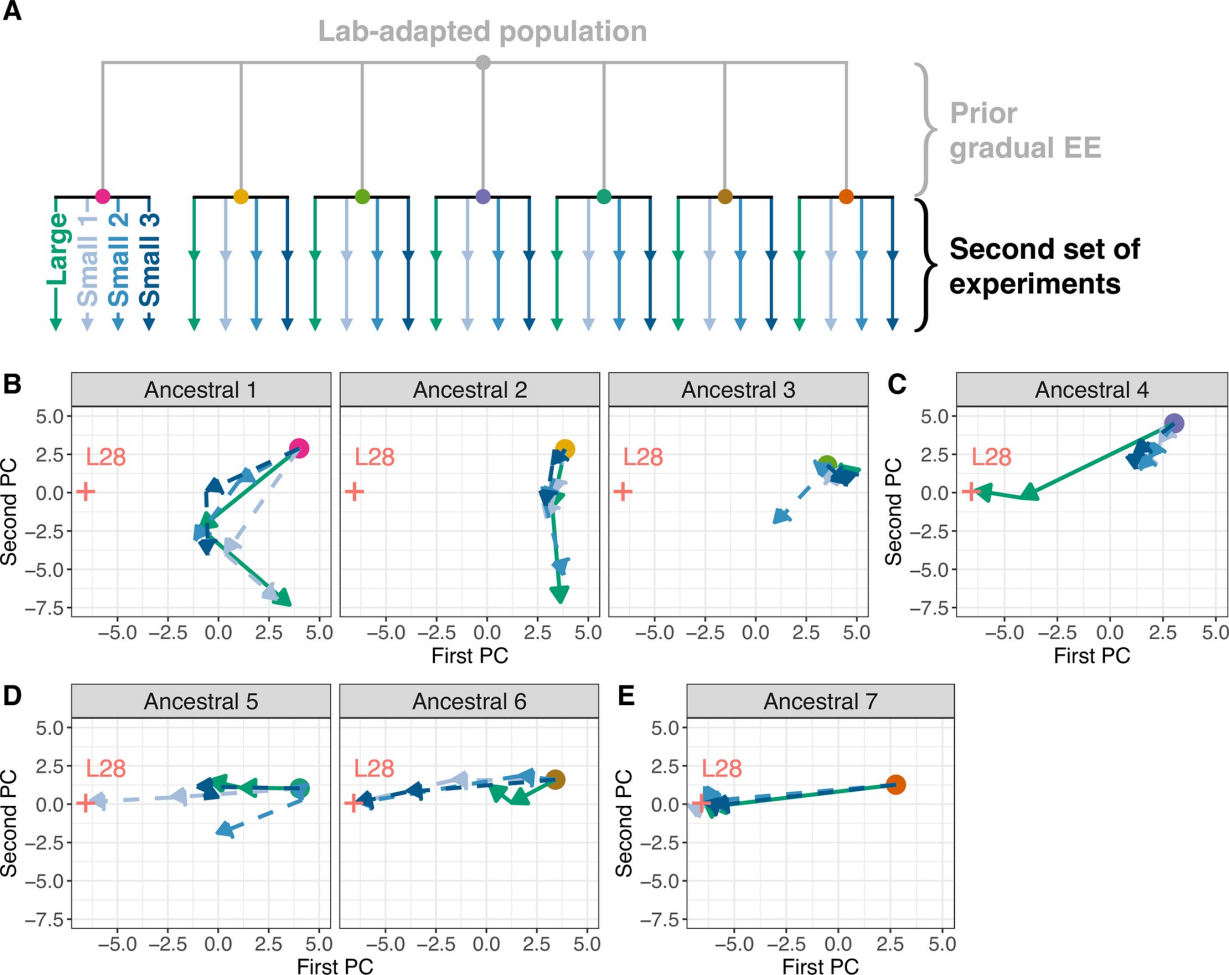
Prior gradual evolution can restrict future adaptation to high salt. (A) Experimental evolution design at different population sizes. Seven gradual populations at generation 35 become the ancestors (colored dots) for continued evolution in constant 305 mM NaCl for an extra 30 generations under large (green) and small (blue) population sizes. Populations were pool-genotyped for 18 SNPs differentiating L28 from all other segregating lineages, after 15 and 30 generations (arrows’ heads). (B-E) Trajectories for the replicate populations under large and small population sizes, from the seven ancestor populations. These trajectories are based on principal component (PC) analysis of allele frequency data, with the two first axis accounting for more than 70% of the variance (see S16 and S17 Figs). Red crosses indicate the likely position of the L28 lineage in this PC space. Analysis of the probability of a sweep by L28 in each population is shown in S17 Fig. Data available from the Dryad Digital Repository: https://doi.org/10.5061/dryad.76n6f7c [54].

To address if genetic drift and founder effects can be implicated in the loss L28 under slower rates of environmental change, we revived frozen stocks from the 7 replicate gradual populations at generation 35 (Figs 1B and 7A), and performed a new set of evolution experiments at two different population size regimes, 10^4^ and 2·10^3^, for 30 generations in constant high salt (Fig 7A, see Materials and Methods and S1 Text section 1.5). In this second set of experiments, we refer to each of the 7 gradual populations as ancestrals #1–7 (S1 Table).

Two main factors, prior genetic drift or selection, could lead to differences in the adaptive responses observed from each of the new ancestral populations, as well as between population size regimes. First, the best high salt lineage determined from the first set of evolution experiments, L28, may have been lost by genetic drift before the second set of experiments started. The freezing and reviving process of the populations could also have resulted in L28 loss; in this case a population size bottleneck would cause a founder effect for the second set of experiments. The second factor is that the efficacy of selection on the best lineages should be lower because of stronger genetic drift in small populations [22].

At two time points during this second set of evolution experiments, a small number of SNPs across the genome were genotyped in pools of individuals, chosen to maximize the ability to distinguish lineage L28 in large samples (S14 and S15 Figs). We then calculated the probability of a L28 sweep under the inferred fitness reaction norms of segregating lineages found above, given the pooled genotyping data (S1 Textsection 1.12, S16 and S17 Figs). Under our genotyping protocol and analysis, an L28 sweep does not imply its fixation during the time frame of experimental evolution–indeed, with deterministic dynamics we predict that after 30 generations at high salt L28 frequency would only reach 50% (Fig 5)–nor are we able to determine if lineages other than L28 sweep through the populations.

We found that the evolutionary responses from the 7 ancestral populations fell into four distinct categories (Fig 7). The first category demonstrates the consequences of a founder effect on adaptation to high salt since the L28 lineage did not sweep through any population (Fig 7B and S16 and S17 Figs). Yet, from at least the first two ancestrals, another unidentified lineage appears to have responded more rapidly at large population sizes than at small population sizes, a result indicating higher selection efficacy at larger population sizes. From the third ancestral, we can only conclude that the L28 lineage was lost before starting the second set of experiments.

A second category of responses more directly illustrates the effects of genetic drift on adaptation (Fig 7C). From the fourth ancestral, the L28 lineage swept rapidly in the high population size regime, while at smaller population sizes the response was more restricted and L28 probably lost during evolution in high salt.

The third category also illustrates the effects of genetic drift to adaptation, although in the opposite sense (Fig 7D). From the fifth and sixth ancestrals, we find that the L28 lineage swept in a fraction of the populations, but exclusively in those with small population sizes. This seemingly puzzling result can be explained if one postulates that, together with L28, the ancestor populations segregated at a relatively high frequency other unidentified lineages that were almost as fit as L28 in high salt (such as lineage 13, see S8 Fig). Interference at large population sizes could have transiently kept L28 at a lower frequency than that expected [27], possibly promoting its extinction by genetic drift [28, 29]. In contrast, at small population sizes the loss of high fit lineages (or maintenance at very low frequencies) by genetic drift might have in turn freed selection to sometimes favor L28 unconstrained.

Despite population size regime, all populations derived from the seventh ancestral showed rapid sweeping of L28 (Fig 7E). This indicates that L28 was initially at a relatively high frequency in the seventh ancestor, when compared to the fourth ancestor (where L28 rapidly sweep only in the large population). At generation 35 of gradual experimental evolution, we observed that one of the constituent region-wide haplotypes of L28 was present in the seventh ancestor, while absent in the fourth ancestor, in line with the expected initial frequency differences in L28 between them (S5–S7 Figs). However, without further genome-wide SNP sampling at high densities and sizes, we cannot assess how prior gradual evolution impacted L28 frequency for continued experimental evolution in constant high salt (S16 and S17 Figs).

## Discussion

### Summary of findings

Adaptation to extreme environments under different rates of environmental change is expected to depend on ancestral GxE fitness variance and thus on the shape of fitness reaction norms and relative frequencies of extant genotypes. Ignoring the input from *de novo* mutations and of new recombinant genotypes during adaptation, our experiments in a gradually changing environment show that genotypes initially favored by selection are later selected against when they are overtaken by better genotypes as the environment becomes more extreme. Further, because adaptation to intermediate environments during gradual evolution decreases the frequencies of the genotypes that are most adapted to the extreme environments, these best genotypes can be lost before populations reach such extreme environments.

### Detecting selection during experimental evolution

During the last decade there has been a substantial effort in the development of inference methods to detect selection on DNA sequence diversity during experimental evolution [30–32], although no prior work has explicitly dealt with changing environments. Without directly assaying fitness of each individual genotype, our approach allowed us to infer the distribution of standing GxE fitness variance, inference of both genotype frequencies and the genotypic effects across an environmental (salt) gradient. Based on the inferred distribution we could have predicted the outcome of selection under any rate of environmental change, although we only explored the experimental evolution regimes that were actually performed. Future studies could therefore investigate how different distributions of ancestral GxE fitness variance–in the amount of diversity and shape of reaction norms–determine the loss of genetic variance during environmental change and, for example, the mean population fitness lag load [2, 11]. Our preliminary results indicate that independently of the specific parameterization of fitness reaction norms, slower environmental change transiently results in maladaptation and ultimately delays adaptation. Reaction norms with more flexible parameterizations, however, seem to generate complex fitness variance dynamics, presumably because genotypes favored at early periods can become neutral at other periods and then again positively selected at later periods. For example, the loss of fitness variance at intermediate salt levels is more pronounced under linear than quadratic functions, although by generation 50 the mean population fitness is actually higher under the linear than the quadratic model. Despite our approach allowing for arbitrary parameterizations of the reaction norms, one can of course argue that the decision to model particular reaction norm shapes should first hinge on an understanding of individual development and physiology in the relevant environments.

The most obvious limitation of our inference method is that population size was not included as a parameter and thus we could not account for the effects of genetic drift. Such extension of the model would allow explicit predictions about the loss of genetic variance with variable population sizes and thus the probability of extinction to deteriorating environments, an especially important problem in the context of changing environments, e.g., [33]. An approach by Nené and colleagues [34], focused on the case of evolution of new haplotypes in a population via mutation and positive selection in a constant environment, could perhaps be adapted to detect selection in changing environments with stochasticity. They developed a phenomenological "delay-deterministic” model where an "effective" mutation rate was conditioned on the current frequency of the focal haplotype, with a given threshold mutation rate being parameterized to mimic the effects of genetic drift. Under a limited set of simulated data, the addition of the delay term to their deterministic model better reproduced the frequency dynamics and produced better estimates of selection coefficients. We anticipate, however, that methods explicitly accounting for stochasticity, for example Bayesian models estimated using MCMC techniques, will be necessary in order to manage computational constraints and allow for hypothesis testing and model fit evaluation.

Another future extension of our approach should be to apply it to outcrossing haploids and diploids. The model could be adapted to account for mating and recombination by finding genomic regions at high to complete linkage disequilibrium during the relative short periods of experimental evolution and treating them as we did here the “region-wide haplotypes” (RWH). But expanding our model with recombination represents a considerable challenge since it requires characterizing the degree of polygenicity for fitness [21] and whether or not accounting for dominance and epistasis is necessary [35]. With selection on new genotypes generated by recombination, as with *de novo* mutation, adaptive rates may increase if it takes longer for a population to reach extreme environments. The net effect of loss of genotypes during adaptation to intermediate environments and the production of new genotypes by recombination is not immediately clear [11], and thus reconciling experimental evolution results that depend on standing genetic variation with and without recombination with those where adaptation occurs from *de novo* mutation is a major future task.

### Adaptation from *de novo* mutation and from standing genetic variation

Experimental evolution studies with microbes that depend on *de novo* mutation suggest that adaptive gains become smaller with each mutational event, and therefore that adaptation involves diminishing-returns epistasis for fitness [36, 37]. Microbial experiments further indicate that slower environmental change allows more time for the exploration of mutational “space” and the possibility to fix mutations at intermediate environments that predispose subsequent fixation of additional mutations at more extreme environments. Such outcomes should depend on the empirical fitness relationship between alleles related by single mutational steps [38, 39]. In the study of Gorter and colleagues [10], under some stressors, slow environmental change retarded adaptation but not the fitness gains in the most extreme environments. In the study of Lindsey and colleagues [40] the populations that survived a sudden environmental change had higher fitness than those that survived a more gradual change, suggesting, just as in our experiments from standing genetic diversity, a key role of GxE fitness interactions. In changing environments, GxE fitness interactions appear to be sufficient to explain adaptation to extreme environments when evolution occurs from standing genetic variation (without recombination), while both GxE interactions and epistasis are important when evolution occurs from *de novo* mutation. Clearly, we were unable to determine if epistasis played a role in adaptation since, by definition, there was only selection between non-recombining genotypes.

Little theoretical work has focused on understanding the population genetics of adaptation from standing genetic variation in changing environments. An exception is the study by Matuszewski and colleagues [11], which explored the distribution of fitness effects of fixed alleles starting from standing variation, and with mutational input, under a moving trait under stabilizing selection and epistasis for fitness. The trait was modeled as polygenic with additive interactions between alleles (effectively a biallelic infinite-site and continuum of alleles model), with recombination rates following a Poisson distribution and cross-overs a uniform position along the genome. Matuszewski and colleagues found that populations facing a fast environmental change show larger trait changes than those facing a slow environmental change, due to increases in both the expected number of fixations and the expected trait effect per allele substitution. Although they did not analyze situations of an abrupt environmental change under complete linkage (no recombination), as in our sudden evolution experiments, they nonetheless predicted a higher number of fixations under faster environmental change, and that adaptation would be deterred under slower environmental changes. Matuszewski and colleagues further found that while fast environmental change eliminates sets of *de novo* mutations, it also helps to keep standing genetic variation until it can be picked up by selection. On the other hand, under slow environmental change, most large effect alleles are already eliminated by genetic drift (or stabilizing selection) before they could contribute to adaptation.

Although the mathematical assumptions of the model of Matuszewski and colleagues do not closely match our experimental conditions, some of their predictions are consistent with the results obtained. We found that slower environmental change allows populations to maintain more genotypes for longer than faster environmental change, and that this can compromise adaptation. Besides loss by genetic drift, one reason for compromised adaptation is that when fitness reaction norms cross, the fitness variance is reduced and adaptive rates diminished [16]. Previous demography, form of selection and degree of environmental variability will determine standing levels of genetic variation and thus from where along the environmental gradient adaptation will ensue [5] (Fig 1). If the population has already exhausted standing GxE variance, then the rate of environmental change will not affect the loss of relevant genotypes simply because they are not present in the population.

Selective “interference” is yet another process that could in part determine hindered adaptation under slower environmental change, and that could also explain why the best genotype was not favored at high population sizes in some of the high salt continued evolution experiments. In this scenario, since slower environmental change can promote the maintenance of polymorphism for longer periods, it is possible that reduced selection efficacy on the best genotypes kept them at low frequencies and caused in turn their loss by genetic drift before populations reached the most extreme environment. With recombination, interference between the best genotypes should be diminished [28, 29, 41], and hence adaptation to the extreme environments will probably not be constrained as when there is limited recombination. Selective interference has been theoretically and empirically studied for microbial evolution experiments in constant environments where the mutational supply is high enough for competing asexual lineages to interfere with each other and retard fixation of the best mutations [42, 43]. Other findings posit an important role for interference and stochasticity in maintaining the long-term standing genetic variation in sexual organisms [29, 41], in particular those reproducing by self-fertilization and with greatly reduced effective recombination rates [44, 45]. However, the importance of interference between beneficial genotypes and stochasticity in promoting their loss in changing environments remains to be explored.

### Conclusion

Understanding the outcome of selection in changing environments is complicated because the historical sequence of population genetic changes, recombination and mutational input will determine the way populations respond later in evolution. Since our experimental design used fixed standing genetic variation, with little opportunity for new mutations or recombinants, we were able to examine in isolation the interaction between the sequence of environmental change and the ancestral variation in fitness reaction norms. We demonstrated that under gradual environmental change the genotypes most adapted to the extreme environments do not rise to high frequency during the early periods at less extreme environments. This then opens the door for stochastic loss of genotypes by genetic drift and founder effects, as revealed by our continued evolution experiments. Ultimately, the combination of these processes results in greater adaptation under faster environmental change.

## Materials and methods

### Experimental evolution in changing salt environments

All populations employed are ultimately derived from a hybrid population of 16 wild isolates [19], followed by 140 generations of laboratory domestication to a 4-day non-overlapping life-cycle under partial self-fertilization (self-fertilization) at census sizes of N = 10^4^ at the time of reproduction [15, 19], and introgression and homozygosity of the *xol-1(tm3055)* sex determination mutant allele at high populations sizes for 16 generations to generate an ancestral population capable of reproduction only by self-fertilization [18]. Experimental evolution in changing environments has been previously reported (Fig 1B, [18]). Large samples of the ancestral population were revived from frozen samples [46], expanded in numbers and first larval staged (L1s) individuals seeded at the appropriate densities to three regimes. The “sudden” regime was characterized by the same conditions to which previous lab-adaptation occurred, except that the NGM-lite media (US Biological) where worms grew was supplemented with NaCl (305 mM) from the start and for 50 generations (4 replicate populations; S1 Table). For the “gradual” regime plates were supplemented with increasing concentrations of NaCl from 33 mM at generation 1 to 305 mM NaCl at generation 35 and onwards until generation 50 (7 replicate populations). A “control” regime was maintained in the ancestral environmental conditions without any salt supplement (3 replicates). Individual hermaphrodites from the ancestor population and generation 10, 35 and 50 from the sudden, gradual and control populations were handpicked for genotyping.

### Experimental evolution in constant high salt at different population sizes

All 7 replicate populations from the gradual regime at generation 35 were revived from frozen stocks, expanded in numbers for two generations, and then split into two regimes: large population sizes of N = 10^4^ and small population sizes of N = 2·10^3^ at the time of reproduction (Fig 7A). From each of the 7 gradual populations at generation 35, one replicate was maintained at large population sizes and three replicates were maintained at small population sizes. All populations were kept at constant 305 mM NaCl for 30 generations. Over 10^3^ L1s were collected per population at generation 15 and 30, for pool-genotyping.

### Ancestral population fitness assays

The ancestral population was thawed from frozen stocks and individuals reared for two generations at 25 mM NaCl before they were exposed to the three salt treatments: 25 mM, 225 mM or 305 mM NaCl (Fig 6). Following the usual culture protocol during experimental evolution, on the third generation, five Petri dishes per NaCl treatment were seeded each with 10^3^ L1s. These five plates constituted one technical replicate, and there were four for each salt treatment. After 66 h, individuals were harvested and exposed to a 1 M KOH:5% NaOCl solution (to which only embryos survive). After 16 h, debris was removed and the total number of live L1s estimated by repeated sampling of small volumes.

Statistical analysis was done based on the log-transformed per-capita L1-to-L1 growth rate values, using a linear model with the assay environment as a categorical variable. For this, the assay environment for the *i*-th measurement is denoted as *E*_*i*_, and given by: *E*_*i*_ = 0, for 25 mM NaCl; *E*_*i*_ = 1, for 225 mM NaCl and *E*_*i*_ = 2, for 305 mM NaCl. In this way, the 25 mM NaCl is taken as the reference environment. The model then takes the form:

$$\xi_{i}=\beta (E_{i})=\beta_{0}+\beta_{1}I(E_{i},1)+\beta_{2}I(E_{i},2)$$

where $I(E_{i},j)$ is the indicator function:

$$I(E_{i},j)=\left\{ \begin{array}{c} 1,\;if\;E_{i}=j\\ 0,\;otherwise \end{array}\right.$$

and *β*_0_, *β*_1_ and *β*_2_ are coefficients to be estimated.

The data was analyzed in R [47], using the following formula to specify the model in the *lm* function:

log(growthRate) ~ saltTreatment

Least-square estimates of the expected log-growth rates in each of the three assay environments were then obtained using the R package *lsmeans* [48]. Note that for inferring fitness reaction norms only the ancestral fitness estimates at 25 mM and 305 mM were used (see below).

### Identification of L28 and L11 lineages

During experimental evolution in changing environments, one lineage (whole-genome haploid haplotype) swept through the sudden populations, while another lineage was initially sweeping though the gradual populations when they were at intermediate salt concentrations (Fig 5). From two gradual populations at generation 50, we derived in [21], by repeated single hermaphrodite self-fertilization for >10 generations, 100 “lines” which were the whole-genome sequenced. Comparing the >300k SNPs in the lines with the 761 SNPs collected during experimental evolution (see below), we identified lines L28 and L11 as representatives of the lineages predicted to explain the experimental population dynamics (S11 Fig and S2 Table).

### L28 and L11 fitness assays under non-competitive conditions

We also conducted absolute fitness assays for L28 and L11 (Fig 6), in a similar manner and replication as for the ancestral population, except that L1-to-L1 growth rate data were collected for two generations. Statistical analysis was done based on the log-transformed per-capita L1-to-L1 growth rate values, with the value obtained for the *i*-th measurement denoted as *ξ*_*i*_. Since the data were gathered over two generations, we accounted for the potential presence of transgenerational effects by using a mixed effects model [49], with environment, lineage and a transgenerational component as fixed effects, and assay block (defined by when the lineages were revived from frozen stocks) as a random effect:

$$\xi_{i}=\beta (E_{i},L_{i})+\alpha (L_{i})g_{i}+\gamma (B_{i})$$

where *E*_*i*_ denotes the assay environment (*E*_*i*_ = 0, for 25 mM NaCl; *E*_*i*_ = 1, for 225 mM NaCl and *E*_*i*_ = 2, for 305 mM NaCl), *L*_*i*_ denotes the line (L11 or L28; *L*_*i*_ = 0, for L28; *L*_*i*_ = 1, for L11), *g*_*i*_ corresponds to the transgenerational component (described below) and *B*_*i*_ is the assay block (*B*_*i*_ ∈ {1,2,3}). *E*_*i*_, *L*_*i*_ and *B*_*i*_ are categorical variables, while *g*_*i*_ is a continuous variable. In this model, the transgenerational value *g*_*i*_ is given by:

$$g_{i}=\left(\frac{c_{i}-25}{305-25}\right)(t_{i}-1)$$

where *c*_*i*_ is the NaCl concentration, in mM, and *t*_*i*_ ∈ {1,2} is the generation assayed. The various terms of the model correspond to: i) *β*(*E*_*i*_,*L*_*i*_), the statistical interaction between environment and line; ii) *α*(*L*_*i*_), the line-dependent transgenerational effect; and iii) the intercept-based effect of block.

The data was analyzed in R [47], using the following formula to specify the model in function *lmer* from package *lme4* [49]:

log(growthRate) ~ saltTreatment * line + line * tGenComp + (1 | block)

With the R package *lsmeans* [48] being then used to obtain estimates of interest:

- expected log-growth rates L28 and L11 in each of the three assay environments:

~ saltTreatment * line

- expected selection coefficient of L28 relative to L11:

pairwise ~ line | saltTreatment

In both cases, the estimates obtained do not include contributions of transgenerational effects (by evaluating the model at *g*_*i*_ = 0, via parameter tGenComp = 0).

### L28 and L11 fitness assays under competitive conditions

L28 and L11 were further assayed in head-to-head competitions (Fig 6D). Lineages were revived and reared for two generations at 25 mM NaCl before they were set up at three NaCl concentrations: 25 mM, 225 mM and 305 mM. On the third generation, L1 larvae from the two lineages were mixed in 1:1 ratio, at a density of 10^3^ L1s in each of two Petri dishes per replicate assay. Each replicate assay was maintained for two generations. At both the assay generations, L1 samples were collected for pool-genotyping of single nucleotide polymorphisms (SNPs). Assays were performed in three blocks, with 3 replicates per salt concentration in each of two blocks, and 4 replicates in the third block. The data for analysis was based on the L28 and L11 SNP frequency values obtained after doing calibration curves where the ratio of both lines was known (S12 Fig). For analysis, the estimated frequencies for L28 were forced to be in the interval (0.005, 0.995).

To estimate relative fitness we calculated the selection coefficients of L28 with respect to L11, for the three assay environments considered, using a mixed effects model per SNP [49]. Each model included salt treatment and generation as fixed effects, and replicates as a random effect:

$$y_{i}=\beta_{0}+\alpha (E_{i})\;t_{i}+\gamma (R_{i})$$

where *y*_*i*_ is the logarithm of odds-ratio of the L28 allele:

$$y_{i}=\log\left(p_{i}/\left(1-p_{i}\right)\right)$$

where *E*_*i*_ denotes the assay environment (*E*_*i*_ = 0, for 25 mM NaCl; *E*_*i*_ = 1, for 225 mM NaCl and *E*_*i*_ = 2, for 305 mM NaCl), *t*_*i*_ denotes the generation, and *R*_*i*_ is the replicate (*R*_*i*_ ∈ {1,2,⋯,30}).

The data was analyzed in R [47], using the following formula to specify the model in function *lmer* from package *lme4* [49]

log(OdssRatioL28Allele) ~ generation : saltTreatment + (1 | replPop)

The selection coefficients in each of the three assay environments were obtained via the point estimates for the corresponding parameters of the model.

### Genotyping during experimental evolution in changing salt environments

Individual L4 genomic DNA was prepared with the ZyGEM prepGEM Insect kit following [20]. A total of 925 biallelic SNPs across the genome were assayed by iPlex Sequenom MALDI-TOF methods [50]. We chose the SNPs known to segregate in the lab adapted population, following [21]. Due to the limited amount of genomic DNA, each individual was assayed for two of the six *C*. *elegans* chromosomes, each pair of chromosomes being referred to as a region (chromosomes I and II: region 1; III and IV: region 2; V and VI: region 3). 64 L4s from the ancestral population and 16 L4s from each of the evolved populations at generations 10, 35 and 50 were sampled per region (3 replicate control populations, 4 replicate sudden, 4 replicate gradual).

Quality control was based on discarding SNPs with a high frequency of heterozygous calls, SNPs with a high frequency of genotyping failures (> 30%), and individuals in which many SNPs failed genotyping (> 25%). The 761 SNPs that passed quality control were imputed into chromosome-wide haplotypes using *fastPHASE* [51].

### Genotyping during experimental evolution in high salt at two population sizes

Genomic DNA from pooled samples was prepared using the Qiagen Blood and Tissue kit, and genotyped for 84 SNPs in chromosomes I, IV and V, using the iPlex Sequenom methods in 3 technical replicates for each SNP assay. In parallel, pooled gDNA was prepared to calibrate SNP L28 allele frequencies when mixed with L11 or the ancestor population at several known proportions (8–14 technical replicates each). After quality control, we retained 29 SNPs, 18 of which differentiating L28 and L11. We interpolated expected L28 frequencies from the calibration curves (S14 Fig), using Levenberg-Marquardt algorithm in R package *minpack*.*lm* [52]. For the principal component analysis of the matrix containing the frequency of the alternative alleles in each sample (Fig 7), the function prcomp in R was used.

### Modeling selection in changing salt environments

We model an asexual population of a haploid organism, and consider deterministic environmental and population dynamics, discrete non-overlapping generations and viability selection, with the only environmentally-relevant variable being the NaCl concentration. We assume an infinite population size, such that any given lineage (genome-wide haploid haplotype) never goes extinct, and that there are no density- or frequency-dependencies, and that transgenerational effects are absent.

Following for example ref. [25], a population is composed of *G* lineages, such that the frequency of the *k*-th lineage in generation *t* + 1, denoted by $g_{k}^{\left(t+1\right)}$, is given by:

$$g_{k}^{\left(t+1\right)}\propto \lambda_{k}(x(t+1))\;g_{k}^{\left(t\right)}$$ [1]

where *x*(*t*) is the environment value faced in generation *t*, and *λ*_*k*_(*x*) the expected number of live offspring produced by lineage *k* when faced with the environment *x*. The function *λ*_*k*_(*x*) thus defines the fitness reaction norm for lineage *k*.

Following the genotyping setup, the genome is divided into *L* non-overlapping regions, and we refer to the haplotype in a region as a region-wide haplotype (RWH). A “lineage” *k* is described by a tuple *S*_*k*_, indicating the RWHs in each region, such that *S*_*k*_ = (*l*_*k*,1_,*l*_*k*,2_,⋯,*l*_*k*,*L*_), and where *l*_*k*,*i*_ is the RWH located in region *i* in lineage *k*. We assume that the fitness reaction norm of a lineage is an additive function of the fitness reaction norms of the RWHs in that lineage such that:

$$\xi_{k}(x)=\log\left(\lambda_{k}(x)\right)=\log\left(\lambda (x|\Theta ,S_{k})\right)=\sum_{l\in S_{k}}f(x|\theta_{l}),f(x|\theta_{l})\in R$$ [2]

where Θ is a vector of parameters for the region-wide haplotypes, *θ*_*l*_ the parameters for RWH *l*, and *f*(*x*|*θ*_*l*_) the parametric function describing the fitness reaction norm for a single RWH. We here consider *f*(*x*|*θ*_*l*_) to be a linear *f*(*x*|*θ*_*l*_) = *a*_*l*_*x* + *b*_*l*_, such that *θ*_*l*_ = (*a*_*l*_,*b*_*l*_) or quadratic function *f*(*x*|*θ*_*l*_) = *a*_*l*_*x*^2^ + *b*_*l*_*x* + *c*_*l*_, such that *θ*_*l*_ = (*a*_*l*_,*b*_*l*_,*c*_*l*_) of the environmental value *x*.

Given genotyping data at *H* time-points plus the ancestral, we consider distinct epochs of the experimental evolution, evaluated at generations *T*_0_,*T*_1_,⋯,*T*_*H*_ (such that *T*_0_ = 0, *T*_1_ = 10, *T*_2_ = 35 and *T*_3_ = 50). To denote the epoch to which a certain variable corresponds, a superscript inside square brackets is used. For a single population, the frequency of lineage *k* in epoch *h*, denoted by $g_{k}^{\left[h\right]}$, follows from the frequencies of the lineages in the previous epochs:

$$g_{k}^{\left[h\right]}\propto \exp\left(\sum_{t=1+T_{h-1}}^{T_{h}}\xi_{k}(x(t))\right)g_{k}^{\left[h-1\right]},\;h=1,2,\cdots ,H$$ [3]

where *x*(*t*) is the environment faced in generation *t*.

The ancestral population, consisting of *G li*neages, is described by two variables: *A* = (*S*_1_,*S*_2_,⋯,*S*_*G*_), corresponding to the RWHs present in each lineage; and $g^{\left[0\right]}=(g_{1}^{\left[0\right]},g_{2}^{\left[0\right]},\cdots ,g_{G}^{\left[0\right]})$, specifying the frequency of each lineage (such that $\sum_{k=1}^{G}g_{k}^{\left[0\right]}=1$).

### Inference of lineage fitness reaction norms

For inferring the lineage fitness reaction norms, *λ*_*k*_(*x*), we consider that *A* and *g*^[0]^ are known. Since this is not the case in the analysis of the experimental data, we sample the pair (*A*,*g*^[0]^), given the experimental data, and then estimate the RWH parameters Θ, repeating these two steps multiple times (sections 1.7.6 and 1.7.7 of the S1 Text).

Under the population genetics model used, all replicate populations within a single evolutionary regime *c* have the same dynamics of the lineage frequencies $g_{k}^{\left[h\right]}$. Let $X_{c}=(X_{c}^{\left[1\right]},X_{c}^{\left[2\right]},\cdots ,X_{c}^{\left[H\right]})$ denote the sequence of environmental values in regime *c*, where $X_{c}^{\left[h\right]}=\left(x\;(t_{1}^{\left[h\right]}),x\;(t_{2}^{\left[h\right]}),\cdots ,x\;(t_{T_{h}-T_{h-1}}^{\left[h\right]})\right),\;t_{i}^{\left[h\right]}=i+T_{h-1}$. Inference is framed in a maximum likelihood context, with contributions from each evolutionary regime, given the fitness and genotyping data. We consider without loss of generality that fitness and genotyping data are available for all epochs *T*_0_,*T*_1_,⋯,*T*_*H*_ for each regime. The case in which data is available only for certain epochs is treated by evaluating the corresponding likelihood function only for those epochs. The S1 Text details how the input data, at the level of the replicate populations, is converted to that at the level of each regime.

Let $W_{c}=(W_{c,1},W_{c,2},\cdots ,W_{c,N_{E}})$ denote the fitness data on regime *c*, with *N*_*E*_ assay environments, with *x*_*m*_ being the environmental value, and $\phi_{c,m}^{\left[h\right]}$ the observed population-averaged fitness value of a population from regime *c* in epoch *h* in the *m*–th assay environment. We assume a log-normal model for noise in the observed values $\phi_{c,m}^{\left[h\right]}$. The log-likelihood for the RWH parameter vector Θ given the fitness data on regime *c* is then:

$$L_{W}(\Theta |W_{c},X_{c},A,g^{\left[0\right]})\propto -\sum_{h=0}^{H}\sum_{m=1}^{N_{E}}{log}^{2}\left(\frac{1}{\phi_{c,m}^{\left[h\right]}}\sum_{k=1}^{G}\lambda_{k}(x_{m})g_{k}^{\left[h\right]}\right)$$ [4]

Let $D_{c}=(D_{c}^{\left[1\right]},D_{c}^{\left[2\right]},\cdots ,D_{c}^{\left[H\right]})$ be the genotyping data on regime *c* (note that it does not include the data on the ancestral), such that $D_{c}^{\left[h\right]}=(n_{{c,l}_{1}}^{\left[h\right]},n_{{c,l}_{2}}^{\left[h\right]},\cdots ,n_{{c,l}_{M}}^{\left[h\right]})$, where $n_{c,l}^{\left[h\right]}$ is the number of copies of RWH *l* that were observed in epoch *h* in regime *c*. Then, the log-likelihood given the genotyping data on regime c is given by:

$$L_{D}(\Theta |D_{c},X_{c},A,g^{\left[0\right]})\propto \sum_{h=1}^{H}\sum_{l}n_{c,l}^{\left[h\right]}\;log\;\left(\sum_{k=1}^{G}I(l,S_{k})g_{k}^{\left[h\right]}\right)$$ [5]

where $I(l,S_{k})$ is an indicator function, equal to 1 if lineage *k* has RWH *l*, or equal to 0 otherwise.

Considering all evolutionary regimes *C*, the log-likelihood is then obtained by combining Eqs [4] and [5]:

$$\sum_{c\in C}L_{W}(\Theta |W_{c},X_{c},A,g^{\left[0\right]})+L_{D}(\Theta |D_{c},X_{c},A,g^{\left[0\right]})$$ [6]

Model fitting is then performed by maximizing Eq [6], using a gradient-based optimization algorithm, starting from random initial conditions.

### Archiving

All data and code for analysis has been archived in *Dryad*.*org*: doi:10.5061/dryad.76n6f7c. The archive consists of the following sets of files, each with a README.md for instructions on setting up the analysis and running the code: 1) input_data-genotp_data_NaCl.zip: raw genotyping data on the initial NaCl experiment (50 generations); 2) analysis_code-genotp_data_NaCl.zip: R code for preparing and summarizing the genotyping data on the initial NaCl experiment in changing environments. 3) input_data-growth_rate_data_NaCl.zip: raw growth-rate data on the ancestral population and the lines L28 and L11; 4) analysis_code-growth_rate_data_NaCl.zip: R code for the analysis of the growth-rate data. This is necessary for inference of the RWH parameters and the lineages, since the inference relies on fitness data on the ancestral population. 5) analysis_code-inferring_RWH_params.zip: R code for the inference of RWH parameters and the lineages, given the genotyping data during the NaCl experiment and the fitness data on the ancestral. 6) analysis_results.zip: the overall results of the analysis in the paper, which was the source for the figures; 7) input_data-genotp_data_NaCl_continuation.zip: raw genotyping data for the second set of experiments (30 generations); 8) analysis_code-genotp_data_NaCl_continuation.zip: R code for the analysis of the data on the second set of experiments.

## Supporting information

S1 Text Detailed description of materials and methods. (PDF)

S1 Fig Overview of the genotyping data for analysis. (A) Histograms showing the distance between consecutive SNPs in each of the six chromosomes after quality control. Top figure shows physical distance (in kbp, see [53] for gene sizes and densities in *C*. *elegans*), and bottom figure shows genetic distances (in cM, based on *C*. *elegans* genetic map of [24], each chromosome with 50cM). (B) Number of individuals for which data is available within the ancestral population (M00) and within each evolved replicate population in the respective time-points; note that gradual population GM2 was not genotyped at generation 50. The numbers shown correspond to those obtained after quality control of the data. For the ancestral population, the sample size is around 3 times larger that of the evolved replicate populations.
(PNG)

S2 Fig Observed RWH frequencies. (A) Most of the RWHs observed across all time points and population sampled are relative rare. Each panel shows the histogram with number of RWH relative to all those observed for each sub-genomic region (left to right). (B) Outline of the approach for defining the major and minor RWHs in a given replicate population; data shown for the gradual population GM1). To the left of the vertical dashed line, all those RWH that were grouped into a single “background” RWH (named H0A, H0B or H0C).
(PNG)

S3 Fig Pre-processing of the RWH data to be used for inference of the lineage fitness reaction norms. (A) Shown is the number of RWHs classified as major and minor as a function of the number of top RWHs selected per sample. (B) Shown is the maximum observed frequency for the minor RWHs as a function of number of top RWHs selected per sample. For the selected value of 3 top RWHs per sample, the maximum frequency is around 10%. (C) For each replicate population, the RWHs are ranked based on the maximum observed frequency across the generations, with the ones attaining the highest maximum frequencies within each region being defined as primary RWHs. Results are shown for replicate population GM1, with the top 3 RWHs being defined as primary RWHs; H0A, H0B and H0C correspond to the background RWHs in the respective regions.
(PNG)

S4 Fig Number of possible lineages segregating in the ancestral population. Number of lineages obtained as a function of the number of top RWHs selected as primary RWHs within each replicate population, and the number of secondary lineages sampled per replicate population.
(PNG)

S5 Fig Detailed RWH frequency trajectories in region 1 with linear fitness reaction norms. The observed (in part from Fig 3) and predicted frequencies of selected RWHs are shown as a function of the number of generations, in each of the replicate populations. RWHs are shown for region 1 (chromosomes I and II). Predicted frequencies when modeling linear frequency reaction norms are shown for 100 generations (assuming that the gradual populations would be kept at high salt after generation 35). Colored lines correspond to the predicted frequencies for each of the 20 sampled ancestral populations considered for inference. For simplicity, the figures show only the RWHs that are among the 2 RWHs having the largest frequencies per replicate population, considering the observed or predicted frequencies. Each RWH is shown in a single row of a given figure, while each column corresponds to a replicate population. Note that, if a given RWH has not been observed in a replicate population, only the predicted frequencies are shown.
(PNG)

S6 Fig Detailed RWH frequency trajectories in region 2 with linear fitness reaction norms. As in S5 Fig, but for RWHs in region 2 (chromosomes III and IV).
(PNG)

S7 Fig Detailed RWH frequency trajectories in region 3 with linear fitness reaction norms. As in S5 Fig, but for RWHs in region 3 (chromosomes V and X).
(PNG)

S8 Fig Main lineage frequency trajectories. Shown are the predicted frequencies of the main lineages from generation 0 to generation 100 in each of the regimes (as in Fig 5B). Each colored line corresponds to the predicted frequencies for a given sampled ancestral population. For each sampled ancestral population, the lineages within each treatment were ranked based on the maximum frequency observed across the 100 generations. The top 3 lineages per case were then considered, and the frequency of ancestral populations in which each lineage appeared among the top 3 lineages was computed. Note that lineages 1 and 3 correspond, respectively, to L28 and L11. Each main lineage is shown in a single column, while each row corresponds to an experimental evolution regime (assuming gradual population would be kept in high salt conditions from generation 35 onwards).
(PNG)

S9 Fig RWH frequency trajectories when considering quadratic fitness reaction norms. (A-C) Left columns show that the majority of haplotypes are quickly selected against in all experimental evolution regimes (as in Fig 3, background haplotypes H0#)). Middle and right columns show the two specific haplotypes in region 1 (A), region 2 (B) or region 3 (C) showing the greatest frequency change until generation 50. Dots and error bars are the mean and one standard error of the observed haplotype frequencies among replicate populations (same as in Fig 3). Lines show the inferred trajectories when RWH fitness reaction norms are modeled as quadratic, instead of linear as in Fig 3. Shaded grey area corresponds to the trajectories obtained by sampling the ancestral 20 times.
(PNG)

S10 Fig Quadratic fitness reaction norms of the two lineages explaining population genetic dynamics. (A) Predicted fitness reaction norms of the L28 (red) and L11 (blue), as in Fig 5A, but modeling quadratic functions. Each line corresponds to one sample done of the ancestral population. (B) Expected L28 and L11 frequency trajectories for 100 generations (with the gradual populations being kept at high salt after generation 35). Shaded colors show the deterministic expectations based on simulating quadratic fitness reaction norms and sampling the ancestor population 20 times, with the line showing the median.
(PNG)

S11 Fig Expected population fitness with linear reaction norms. The average population fitness and variance in fitness for 20 samples of the ancestral population, under the sudden or the gradual regimes, when modeling linear reaction norms for each segregating lineage. Lines indicate the mean with shaded areas the 95% credible interval.
(PNG)

S12 Fig Expected population fitness with quadratic reaction norms. The average population fitness and variance in fitness for 20 samples of the ancestral population, under the sudden or the gradual regimes, when modeling quadratic reaction norms for each segregating lineage. Lines indicate the mean with shaded areas the 95% credible interval.
(PNG)

S13 Fig Statistics on the RWHs observed on the lines derived from replicate populations GM1 and GM3 in generation 50. (A) 100 lines were derived from gradual populations (GM1 and GM3) at generation 50 and whole-genome sequenced in [21]. Most of the CWHs and RWHs observed in the present study are observed as well in the lines of [21]. Shown are the number of distinct CWHs and RWHs observed within each region, considering the lines derived from each replicate population (rows 1 and 2), and considering all lines (last row). (B) RWHs detected in high frequencies in the lines were also observed in the populations, having been assigned most often as major RWHs. (C) Overview of the lineages corresponding to the lines that were derived out of replicate populations GM1 and GM3 in generation 50. Shown are the number of lines corresponding to each lineage, considering each replicate population (rows 1 and 2), and both altogether (last row). The lineages are labelled based on their rank in terms of the frequency within the replicate populations. Lineages G4 and G9 correspond to L11 and L28 in [21] (see also S2 Table), respectively, and were observed in lines derived from both replicate populations.
(PNG)

S14 Fig Calibration curves for the pooled sequencing data. Pooled sequencing data was used to estimate lineage frequencies in the head-to-head competition assays (Fig 6D) and in the second set of evolution experiments (Fig 7). Each entry corresponds to a single SNP analyzed, with the symbols corresponding to the observed values, while the curves correspond to the fitted calibration curves over known ratio of L28 gDNA molarity versus L11 gDNA molarity (magenta) or known ratio of L28 gDNA molarity versus the ancestral population (M00) gDNA molarity (green). The former data has been omitted in the case that L28 and L11 have the same allele for the respective SNP.
(PNG)

S15 Fig Differentiating L28 with pool genotyping. Impact of a subset of SNPs in terms of the ability to distinguish all possible segregating lineages (S4 Fig), contrasting the effect of considering the 29 target SNPs that were assayed in the pooled genotyping data (Fig 6D and Fig 7) with the full set of 761 SNPs used in the first set of evolution experiments. Shown is the number of lineages confounded with the target lineage (L28) as a function of the number of different SNPs (compared with L28). The figure is shown in a logarithmic scale in both axes. The number of lineages matching a threshold value lower than 1 corresponds to the lowest number of confounded lineages: 1 lineage, for all 761 SNPs (L28), and 10 lineages for the 29 target SNPs (L28 and 9 other lineages).
(PNG)

S16 Fig Discrepancy L28 function. Shown are the discrepancy values for each ancestral population used to seed the continued experimental evolution under each of the experimental evolution regimes (large or small population size); (A) for populations where L28 was lost before continued experimental evolution, (B) for the population where L28 swept in large populations, (C) for the populations where L28 swept in some of the small populations and (D) where L28 swept regardless of population size.
(PNG)

S17 Fig Probability of a sweep involving lineage L28. Estimated probability of a L28 sweep for each ancestral population used to seed the continued experimental evolution under each of the evolutionary regime (large or small population size); (A) for populations where L28 was lost before continued experimental evolution, (B) for the population where L28 swept in large populations, (C) for the populations where L28 swept in some of the small populations and (D) where L28 swept regardless of population size.
(PNG)

S1 Table Experimental evolution populations analyzed in the present study. (TXT)

S2 Table Lineage identification among the 100 lines of ref. [21]. (PDF)

## References

[1] Stocker TF, D. Qin G.-K. Plattner L.V. Alexander S.K. Allen N.L. Bindoff, et al Technical Summary Chapter 02. In: Climate Change 2013: The Physical Science Basis Contribution of Working Group I to the Fifth Assessment Report of the Intergovernmental Panel on Climate Change. Cambridge: Cambridge University Press; 2013.

[2] Lynch M, Lande R. Evolution and extinction in response to environmental change In: Kareiva P, Kingsolver JG, Huey RB, editors. Biotic Interactions and Global Change. Sunderland, MA: Sinauer; 1993.

[3] Chevin LM, Lande R, Mace GM. Adaptation, plasticity, and extinction in a changing environment: towards a predictive theory. PLoS biology. 2010;8(4):e1000357 10.1371/journal.pbio.1000357 ; PubMed Central PMCID: PMC2864732. 20463950 PMC2864732

[4] Kopp M, Hermisson J. The genetic basis of phenotypic adaptation I: fixation of beneficial mutations in the moving optimum model. Genetics. 2009;182(1):233–49. 10.1534/genetics.108.099820 ; PubMed Central PMCID: PMC2674819. 19255369 PMC2674819

[5] Lande R. Adaptation to an extraordinary environment by evolution of phenotypic plasticity and genetic assimilation. Journal of evolutionary biology. 2009;22(7):1435–46. 10.1111/j.1420-9101.2009.01754.x . 19467134

[6] Matuszewski S, Hermisson J, Kopp M. Fisher's geometric model with a moving optimum. Evolution. 2014;68(9):2571–88. 10.1111/evo.12465 PubMed Central PMCID: . 24898080 PMC4285815

[7] Bell G, Gonzalez A. Adaptation and evolutionary rescue in metapopulations experiencing environmental deterioration. Science. 2011;332(6035):1327–30. 10.1126/science.1203105 . 21659606

[8] Collins S, de Meaux J. Adaptation to different rates of environmental change in Chlamydomonas. Evolution. 2009;63(11):2952–65. 10.1111/j.1558-5646.2009.00770.x . 19619223

[9] Perron GG, Gonzalez A, Buckling A. The rate of environmental change drives adaptation to an antibiotic sink. Journal of evolutionary biology. 2008;21(6):1724–31. 10.1111/j.1420-9101.2008.01596.x . 18681913

[10] Gorter FA, Aarts MGM, Zwaan BJ, de Visser JA. Dynamics of adaptation in experimental yeast populations exposed to gradual and abrupt change in heavy metal concentration. The American naturalist. 2015;187:110–9. 10.1086/684104 27277407

[11] Matuszewski S, Hermisson J, Kopp M. Catch me if you can: adaptation from standing genetic variation to a moving phenotypic optimum. Genetics. 2015;200:1255–74. 10.1534/genetics.115.178574 26038348 PMC4574244

[12] Hill WG. Rates of change in quantitative traits from fixation of new mutations. Proceedings of the National Academy of Sciences of the United States of America. 1982;79(1):142–5. Epub 1982/01/01. . 6948296 10.1073/pnas.79.1.142 PMC345678

[13] Walsh B, Lynch M. Evolution and Selection of Quantitative Traits. nitro.biosci.arizona.edu/zbook/NewVolume_2/newvol2.html2014.

[14] Gonzalez A, Bell G. Evolutionary rescue and adaptation to abrupt environmental change depends upon the history of stress. Philosophical transactions of the Royal Society of London Series B, Biological sciences. 2013;368(1610):20120079 10.1098/rstb.2012.0079 ; PubMed Central PMCID: PMC3538446. 23209161 PMC3538446

[15] Chelo IM, Nédli J, Gordo I, Teotónio H. An experimental test on the probability of extinction of new genetic variants. Nature Communications. 2013;4: 10.1038/ncomms3417 24030070 PMC3778522

[16] Fisher R. The Genetical Theory of Natural Selection Oxford: Oxford University Press; 1930.

[17] Price GR. Selection and covariance. Nature. 1970; 227(5257):520–1. 5428476 10.1038/227520a0

[18] Theologidis I, Chelo IM, Goy C, Teotónio H. Reproductive assurance drives transitions to self-fertilization in experimental Caenorhabditis elegans. BMC biology. 2014;12(1):93 10.1186/s12915-014-0093-1 25369737 PMC4234830

[19] Teotónio H, Carvalho S, Manoel D, Roque M, Chelo IM. Evolution of outcrossing in experimental populations of Caenorhabditis elegans. PloS one. 2012;7(4):e35811 10.1371/journal.pone.0035811 ; PubMed Central PMCID: PMC3335146. 22540006 PMC3335146

[20] Chelo IM, Teotónio H. The opportunity for balancing selection in experimental populations of Caenorhabditis elegans. Evolution. 2013;67(1):142–56. 10.1111/j.1558-5646.2012.01744.x . 23289568

[21] Noble L, Chelo IM, Guzella T, Afonso B, Riccardi D, Ammerman P, et al Polygenicity and epistasis underlie fitness-proximal traits in the Caenorhabditis elegans multiparental experimental evolution (CeMEE) panel. Genetics. 2017;207(4):1663–85. 10.1534/genetics.117.300406 29066469 PMC5714472

[22] Crow JF, Kimura M. An Introduction to Population Genetics Theory New York: Harper & Row, Publishers; 1970.

[23] Teotónio H, Estes S, Phillips P, Baer CF. Evolution experiments with Caernohabditis nematodes. Genetics. 2017;206(12):691–716. 10.1534/genetics.115.186288 28592504 PMC5499180

[24] Rockman MV, Kruglyak L. Recombinational landscape and population genomics of Caenorhabditis elegans. PLoS genetics. 2009;5(3):e1000419 10.1371/journal.pgen.1000419 . 19283065 PMC2652117

[25] Burger R. The Mathematical Theory of Selection, Recombination, and Mutation Levin S, editor. New York: John Wiley & Sons, Ltd; 2000.

[26] Gallet R, Cooper TF, Elena SF, Lenormand T. Measuring selection coefficients below 10(-3): method, questions, and prospects. Genetics. 2012;190(1):175–86. 10.1534/genetics.111.133454 ; PubMed Central PMCID: PMC3249376. 22042578 PMC3249376

[27] Gerrish PJ, Lenski RE. The fate of competing beneficial mutations in an asexual population. Genetica. 1998;102/103:127–44. 10.1023/a:1017067816551 9720276

[28] Hill WG, Robertson A. The effect of linkage on limits to artificial selection. Genet Res. 1966;8(3):269–94. . 5980116

[29] Barton NH. Linkage and the limits to natural selection. Genetics. 1995;140(2):821–41. Epub 1995/06/01. . 7498757 10.1093/genetics/140.2.821 PMC1206655

[30] Schlotterer C, Kofler R, Versace E, Tobler R, Franssen SU. Combining experimental evolution with next-generation sequencing: a powerful tool to study adaptation from standing genetic variation. Heredity. 2014;0. 10.1038/hdy.2014.86 . 25269380 PMC4815507

[31] Desai MM. Statistical questions in experimental evolution. Journal of Statistical Mechanics: Theory and Experiment. 2013;2013(01):P01003 10.1088/1742-5468/2013/01/p01003

[32] Illingworth CJR, Mustonen V. Quantifying selection in evolving populations using time-resolved genetic data. Journal of Statistical Mechanics: Theory and Experiment. 2013;2013(01):P01004 10.1088/1742-5468/2013/01/p01004

[33] Martin G, Aguilee R, Ramsayer J, Kaltz O, Ronce O. The probability of evolutionary rescue: towards a quantitative comparison between theory and evolution experiments. Philosophical transactions of the Royal Society of London Series B, Biological sciences. 2013;368(1610):20120088 10.1098/rstb.2012.0088 ; PubMed Central PMCID: PMC3538454. 23209169 PMC3538454

[34] Nene NR, Dunham AS, Illingworth CJR. Inferring Fitness Effects from Time-Resolved Sequence Data with a Delay-Deterministic Model. Genetics. 2018;209(1):255–64. 10.1534/genetics.118.300790 . 29500183 PMC5937181

[35] Neher RA, Shraiman BI. Competition between recombination and epistasis can cause a transition from allele to genotype selection. Proceedings of the National Academy of Sciences of the United States of America. 2009;106(16):6866–71. 10.1073/pnas.0812560106 ; PubMed Central PMCID: PMC2672512. 19366665 PMC2672512

[36] Kryazhimskiy S, Rice DP, Jerison ER, Desai MM. Microbial evolution. Global epistasis makes adaptation predictable despite sequence-level stochasticity. Science. 2014;344(6191):1519–22. 10.1126/science.1250939 . 24970088 PMC4314286

[37] Lang GI, Botstein D, Desai MM. Genetic variation and the fate of beneficial mutations in asexual populations. Genetics. 2011;188(3):647–61. 10.1534/genetics.111.128942 ; PubMed Central PMCID: PMC3176544. 21546542 PMC3176544

[38] Wagner A., Robustness evolvability, and neutrality. FEBS Lett. 2005;579(8):1772–8. 10.1016/j.febslet.2005.01.063 . 15763550

[39] Hahn MW, Conant GC, Wagner A. Molecular evolution in large genetic networks: does connectivity equal constraint? Journal of molecular evolution. 2004;58(2):203–11. 10.1007/s00239-003-2544-0 . 15042341

[40] Lindsey HA, Gallie J, Taylor S, Kerr B. Evolutionary rescue from extinction is contingent on a lower rate of environmental change. Nature. 2013;494:463–7. 10.1038/nature11879 . 23395960

[41] Weissman DB, Barton N. Limits to the rate of adaptive substitution in sexual populations. PLoS genetics. 2012;8:e1002740 10.1371/journal.pgen.1002740 22685419 PMC3369949

[42] Lang GI, Rice DP, Hickman MJ, Sodergren E, Weinstock GM, Botstein D, et al Pervasive genetic hitchhiking and clonal interference in forty evolving yeast populations. Nature. 2013;500(7464):571–4. 10.1038/nature12344 ; PubMed Central PMCID: PMC3758440. 23873039 PMC3758440

[43] McDonald MJ, Rice DP, Desai MM. Sex speeds adaptation by altering the dynamics of molecular evolution. Nature. 2016 10.1038/nature17143 . 26909573 PMC4855304

[44] Roze D. Effects of interference between selected loci on the mutation load, inbreeding depression, and heterosis. Genetics. 2015;201:745–57. 10.1534/genetics.115.178533 26269503 PMC4596681

[45] Kamran-Disfani A, Agrawal AF. Selfing, adaptation and background selection in finite populations. Journal of evolutionary biology. 2014;27(7):1360–71. 10.1111/jeb.12343 . 24601989

[46] Stiernagle T. Maintenance of C. elegans Hope IA, editor. Oxford: Oxford University Press; 1999.

[47] R Development Core Team. R: A language and environment for statistical computing R Foundation for Statistical Computing, Vienna, Austria 2015.

[48] Lenth RV. lsmeans: Least-Squares Means. R package version 2.20–23. http://CRANR-projectorg/package=lsmeans. 2015.

[49] Bates D, Maechler M, Bolker B, Walker S. Fitting Linear Mixed-Effects Models Using lme4. Journal of Statistical Software. 2015;67:1–48.

[50] Bradic M, Costa J, Chelo IM. Genotyping with Sequenom In: Orgogozo V, Rockman M, editors. Molecular Methods for Evolutionary Genetics. 772. New York: Humana Press; 2011. 10.1007/978-1-61779-228-1_11 22065439

[51] Scheet P, Stephens M. A fast and flexible statistical model for large-scale population genotype data: applications to inferring missing genotypes and haplotypic phase. Am J Hum Genet. 2006;78(4):629–44. 10.1086/502802 . 16532393 PMC1424677

[52] Elzhov TV, Mullen KM, Spiess A-N, Bolker B. minipack.lm: R interface to the Levenberg-Marquardt nonlinear lesat-squares algorithm found in MINPACK, plus support for bounds. 2016.

[53] Cutter AD, Dey A, Murray RL. Evolution of the Caenorhabditis elegans genome. Molecular biology and evolution. 2009;26(6):1199–234. 10.1093/molbev/msp048 . 19289596

[54] Guzella TS, Dey S, Chelo IM, Pino-Querido A, Pereira VF, Proulx SR, Teotónio H. 2018 Data from: Slower environmental change hinders adaptation from standing genetic variation. Dryad Digital Repository. 10.5061/dryad.76n6f7c PMC6233921 30383789

