# Supplementary material for: Slower environmental change hinders adaptation from standing genetic variation
Source: PLoS Genet. 2018 Nov 1;14(11):e1007731. doi: 10.1371/journal.pgen.1007731 (PMC6233921; doi:10.1371/journal.pgen.1007731)
Supplement: S1 Text — (PDF) [file pgen.1007731.s001.pdf]

Supporting Information for: Slower environmental change hinders adaptation from standing genetic variation

Thiago S. Guzella[1], Snigdhadip Dey[1], Ivo M. Chelo[2], Ania Pino-Querido[2], Veronica Pereira[1], Stephen R. Proulx[3], and Henrique Teotónio[1,*]

[1]*Institut de Biologie de l'École Normale Supérieure (IBENS), École Normale Supérieure, CNRS, Inserm, PSL Research University, F-75005 Paris, France.*

[2]*Instituto Gulbenkian de Ciência, Oeiras P-27801-901, Portugal.*

[3]*Department of Ecology, Evolution, and Marine Biology, University of California Santa Barbara, CA 93106, U.S.A.*

[*]*Correspondence to: teotonio@biologie.ens.fr*

Contents

1 Detailed materials and methods **3**

1.1 Experimental evolution in changing salt environments 3

1.1.1 Ancestral population 3

1.1.2 Design 3

1.2 Identifying the two lineages (L28 and L11) explaining most of population genetics during experimental evolution 3

1.3 Absolute fitness assays 4

1.3.1 Ancestral population 4

1.3.1.1 Design 4

1.3.1.2 Statistical analysis 4

1.3.2 L28 and L11 lineages 5

1.3.2.1 Design 5

1.3.2.2 Statistical analysis 5

1.4 Relative fitness assays between L28 and L11 6

1.4.1 Design 6

1.4.2 Statistical analysis 7

1.5 Experimental evolution at different population sizes in constant high salt 8

1.5.1 Ancestral populations 8

1.5.2 Design 8

1.6 Genotyping of single individuals from the evolution experiments in changing environments . . . 9
1.6.1 Sample preparation . . . 9
1.6.2 Quality control . . . 9
1.7 Pooled genotyping from relative L28/L11 fitness assays and experimental evolution at different population sizes in constant high salt . . . 10
1.7.1 Sample preparation . . . 10
1.7.2 Quality control . . . 10
1.8 Modeling selection in changing environments . . . 12
1.8.1 Preliminary considerations . . . 12
1.8.2 General model . . . 13
1.9 Maximum-likelihood inference of fitness reaction norms . . . 15
1.9.1 Lineage fitness reaction norms given fitness data . . . 15
1.9.2 Lineage fitness reaction norms given genotyping data . . . 17
1.9.3 Summing the max-likelihoods of fitness and genotyping data for the analysis of multiple populations and regimes . . . 18
1.10 Defining major sub-genomic region haplotypes (RWHs) . . . 19
1.11 Sampling standing genetic variation in the ancestral population . . . 19
1.11.1 Lineage identity . . . 19
1.11.2 Estimating the initial lineage frequencies . . . 20
1.12 Adaptation during experimental evolution at different population sizes in constant high salt . . . 21
1.12.1 Inferring the occurrence of an L28 sweep given the pooled genotyping . . . 21
1.12.2 Principal component analysis . . . 22

2 References for supplementary information . . . 23

1 Detailed materials and methods

1.1 Experimental evolution in changing salt environments

1.1.1 Ancestral population

All populations employed here are ultimately derived from a hybrid population of 16 wild isolates (Teotonio et al., 2012; Noble et al., 2017), followed by 140 generations of laboratory domestication to a 4-day non-overlapping life-cycle under partial self-fertilization (selfing) at census sizes of $N = 10^4$ (Teotonio et al., 2012; Chelo and Teotónio, 2013), and finally introgression and homozygosity of the *xol-1(tm3055)* allele at high populations sizes for 16 generations to generate the ancestral population named M00 (where M stands for monoecious and 00 for ancestor). The *xol-1(tm3055)* allele disrupts X-chromosome dosage compensation such that male embryos are not viable (Meyer, 2005), and reproduction occurs exclusively by selfing as hermaphrodites cannot mate each other (Maupas, 1900). Reproduction is discrete within 2 hours of the 96 hour life-cycle. Barring overdominance, homozygosity across the genome should be quickly achieved during the first few generations (Crow and Kimura, 1970).

1.1.2 Design

The experimental evolution design has been detailed elsewhere (Theologidis et al., 2014). M00 samples with more than 10^4 individuals were thawed, expanded in numbers and first larval staged (L1s) seeded at the appropriate densities to three regimes. The "Sudden" experimental evolution regime was characterized by the same 4-day life cycle conditions to which previous lab-adaptation occurred, except that NGM-lite media (US Biological) was supplemented with NaCl to 1.78% w/v (305 mM) from the start of the experiment and for 50 generations (SM2, SM3, SM5, and SM6 populations; where S stands for sudden, M for monoecious). For the "Gradual" regime NGM-lite plates were supplemented with increasing concentrations of NaCl from 33 mM at generation 1 to 305 mM NaCl at generation 35 and onwards until generation 50 (GM1-7 populations). Finally, a "Control" regime was maintained in the ancestral environmental conditions without any salt supplement, also for 50 generations (CM1-3 populations). For all experimental evolution regimes, [NaCl] from L1 to adult reproduction were constant, from embryo to L1 [NaCl] were of 25 mM under all regimes. Periodic samples of each population were cryogenically frozen at high densities. During the experiments we were always able to maintain census population sizes at the L1 seeding stage at 10^4 or above (Theologidis et al., 2014).

1.2 Identifying the two lineages (L28 and L11) explaining most of population genetics during experimental evolution

Our analysis suggested that effectively one lineage swept through the sudden populations, while another lineage was initially sweeping though the gradual populations when they were at intermediate salt concentrations. From the GM1 and GM3 populations at generation 50, we have derived elsewhere (Noble et al., 2017) 100 lineages

by 13 generations of single hermaphrodite self-fertilization, which were both genotyped by the Sequenom methods described below and whole-genome short-read Illumina sequenced, as described in (Noble et al., 2017). Of these, we chose GM350L28 (L28 for simplicity) and GM350L11 (L11) to test our model predictions, as they would correspond to lineages that presumably swept in the sudden and initially in the gradual populations, respectively.

1.3 Absolute fitness assays

1.3.1 Ancestral population

1.3.1.1 Design Fitness of the M00 population was characterized by carrying out a growth rate assay in three NaCl concentrations: 25 mM, 225 mM and 305 mM. The assays involved measurement of per-capita population growth rates over a complete generation, under conditions that closely mimicked those employed during experimental evolution (Teotonio et al., 2012; Theologidis et al., 2014), and that account for potential confounding maternal and grand-maternal environmental effects (Dey et al., 2016; Teotonio et al., 2017).

Specifically, M00 ($> 10^3$) was thawed from frozen stocks and individuals reared for two generations at 25 mM before they were exposed to the three assay NaCl treatments. On the third generation, five Petri dishes per NaCl treatment were seeded with 1000 L1s per plate. These five plates constituted one technical replicate, and there were four of these for each salt treatment. After 66 h, individuals were harvested with M9 isotonic solution into a 15 mL Falcon tube, and exposed to a 1 M KOH:5% NaOCl "bleach" solution (to which only embryos survive), washed three times and suspended in 5 mL of M9 solution. After 16 h, debris of dead larvae and adults were removed and the total number of live L1s in each tube was estimated by scoring the number of L1s in ten 5 μL drops, and by measuring the total M9 volume. The estimated number of L1s was divided by 5000, the number of L1s used in the previous generation to set up each assay replicate, to calculate the per-capita L1-to-L1 growth rate.

1.3.1.2 Statistical analysis In order to obtain the expected number of live offspring in each assay environment (the key variable necessary for analyzing the data from experimental evolution; see equations 35 and 36), the log-transformed per-capita L1-to-L1 growth rate values were analyzed using a linear model with the assay environment as a categorical variable. For this, the assay environment for the i-th measurement is denoted as E_i, and given by: $E_i = 0$, for 25 mM NaCl; $E_i = 1$, for 225 mM NaCl and $E_i = 2$, for 305 mM NaCl. In this way, the 25 mM NaCl condition is taken as the "reference" environment. The model then takes the form:

$$\zeta_i = \beta_0 + \beta_1 \mathbb{I}(E_i, 1) + \beta_2 \mathbb{I}(E_i, 2) \tag{1}$$

where ζ_i is the log-transformed per-capita growth rate value, β_0, β_1 and β_2 are coefficients to be estimated, and $\mathbb{I}(E_i, j)$ is the indicator function:

$$\mathbb{I}(E_i, j) = \begin{cases} 1, & \text{if } E_i = j \\ 0, & \text{otherwise} \end{cases} \tag{2}$$

The data was analyzed in R (version 3.3; R Core Team, 2016), using the following formula to specify the model in the *lm* function:

```
logGrowthRate ~ saltTreat
```

where `logGrowthRate` is the log-transformed per-capita L1-to-L1 growth rate (numerical variable, corresponding to ζ_i in equation 1) and `saltTreat` is the assay environment (identified by the NaCl concentration: 25 mM, 225 mM or 305 mM; categorical variable, corresponding to E_i in equation 1). Least-square estimates of the log-growth rates were obtained using the R package lsmeans (version 2.24; Lenth, 2016), producing the values $\log(\phi_{25\text{mM}})$, $\log(\phi_{225\text{mM}})$ and $\log(\phi_{305\text{mM}})$ (see section 1.9 and equation 34). These values correspond to the logarithm of the expected number of offspring, averaged over the individuals in the ancestral population, for the respective assay environments (25 mM, 225 mM and 305 mM).

1.3.2 L28 and L11 lineages

1.3.2.1 Design For lineages L28 and L11, the same protocol as for the ancestral M00 was used to estimate L1-to-L1 per capita growth rates, but considering 2 consecutive generations (generations 1 and 2), instead of a single generation as was done for M00 (section 1.3.1). The assays were done over three blocks, each defined by when the L28 and L11 were revived from frozen stocks. In each of the first two blocks, four technical replicates under each lineage and salt treatment were done. In the third block, only L28 was assayed, with four technical replicates under each NaCl concentration treatment.

1.3.2.2 Statistical analysis Let $\lambda_{k,\mathcal{E}}$ denote the expected number of live offspring for lineage k in environment $\mathcal{E}$. Since the data was collected on two generations, we sought to account for the potential presence of trans-generational effects, and obtain estimates of the values of $\lambda_{k,\mathcal{E}}$ for lineages L28 and L11, in 25 mM, 225 mM and 305 mM. We let ζ_i denote the log-transformed number of live offspring obtained in the i-th measurement (i.e., $\zeta_i = \log(\lambda_i)$). The data was analyzed using a mixed effects model, with environment, line and trans-generation component as fixed effects, and "assay block" as a random effect:

$$\zeta_i = \underbrace{\beta(E_i, L_i) + \alpha(L_i)\, g_i}_{\text{Fixed effects}} + \underbrace{\gamma(B_i)}_{\text{Random effect}} \tag{3}$$

where ζ_i is the logarithm of the growth rate for the i-th measurement, E_i denotes the environment ($E_i = 0$ for 25 mM, $E_i = 1$ for 225 mM, and $E_i = 2$ for 305 mM NaCl), L_i denotes line (L11 or L28; $L_i = 0$ for L28, and $L_i = 1$ for L11), g_i denotes the trans-generational component, and B_i is the assay block ($B_i \in \{1, 2, 3\}$).

In particular, g_i is given by:

$$g_i = \left(\frac{c_i - 25}{305 - 25}\right)(t_i - 1) \tag{4}$$

where c_i is the NaCl concentration in mM, and $t_i \in \{1, 2\}$ the generation assayed.

The terms of the model are given by:

- $\beta(E_i, L_i)$ denotes the statistical interaction between environment and line:

$$\beta(E_i, L_i) = \beta_0 + \beta_1 \mathbb{I}(E_i, 1) + \beta_2 \mathbb{I}(E_i, 2) + \beta_3 \mathbb{I}(L_i, 1) + \beta_4 \mathbb{I}(E_i, 1) \mathbb{I}(L_i, 1) + \beta_5 \mathbb{I}(E_i, 2) \mathbb{I}(L_i, 1) \quad (5)$$

  with β_k, $k = 0, 1, \ldots, 5$, being coefficients to be estimated

- $\alpha(L_i)$ denotes the line-dependent trans-generational effect:

$$\alpha(L_i) = \alpha_0 + \alpha_1 \mathbb{I}(L_i, 1) \quad (6)$$

  with α_0 and α_1 being coefficients to be estimated

- intercept-based effect of block:

$$\gamma(B_i) = \sum_{j=1}^{3} \gamma_j \mathbb{I}(B_i, j) \quad (7)$$

  with γ_j, $j \in \{1, 2, 3\}$, being coefficients to be estimated

The model was fit using package lme4 (version 1.1-12; Bates et al., 2015) in R via the following formula:

```
logGrowthRate ~ saltTreat * line + tgenComp * line + (1 | block)
```

where: i) `logGrowthRate` is the log-transformed the per-capita L1-to-L1 growth rate; ii) `saltTreat` is a categorical variable denoting the environment, defined by the salt concentration (25 mM, 225 mM or 305 mM); iii) `line` is a categorical variable denoting the line (L28 or L11); iv) `tgenComp` is a numerical variable representing the "transgenerational component", given by the product between x, the normalized NaCl concentration (given by $x = (c - 25)/(305 - 25)$, where c is the NaCl concentration in mM) and $t - 1$ (where t denotes the generation assayed); v) `block` is a categorical variable representing the assay block (B_1, B_2 or B_3).

Afterwards, the R package lsmeans (version 2.24; Lenth, 2016) was used to obtain two sets of estimates, via the respective R formulas:

- least-squares estimates of $\log(\lambda_{k,\mathcal{E}})$ for each of the two lineages in each of the three NaCl concentrations assayed: `~ saltTreat * line`

- estimates of the selection coefficient of L28 relative to L11, in each of the three NaCl concentrations assayed: `pairwise ~ line | saltTreat`

In both cases, the estimates obtained do not include contributions of trans-generational effects, by evaluating the model at `transgenComp = 0`.

1.4 Relative fitness assays between L28 and L11

1.4.1 Design

L28 and L11 were revived from frozen stocks and reared for two generations at 25 mM NaCl before they were set up for head-to-head competition assays at three NaCl concentrations: 25 mM, 225 mM and 305 mM. On the third generation, L1 larvae

from the two lineages were mixed in 1:1 ratio, at a density of 1000 L1s in each of two Petri dishes per technical replicate. Each technical replicate was then maintained for two generations by employing the protocol used for the aforementioned non-competitive growth rate assays. At both the assay generations, L1 samples ($> 10^3$) were collected for pool-genotyping. Assays were performed in three blocks, with 3 replicates per salt concentration in each of two blocks, and 4 replicates in the third block. Therefore, a total of 10 replicate competitions were done for each experimental treatment (NaCl concentration), with a total of 30 replicates over the entire experiment.

1.4.2 Statistical analysis

The data for analysis was based on the L28 and L11 frequency values obtained after doing several calibration curves where the ratio of both lines was known. Let p_i denote the measured frequency for the L28 allele in the i-th measurement made. This frequency was forced to be always contained in the interval $(0.005, 0.995)$. Moreover, let E_i denote the experimental treatment applied to the i-th measurement: $E_i = 0$ for the low salt (25 mM) environment, $E_i = 1$ for the intermediate salt (225 mM) environment, $E_i = 2$ for the high salt (305 mM) environment. Finally, let t_i denote the generation and $R_i \in \{1, 2, \ldots 30\}$ the replicate population.

Analysis relied on a mixed effects model, with experimental treatment and generation as fixed effects, and "replicate" as a random effect. The model is formulated as:

$$y_i = \underbrace{\beta_0 + \alpha\left(E_i\right) t_i}_{\text{Fixed effects}} + \underbrace{\gamma\left(R_i\right)}_{\text{Random effect}} \tag{8}$$

where y_i is the logarithmic of odds-ratio for the allele of L28:

$$y_i = \log\left(\frac{p_i}{1 - p_i}\right) \tag{9}$$

and the terms of the model are given by:

- β_0 is the intercept, corresponding to the log-odds ratio in the ancestral population. For this term, β_0 is a coefficient to be estimated.

- $\alpha\left(E_i\right)$ is a treatment-dependent coefficient relating the impact of the number of generations:

$$\alpha\left(E_i\right) = \sum_{j=0}^{2} \alpha_j \, \mathbb{I}\left(E_i, j\right) \tag{10}$$

  with the α_k ($k = 0, 1, 2$) being coefficients to be estimated. Note that the selection coefficient of line L28 relative to L11 in assay environments j is therefore given by:

$$s_j = \alpha_j \tag{11}$$

- intercept-based effect of replicate population:

$$\gamma(R_i) = \sum_{j=1}^{30} \gamma_j \mathbb{I}(R_i, j) \tag{12}$$

  with the γ_j ($j \in \{1, 2, \ldots, 30\}$) being coefficients to be estimated

The model was fit using (package lme4, version 1.1-12; Bates et al., 2015) in R via the following formula:

```
logOddsRatioL28Allele ~ generation : saltTreat + (1 | replPop)
```

where: i) `logOddsRatioL28Allele` is the logarithmic of odds-ratio for the allele of L28, given by $\log(p_{\mathrm{L28}}/(1-p_{\mathrm{L28}}))$. ii) `saltTreatment` is a categorical variable denoting the salt concentration; iii) `generation` is a numerical variable denoting the generation; iv) `replPop` is a categorical variable corresponding to the replicate population.

For subsequent analysis, point estimates of the selection coefficients for each SNP in each treatment were obtained.

1.5 Experimental evolution at different population sizes in constant high salt

1.5.1 Ancestral populations

The second set of evolution experiments was conducted at constant 305mM NaCl for 30 generations. All seven replicate GM populations from G35 were revived from frozen stocks at sample sizes of 10^3 individuals or more each, expanded in numbers for two generations, and then split into two regimes: large population sizes of $N = 10^4$ and small population sizes of $N = 2 \cdot 10^3$. For full population nomenclature see S1 table.

1.5.2 Design

For the large population size regime, we followed exactly the same protocol as the first set of evolution experiments, while in the second treatment only 2 Petri dishes were seeded each with 1000 L1s at each generation. From each of the seven gradual populations at generation 35, one replicate was maintained at large population sizes and three replicates were maintained at small population sizes. Given the extent of genetic diversity in the M00 population, the known Poisson distributions of hermaphrodite fertility (Chelo and Teotónio, 2013), and the effects of selfing on the segregation of neutral SNP markers (Crow and Kimura, 1970), we expected the large population size treatment populations to have an effective population size of close to $N_e = 500$, and the small population size treatment populations of about $N_e = 100$. With selection and complete linkage among markers across the genome these numbers will be greatly reduced (Schiffels et al., 2011; Neher, 2013).

1.6 Genotyping of single individuals from the evolution experiments in changing environments

1.6.1 Sample preparation

Immature (L4 larval stage) individuals from M00, SM2, SM3, SM5, SM6, GM1, GM3, GM5, CM1, CM2 and CM3 were hand-picked at generation 10, 35 and 50. GM2 individuals were also collected but only for generation 10 and 35 (as this population was discontinued at this point). Individual genomic DNA was prepared with the ZyGEM prepGEM TM Insect kit following the manufacturer's protocol. A total of 925 biallelic SNPs evenly distributed across the genome according to the genetic distance of Rockman and Kruglyak (2009) were assayed based on the known polymorphism data segregating in the 140 generation lab-adapted population (Noble et al., 2017) and using the iPlex SequenomTM MALDI-TOF methods as described in Bradić et al. (2011). Due to the limited amount of gDNA, each individual was assayed for only two of the six *C. elegans* chromosomes (chromosomes I and II, or III and IV, or V and X). Because of exclusive selfing during experimental evolution, linkage across the genome was expected to be extensive and this design was anticipated to yield a better estimate of the total number of lineages present in the ancestral population, as opposed to sampling fewer SNPs across the whole genome in each individual (see model for inference below, section 1.8). For each one of the three pairs of chromosomes, the genotyping data consists of 64 individuals for the ancestral M00, and 16 individuals per evolutionary replicate population per generation

1.6.2 Quality control

Quality control of the genotyping data on single individuals was performed following Chelo and Teotónio (2013), starting from 817 SNPs that passed preliminary inspection of the raw data. Quality control relied on a large dataset, consisting of data obtained from various populations and experimental evolution done in the lab, so as to leverage a large sample size to discard unreliable SNPs.

In a first step of quality control, we sought to discard SNPs with high frequency of heterozygous calls. For this, we split the various set of samples into two main categories: i) those in which few, if any heterozygous calls were expected, with 4 groups, namely all the monoecious populations considered in the present study (total of 576 individuals genotyped per chromosome), and inbred lines derived from monoecious (100 lines), androdioecious (333 lines) and trioecious populations (88 lines) (Noble et al., 2017); ii) those in which heterozygous calls were expected, with 3 groups, consisting of androdioecious (96 individuals), dioecious (560 individuals) and trioecious (32 individuals) populations (unpublished data). For each of these two categories, the frequency of heterozygous calls was determined as the maximum frequency in the groups belonging to that category. SNPs were then excluded from the analysis if the frequency in both was greater than 5%. The 817 SNPs that passed this first step had at most 10% heterozygous calls in the first category. Heterozygous calls in individuals from the monoecious populations or the inbred lines were then considered missing data.

In the second step of quality control, the frequency of missing data over all individuals and inbred lines genotyped (total of 1728) was determined, and SNPs

having more than 30% of missing data were discarded. Finally, in the last step, individuals/lines having more than 25% of missing data were discarded. In the end, we obtained a total of 761 SNPs that were considered for analysis, with 112 in chromosome I, 119 in II, 130 in III, 134 in IV, 128 in V, and 138 in the X chromosome; median distance between consecutive SNPs within each chromosome are 55-100 kbp and 0.30-0.38 cM The number of individuals that passed quality control per population per pair of chromosomes is shown in figure

After quality control, the genotyping data on all the monoecious populations and the inbred lines was imputed using fastPHASE (version 1.4; Scheet and Stephens, 2006), to obtain chromosome-wide haplotypes. This analysis was done using 20 random starts of the EM algorithm, 10 clusters and using argument `-B` for estimating missing data.

1.7 Pooled genotyping from relative L28/L11 fitness assays and experimental evolution at different population sizes in constant high salt

1.7.1 Sample preparation

For L28 and L11 head-to-head competition assays and for the second set of evolution experiments at different population sizes (see above), we performed genotyping of gDNA obtained from multiple individuals. We decided to do a limited number of SNPs, instead, for example, of doing pooled gDNA whole-genome sequencing, since to estimate allele frequencies we needed as high coverage per SNP site as possible (which is a function of the number of individuals sampled).

GM1-7 at generation 35, and all populations of the second set of evolution experiments after 15 and 30 generations in high salt were collected in pools of L1s ($>10^3$) and gDNA prepared from them using the Qiagen Blood and Tissue kit, following the manufacturer's protocol. These samples were then genotyped for 84 SNPs in chromosomes I, IV and V, using the iPlex Sequenom methods in 3 technical replicates for each SNP assay.

In parallel, pooled gDNA was also prepared for the M00 population and the L28 and L11 lineages. We did two calibration curves in order to estimate SNP allele frequencies. In the first, equal molarity of L28 and L11 gDNA were mixed, while in the second equal molarity of M00 and L28 gDNA, at 0:100, 25:75, 50:50, 75:25 and 100:0 proportions. For the first calibration, between 8 and 14 technical replicates were done for each of the five DNA mixes, while for the second calibration 8 technical replicates per DNA mix were done.

1.7.2 Quality control

For the pooled genotyping, a different quality control approach is needed than that of individual genotyping (Le Hellard et al., 2002). For each SNP, the initial step was verifying whether it has the same or different alleles in L28 and L11. We then considered the calibration data on each SNP, which consists of technical replicates within each calibration value assayed. In these data, we determined the number of technical replicates, within each calibration value, that had frequency values in the interval $(0.05, 0.95)$ (that is, not indicative of fixation). Afterwards, for each

SNP, we summarized the number of "reliable" calibration values as the number of calibration values in which the number of non-fixation values is greater than or equal to 2. We also processed the calibration data to determine, for each SNP and calibration setup (L28 vs L11, or L28 vs M00), the 5%-quantile (denoted by $q_{0.05}$) and the 95%-quantile (denoted by $q_{0.95}$) of the L28 allele frequency values observed, independently of the calibration value. In this way, SNPs that passed the quality control were those that satisfied the following criteria for both calibration settings:

L28 vs L11: if L28 and L11 have the same allele, then the SNP passed the quality control if $q_{0.05} \geq 0.95$. At other frequencies, the SNP passes quality control if the following three conditions are all satisfied: i) $q_{0.05} \leq 0.1$; ii) $q_{0.95} \geq 0.9$; iii) the number of good calibration values is greater than or equal to 2

L28 vs M00: the SNP passed quality control if the following three conditions are all satisfied: i) $q_{0.95} - q_{0.05} \geq 0.2$; ii) $q_{0.95} \geq 0.99$; iii) the number of good calibration values is greater than or equal to 2

This resulted 29 SNPs, out of which 18 have different alleles in L28 and L11.

In order to estimate the true allele frequencies given the measured frequency values, the data on each SNP is denoted as $\mathcal{D} = \{g_1, g_2, \ldots, g_n\}$, with each observation g_i consisting of a tuple (c_i, k_i, y_i), where:

- $0 < c_i < 1$ is the "calibration value", corresponding to the L28 frequency in the mix. This calibration value is pre-processed via $c_i = \min(1 - \delta, \max(\delta, \tilde{c}_i))$, where $\tilde{c}_i$ is the raw, unprocessed value and δ is a threshold, such that $\delta \leq c_i \leq 1 - \delta$, for some $0 < \delta < 1$. This is done so that the *logit* transformation (see equation 15 below) always produces a finite value. For the analysis, we use $\delta = 0.01$.

- $k_i \in \{0, 1\}$ is given by:

$$k_i = \begin{cases} 1, & \text{if } g_i \text{ arises from the calibration data on L28 vs M00} \\ 0, & \text{if } g_i \text{ arises from the calibration data on L28 vs L11} \end{cases} \quad (13)$$

- y_i is the frequency of the L28 SNP allele that was measured

The following formulation was used for fitting of the calibration curves:

$$f_i = c_i + (1 - c_i)\, k_i\, u_0,\;\; 0 < f_i < 1\, \forall i \quad (14)$$

$$z_i = \text{logit}\,(f_i) = \log\left(\frac{f_i}{1 - f_i}\right) \quad (15)$$

$$y_i = \frac{1}{1 + \exp\,(-b\, z_i + d)} + \epsilon_i \quad (16)$$

where f_i (an estimate of the true frequency of the target allele in the sample corresponding to observed g_i) and z_i (transformed version of f_i) is an auxiliary variable. The following parameters were estimated per SNP: i) $u_0 \in [0, 1]$: frequency of the target allele in the ancestral population M00; ii) b: the steepness of the calibration curve; iii) d: the horizontal displacement of the calibration curve.

Model fitting was done using the Levenberg-Marquardt algorithm in R, via function `nlsLM` from package `minpack.lm` (version 1.2-1; Elzhov et al., 2016), considering

100 random initial conditions, and retaining in the end the model with the smallest sum of squared residuals. The parameters were constrained to be in the intervals: $u_0 \in [0.01, 0.99]$, $b \in [0.001, 20]$, and $d \in [-\delta_d, \delta_d]$, where $\delta_d = 5$ for SNPs that are different in L28 and L11, and $\delta_d = 0.1$ for SNPs that are identical in L28 and L11. Once the model was fit, for a given sample and observed value $\tilde{y}_i$, an estimate of the "true" frequency of the target SNP allele with the parameters (u_0, b, d) is obtained by interpolating the calibration curve (f_i, y_i) around the value $\tilde{y}_i$, resulting in the estimate $\tilde{f}_i$.

1.8 Modeling selection in changing environments

1.8.1 Preliminary considerations

In this section we describe a general model to understand natural selection in changing environments solely from extant genetic diversity, which is then applied to infer the population genetic dynamics during the two sets of evolution experiments that we did. We developed this model motivated by two major empirical problems, likely encountered in all studies of natural or experimental populations. First, extant genetic diversity can only be partially accounted for because not all reproducing individuals can be genotyped and/or because genotype data is incomplete with low frequency variants never being sampled or the genetic markers employed being at unknown linkage disequilibrium with the causal adaptive alleles. Second, only partial information about the frequency trajectories of extant genotypes or fitness will be available (e.g., by having data for only one or two time-points during evolution). Related approaches have been used in the context of viral infection or cancer progression, e.g. (Illingworth et al., 2014) and references therein, although environmental change has not been explicitly considered.

We model effectively asexual population genetic dynamics for an haploid organism by ignoring segregation within loci and recombination among loci. We consider a linear genome that may be genotyped at the individual level for bi-allelic markers such as single-nucleotide polymorphims (SNPs).

To explicitly account for how genotyping data was collected (and may be collected in other empirical studies), the genome is divided into L non-overlapping regions, named region-wide haplotypes (RWHs). Each region may correspond to a single SNP allele, a set of SNP alleles in a chromosome or a pair of chromosomes, as in the case of our sampling in the first set of evolution experiments. This allows us to cast all analyses in a multi-allelic framework, thinking of each particular combination of RWHs across the genome as an extant "lineage" of the ancestral population. Finally, we ignore the impact of new mutations, as adaptive ones should be relatively rare and deleterious or neutral ones quickly removed or maintained at low frequencies, respectively, during tens to hundreds of generations (Crow and Kimura, 1970; Matuszewski et al., 2015).

We consider deterministic environmental and population genetic dynamics, although our maximum likelihood inference accounts for measurement errors, random covariates and other non-deterministic effects during experimental evolution and assays (Teotonio et al., 2017).

1.8.2 General model

We assume deterministic population genetic dynamics, under discrete non-overlapping generations and viability selection. By deterministic population genetic dynamics we mean that the frequency of lineages in a given generation is a deterministic function of the frequencies of the previous generation. In addition, we assume an infinite population size, such that any given lineage never goes extinct. Our model further assumes there are no density- or frequency-dependencies, and that trans-generational effects are absent. We let generation 0, $t = 0$, refer to the ancestral population and generation 1, $t = 1$, time at reproduction after populations first faced the respective environment for that generation. The environment here is formalized in terms of an environmental value represented by the variable $x(t)$. In the present study, the environment is the salt concentration, and for convenience we let $x(t)$ denote the normalized salt concentration (with $x(t) = (c(t) - 25)/(305 - 25)$, where $c(t)$ is the concentration in mM). In this way, $x(1) = 0$ for the control populations (25 mM NaCl), $x(1) = 0.11$ for the gradual populations (33 mM NaCl), and $x(1) = 1$ for the sudden populations (305 mM NaCl). Furthermore, we assume that the G lineages that constitute the ancestral population are known in their identity, along with their frequencies.

We define $\lambda_k(x)$ as the fitness reaction norm for a lineage k as a function of the environmental value x. Specifically, $\lambda_k(x)$ corresponds to the expected number of live offspring produced after one generation in the environment x. We account for the environmental values $x(t)$ that define a given experimental evolution regime and for simplicity consider:

$$\lambda_k\left(x(t)\right) = \lambda_k^{(t)} \tag{17}$$

Let $g_k^{(t)}$ denote the frequency of the k-th lineage in generation t, before selection, such that $\sum_{k=1}^{G} g_k^{(t)} = 1 \, \forall t$. Under deterministic dynamics, and for a single population, the frequencies of the lineages in the next generation are then given by (e.g., Bürger, 2000, pp. 24–25):

$$g_k^{(t+1)} = \frac{\lambda_k^{(t+1)} \, g_k^{(t)}}{\sum_{i=1}^{G} \lambda_i^{(t+1)} \, g_i^{(t)}} \tag{18}$$

Since the denominator in equation 18 (which corresponds to the mean population fitness) is merely a scaling factor, ensuring that the lineage frequencies $g_k^{(t+1)}$ add to unity, we will re-write equation 18 with a proportionality sign to simplify the presentation:

$$g_k^{(t+1)} \propto \lambda_k^{(t+1)} \, g_k^{(t)} \tag{19}$$

Extending to after $\delta \geq 1$ generations, one obtains:

$$g_k^{(t+\delta)} \propto \left(\prod_{i=1}^{\delta} \lambda_k^{(t+i)} \right) g_k^{(t)} \tag{20}$$

For H time points plus the ancestral, we consider distinct epochs of the experimental evolution, evaluated at generations $\mathbb{T}_0, \mathbb{T}_1, \ldots, \mathbb{T}_H$. In the present study,

$H = 3$, and $\mathbb{T}_0 = 0$, $\mathbb{T}_1 = 10$, $\mathbb{T}_2 = 35$, and $\mathbb{T}_3 = 50$. Using a superscript enclosed in square brackets to refer to these epochs, we define:

$$\Lambda_k^{[h]} = \prod_{t=\mathbb{T}_{h-1}+1}^{\mathbb{T}_h} \lambda_k^{(t)},\ h = 1, \ldots, H \tag{21}$$

Furthermore, let $g_k^{[h]}$ denote the frequency of the k-th lineage in the h-epoch (i.e., $g_k^{[h]} = g_k^{(\mathbb{T}_h)}$ in our experiments). Using equations 20 and 21, we obtain:

$$g_k^{[h]} \propto \Lambda_k^{[h]}\, g_k^{[h-1]} \tag{22}$$

As the identity of genome-wide haplotypes is never known (due to incomplete characterization of linkage disequilibrium because genotyped markers and causal alleles, insufficient sampling, etc.) the genome can be divided into L non-overlapping regions, called region-wide haplotypes (RWHs). A haploid haplotype or gamete, here termed lineage, k can therefore represented as a tuple $\mathcal{S}_k$ indicating the RWHs in each region:

$$\mathcal{S}_k = (l_{k,1},\, l_{k,2},\, \ldots,\, l_{k,L}) \tag{23}$$

where $l_{k,i}$ is the RWH at the i-th region, such that the ancestral population is represented by:

$$A = (\mathcal{S}_1, \mathcal{S}_2, \ldots, \mathcal{S}_G) \tag{24}$$

To complete the model, we introduce parameters that describe the fitness reaction norm for each of the M RWHs. In this way, the lineage frequencies in the next epoch follow, via equation 22, from the frequencies in the previous epoch, the RWH fitness reaction norm parameters, and the environmental values that are faced in between these two epochs. We let θ_l denote the parameters for RWH l, and:

$$\Theta = (\theta_{l_1}, \theta_{l_2}, \ldots, \theta_{l_M}) \tag{25}$$

denote the parameters for all RWHs. We assume, for simplicity, that the fitness reaction norms for all RWHs have the same parametric form, given by a function $f(\cdot)$. In this way, we write the fitness reaction norm for lineage k as:

$$\lambda_k\,(x) = \lambda\,(x \mid \Theta, \mathcal{S}_k) \tag{26}$$

to emphasize the dependency on the RWH parameters (Θ) and on the RWHs that define lineage k ($\mathcal{S}_k$). Finally, assuming that the fitness reaction norm of lineage k is an additive function, in log-scale, of the fitness reaction norm of the RWHs in that lineage, we define:

$$\log\left(\lambda(x \mid \Theta, \mathcal{S}_k)\right) = \sum_{l \in \mathcal{S}_k} f(x \mid \theta_l) \tag{27}$$

For example, when the log-fitness reaction norms are defined as linear over the environment value x, θ_l would consist of the intercept and the slope. Let $X^{[h]}$ denote the environmental values encountered over an epoch h, that is:

$$X^{[h]} = \left(x\left(t_1^{[h]}\right), x\left(t_2^{[h]}\right), \ldots, x\left(t_{\mathbb{T}_h - \mathbb{T}_{h-1}}^{[h]}\right)\right),\ t_i^{[h]} = i + \mathbb{T}_{h-1} \tag{28}$$

where $x(t)$ is the environmental value encountered in generation t. In this way, over an epoch h, it follows from equation 21 that:

$$\log\left(\Lambda^{[h]} \mid \Theta, \mathcal{S}_k\right) = \sum_{x \in X^{[h]}} \sum_{l \in \mathcal{S}_k} f(x \mid \theta_l), \;\; h = 1, \ldots, H \tag{29}$$

Therefore, equation 22 can be re-written as:

$$g_k^{[h]} \propto \exp\left(\sum_{x \in X^{[h]}} \sum_{l \in \mathcal{S}_k} f(x \mid \theta_l)\right) g_k^{[h-1]}, \;\; h = 1, \ldots, H \tag{30}$$

Hence, equation 30 allows to calculate the frequencies of the lineages in the current epoch h, given the frequencies in the previous epoch $h-1$, the environmental values encountered ($X^{[h]}$) and the lineage fitness reaction norms ($f(x \mid \theta_l)$, $l \in \mathcal{S}_k$).

1.9 Maximum-likelihood inference of fitness reaction norms

In this section, we present the approach for estimating the fitness reaction norm parameters Θ (equation 25) given two types of data, fitness and genotypes. Using model described in section 1.8.2, inference is framed in a maximum likelihood context, such that we derive expressions for the likelihood, and also its gradient with respect to a generic scalar parameter θ. This generic scalar may refer to a parameter of a RWH fitness reaction norm, but also an additional parameter that is introduced in the model for inference.

We first consider the case in which data is available on a single population during the evolution experiment, which encountered environmental values $\mathcal{X} = \left(X^{[1]}, X^{[2]}, \ldots, X^{[h]}, \ldots, X^{[H]}\right)$ (cf. equation 28). In section 1.9.3, we extend the approach to account for the multiple populations and regimes, and combine the likelihood functions on the two types of data into a single function to be optimized for model fitting. Since we assume that the G lineages in the ancestral population are known (A, equation 24), along with their frequencies ($g^{[0]}$), the likelihood functions are conditioned on A and $g^{[0]}$. Furthermore, we consider without loss of generality that both fitness and genotyping data are available for all epochs $\mathbb{T}_0, \mathbb{T}_1, \ldots, \mathbb{T}_H$; the case in which data is available only for certain epochs is treated by evaluating the corresponding likelihood function only for those epochs.

1.9.1 Lineage fitness reaction norms given fitness data

We first consider the fitness data. It may be at the level of single individuals (that is, the number of live offspring produced by each individual sampled) or a population-averaged estimate (e.g., the ratio between the total number of live offspring produced and the total number of individuals sampled). We consider only population-averaged fitness data, with the extension to fitness data at the level of individuals being straight-forward. Moreover, although we use only fitness data on the ancestral population for fitting the model in the present study, for the sake of generality we present expressions considering the general case in which fitness data is gathered in various time-points during experimental evolution. We consider a set of $N_{\mathcal{E}}$ assay environments, with x_m denoting the value corresponding to the m-th assay

environment, and denote the fitness data as:

$$\mathcal{W} = \left(W^{[0]}, W^{[1]}, \ldots, W^{[H]}\right) \tag{31}$$

where the data in epoch h, denoted by $W^{[h]}$, corresponds to data in $N_{\mathcal{E}}$ assay environments:

$$W^{[h]} = \left(\phi_1^{[h]}, \phi_2^{[h]}, \ldots, \phi_m^{[h]}, \ldots, \phi_{N_{\mathcal{E}}}^{[h]}\right) \tag{32}$$

with $\phi_m^{[h]}$ being the population-level fitness that has been measured in the m-th assay environment in epoch h.

Given the lineage frequencies in a given epoch and the parameters of the fitness reaction norms, the predicted population-level fitness in the m-th assay environment is:

$$\widehat{\phi}_m^{[h]} = \sum_{k=1}^{G} \lambda\left(x_m \mid \Theta, \mathcal{S}_k\right)\, g_k^{[h]} \tag{33}$$

We assume for simplicity a log-normal model for noise, such that:

$$\log\left(\phi_m^{[h]}\right) - \log\left(\widehat{\phi}_m^{[h]}\right) \sim \mathcal{N}\left(0, \sigma_w^2\right) \tag{34}$$

where σ_w^2 represents the dispersion between observed and predicted population-level fitness. This dispersion may be taken as either known or to be inferred given the data. Therefore, assuming that σ_w^2 is the same for all assay environments, the log-likelihood for fitness, denoted by $\mathcal{L}_{\mathcal{W}}$, is given by:

$$\mathcal{L}_{\mathcal{W}}\left(\Theta \mid \mathcal{W}, \mathcal{X}, A, g^{[0]}\right) = -\frac{1}{2} \sum_{h=0}^{H} \sum_{m=1}^{N_{\mathcal{E}}} \left[\log\left(2\,\pi\,\sigma_w^2\right) + \frac{1}{\sigma_w^2}\,\left(r_m^{[h]}\right)^2\right] \tag{35}$$

where:

$$r_m^{[h]} = \log\left(\phi_m^{[h]}\right) - \log\left(\widehat{\phi}_m^{[h]}\right) \tag{36}$$

The gradient of the log-likelihood for the fitness data is then given by:

$$\frac{\partial \mathcal{L}_{\mathcal{W}}\left(\Theta \mid \mathcal{W}, \mathcal{X}, A, g^{[0]}\right)}{\partial \theta} = -\frac{1}{2\,\sigma_w^2} \sum_{h=0}^{H} \sum_{m=1}^{N_{\mathcal{E}}} \left[\left(1 - \frac{\left(r_m^{[h]}\right)^2}{\sigma_w^2}\right) \frac{\partial\left(\sigma_w^2\right)}{\partial \theta} + \frac{2\,r_m^{[h]}}{\widehat{\phi}_m^{[h]}} \frac{\partial \widehat{\phi}_m^{[h]}}{\partial \theta}\right] \tag{37}$$

where,

$$\begin{aligned}
\frac{\partial \widehat{\phi}_m^{[h]}}{\partial \theta} &= \sum_{k=1}^{G} \lambda\left(x_m \mid \Theta, \mathcal{S}_k\right) \frac{\partial g_k^{[h]}}{\partial \theta} + g_k^{[h]} \frac{\partial \lambda\left(x_m \mid \Theta, \mathcal{S}_k\right)}{\partial \theta} \\
&= \sum_{k=1}^{G} g_k^{[h]} \frac{\partial \lambda\left(x_m \mid \Theta, \mathcal{S}_k\right)}{\partial \theta} \\
&= \sum_{k=1}^{G} \left(g_k^{[h]}\, \lambda\left(x_m \mid \Theta, \mathcal{S}_k\right) \sum_{l \in \mathcal{S}_k} \frac{\partial f\left(x_m \mid \theta_l\right)}{\partial \theta}\right)
\end{aligned} \tag{38}$$

1.9.2 Lineage fitness reaction norms given genotyping data

We now turn our attention to the individual genotyping data, represented as:

$$\mathcal{D} = \left(D^{[1]}, D^{[2]}, \ldots, D^{[h]}, \ldots, D^{[H]}\right), \; D^{[h]} = \left(n_{l_1}^{[h]}, n_{l_2}^{[h]}, \ldots, n_{l_M}^{[h]}\right) \tag{39}$$

where $n_l^{[h]}$ is the number of times that RWH l has been observed in the h-th epoch. When sampling individuals to be genotyped out of the population in the respective time-point, we assume that the number of observations of the various RWHs follow a multinomial distribution, given the frequencies of the lineages in the population ($g_k^{[h]}, k = 1, \ldots, G$) and the relation between the lineages and the RWHs (A). The assumption of the multinomial distribution is justified when the number of sampled individuals is much smaller than the total number of individuals in the source population, such that the process is well approximated by sampling with replacement. Hence, although the model for inference considers only deterministic population genetics dynamics, it does account for noise due to the sampling of individuals for genotyping. The log-likelihood for the genotyping data is denoted as $\mathcal{L}_{\mathcal{X}}$, and is given by:

$$\mathcal{L}_{\mathcal{D}}\left(\Theta \mid \mathcal{D}, \mathcal{X}, A, g^{[0]}\right) = \sum_{h=1}^{H} \sum_{l} n_l^{[h]} \log\left(\nu_l^{[h]}\right) \tag{40}$$

where $\nu_l^{[h]}$ is the probability of observing RWH l in epoch h (given by the sum of the frequencies of the lineages that have this RWH):

$$\nu_l^{[h]} = \sum_{k=1}^{G} \mathbb{I}\left(l, \mathcal{S}_k\right) g_k^{[h]} \tag{41}$$

$$\mathbb{I}\left(l, \mathcal{S}_k\right) = \begin{cases} 1, & \text{if } l \in \mathcal{S}_k \\ 0, & \text{otherwise} \end{cases} \tag{42}$$

Note that the genotyping data on the ancestral population is not included in $\mathcal{D}$ (equation 39), since the corresponding likelihood term is independent of the reaction norm parameters Θ (as the likelihood would depend only on the initial lineage frequencies $g^{[0]}$).

The gradient of the log-likelihood function for the genotyping data is then:

$$\frac{\partial \mathcal{L}_{\mathcal{D}}\left(\Theta \mid \mathcal{D}, \mathcal{X}, A, g^{[0]}\right)}{\partial \theta} = \sum_{h=1}^{H} \sum_{l} \frac{n_l^{[h]}}{\nu_l^{[h]}} \left(\sum_{k=1}^{G} \mathbb{I}\left(l, \mathcal{S}_k\right) \frac{\partial g_k^{[h]}}{\partial \theta}\right) \tag{43}$$

Defining:

$$\Pi_k^{[h]} = \Lambda_k^{[h]} g_k^{[h-1]}, \; h = 1, 2, \ldots, H \tag{44}$$

$$\Phi^{[h]} = \sum_{k=1}^{G} \Pi_k^{[h]} \tag{45}$$

where $\Lambda_k^{[h]}$ is given by equation 29, the expression for $\frac{\partial g_k^{[h]}}{\partial \theta}$ needed for equation 43 follows from equation 30:

$$\frac{\partial g_k^{[h]}}{\partial \theta} = \frac{1}{\Phi^{[h]}} \left(\frac{\partial \Pi_k^{[h]}}{\partial \theta} - g_k^{[h]} \frac{\partial \Phi^{[h]}}{\partial \theta} \right) \tag{46}$$

$$\frac{\partial \Phi^{[h]}}{\partial \theta} = \sum_{k=1}^{G} \frac{\partial \Pi_k^{[h]}}{\partial \theta} \tag{47}$$

$$\frac{\partial \Pi_k^{[h]}}{\partial \theta} = \Lambda_k^{[h]} \left(\frac{\partial g_k^{[h-1]}}{\partial \theta} + g_k^{[h-1]} \sum_{l \in \mathcal{S}_k} \sum_{x \in X^{[h]}} \frac{\partial f(x \mid \theta_l)}{\partial \theta} \right) \tag{48}$$

1.9.3 Summing the max-likelihoods of fitness and genotyping data for the analysis of multiple populations and regimes

Up to this point we focused on inference in the case of a single evolving population. In this section, we consider how the ensemble of the data that may be collected is analyzed. In the present work, the ensemble of the data consists of multiple experimental evolution regimes, each with several replicate populations. Let the regimes be denoted by $\mathcal{C} = (c_1, c_2, \ldots, c_{N_\mathcal{C}})$, and let $\mathcal{R}_c = (r_1, r_2, \ldots, r_{N_\mathcal{R}})$ define the replicate populations in regime c. Since the replicate populations are independent evolutionary realizations , and in our case the ancestral population is the same in the first set of evolution experiments we did, the log-likelihood for treatment c given the individual genotyping data $\mathcal{D}_c = \left(\mathcal{D}_{r_1}, \mathcal{D}_{r_2}, \ldots, \mathcal{D}_{r_{N_\mathcal{R}}}\right)$ on the $N_\mathcal{R}$ populations is:

$$\sum_{r \in \mathcal{R}_c} \mathcal{L}_\mathcal{D} \left(\Theta \mid \mathcal{D}_r, \mathcal{X}_c, A, g^{[0]} \right) \tag{49}$$

where $\mathcal{X}_c$ denotes the environmental values that define regime c. From equation 40, it follows that the genotyping data from the replicate populations in the evolutionary regime c can be grouped together:

$$\widetilde{\mathcal{D}}_c = \left(\widetilde{D}_c^{[1]}, \widetilde{D}_c^{[2]}, \ldots, \widetilde{D}_c^{[h]}, \ldots, \widetilde{D}_c^{[H]} \right) \tag{50}$$

where:

$$\widetilde{D}_c^{[h]} = \left(\ldots, \sum_{r \in \mathcal{R}_c} n_{l,r}^{[h]}, \ldots \right) \tag{51}$$

with $n_{l,r}^{[h]}$ being the number of times that RWH l was observed in replicate population r in epoch h. We note that, if the sample sizes in some cases are very different, the values of $n_{l,r}^{[h]}$ should be combined considering by weighting, such that cases with large sample sizes do not dominate the log-likelihood; such adjustments were not done in the present study, as the sample size in the experimental data for the evolved populations was mostly between to 12 and 16 individuals per region per sample analyzed, and all replicate populations being analyzed in all epochs (except for GM2 in generation 50).

Therefore, the final log-likelihood for regime c is given by:

$$\mathcal{L}_{\mathcal{W}}\left(\Theta \mid \mathcal{W}_c, \mathcal{X}_c, A, g^{[0]}\right) + \mathcal{L}_{\mathcal{D}}\left(\Theta \mid \widetilde{\mathcal{D}}_c, \mathcal{X}_c, A, g^{[0]}\right) \tag{52}$$

The log-likelihood for the fitness data, $\mathcal{L}_{\mathcal{W}}\left(\Theta \mid \mathcal{W}_c, \mathcal{X}_c, A, g^{[0]}\right)$, is written considering fitness data at the level of the regime c (given $\mathcal{W}_c$), for simplicity. The corresponding values of $\phi_m^{[h]}$ (equation 32) may be obtained out of the values per replicate population by averaging, for example. Finally, the final log-likelihood for the experiment, considering all regimes, is given by:

$$\sum_{c \in \mathcal{C}} \mathcal{L}_{\mathcal{W}}\left(\Theta \mid \mathcal{W}_c, \mathcal{X}_c, A, g^{[0]}\right) + \mathcal{L}_{\mathcal{D}}\left(\Theta \mid \widetilde{\mathcal{D}}_c, \mathcal{X}_c, A, g^{[0]}\right) \tag{53}$$

For model fitting, the L-BFGS gradient-based optimization algorithm was used (Nocedal, 1980; Liu and Nocedal, 1989). Model fitting was done via the NLopt package (version 2.4.2) as implemented in R (version 1.0.4; Johnson, 2017).

1.10 Defining major sub-genomic region haplotypes (RWHs)

We lumped the RWHs that were observed only few times during the experiment into a single RWH, referred to as the background RWH, in order to reduce the number of parameters. In this way, inference is done considering only the major and background RWHs. We call as major RWHs those that are among the K_M RWHs with the highest frequencies in at least one time point and one replicate population that was assayed. For this, the genotyping data on the ancestral population was not considered.

1.11 Sampling standing genetic variation in the ancestral population

1.11.1 Lineage identity

Unless the ancestral population is especially constructed to bear specific lineages at particular frequencies (see, e.g., Gresham et al., 2011), its exact constitution in practice is always unknown. For this reason, we describe the heuristic employed to sample the ancestral population for inference. We describe how to sample A (equation 24), given the genotyping data obtained for the entire experiment and the definition of major and background RWHs. The approach can nonetheless be directly applied to the input RWHs.

Sampling the ancestral population relies on two sets of lineages: i) primary lineages, representing the lineages for which there is reasonable evidence that they are indeed present; ii) secondary lineages, corresponding to those with a lower probability of being present. Consider the genotyping data on a given replicate population r, as initially defined in equation 39:

$$\mathcal{D}_r = \left(D_r^{[1]}, \ldots, D_r^{[h]}, \ldots, D_r^{[H]}\right), \; D_r^{[h]} = \left(n_{l_1,r}^{[h]}, n_{l_2,r}^{[h]}, \ldots, n_{l_M,r}^{[h]}\right) \tag{54}$$

Note that this does not include the genotyping data on the ancestral. Let $N^{[h]}$ be the sample size for the corresponding region in generation h:

$$N_r^{[h]} = \sum_l n_{l,r}^{[h]} \tag{55}$$

First, we obtain the maximum observed frequency of each RWH over the generations, denoted by q_l:

$$q_{l,r} = \max\left(\frac{n_{l,r}^{[1]}}{N_r^{[1]}}, \frac{n_{l,r}^{[2]}}{N_r^{[2]}}, \ldots, \frac{n_{l,r}^{[H]}}{N_r^{[H]}}\right) \tag{56}$$

Then, for each region the K_P RWHs having the highest values of $q_{l,r}$ will constitute the set of primary RWHs for that region. Afterwards, the primary lineages are formed by taking all possible combinations of the primary RWHs in the L regions. The secondary lineages, on the other hand, are obtained by sampling the RWHs for each region. If it happens that a secondary lineage is identical to a primary lineage, only one instance of that lineage is retained in the ancestral; and that lineage is considered to be a primary lineage. Therefore, the primary lineages are obtained under a setting corresponding to linkage equilibrium among the primary RWHs for the various regions, even though in our setting of exclusive selfing linkage disequilibrium is very high We took this approach, however, because it is more conservative and because it assures that the ancestral population will contain at least one of the lineages that are consistent with the most common RWHs over the regions that were defined (i.e., across chromosomes I and II, III and IV, and V and X). Note, as well, that new segregants/recombinants between RWH will be identified if they appear at a sufficient frequency during experimental evolution.

This concludes the description of the approach for sampling the ancestral given the genotyping data on a single replicate population. After running this step for all replicate populations, the overall ancestral population is obtained by taking the union of the lineages sampled for each replicate population.

1.11.2 Estimating the initial lineage frequencies

Once the ancestral population has been sampled, the initial lineages frequencies ($g_0^{[h]}$) are estimated based on the genotyping data on the ancestral population:

$$D_0 = \left(n_{l_1}^{[0]}, n_{l_2}^{[0]}, \ldots, n_{l_M}^{[0]}\right) \tag{57}$$

Given X_0 and A (equation 24), the initial lineage frequencies $g^{[0]}$ are obtained by maximizing the log-likelihood, similarly to equation 40:

$$\mathcal{L}_{g^{[0]}}\left(g^{[0]} \mid X_0, A\right) = \sum_l n_l^{[0]} \log\left(\sum_{k=1}^{G} \mathbb{I}\left(l, \mathcal{S}_k\right) g_k^{[0]}\right) \tag{58}$$

The gradient for the log-likelihood in equation 58 follows from equation 43. As for fitting the model, estimation was done using L-BFGS (Nocedal, 1980; Liu and Nocedal, 1989), via the NLopt package (version 2.4.2) in R (version 1.0.4; Johnson, 2017). We performed multiple runs of the optimization algorithm, each run starting from a random initial condition, and kept the estimate for $g^{[0]}$ with the largest likelihood value.

1.12 Adaptation during experimental evolution at different population sizes in constant high salt

1.12.1 Inferring the occurrence of an L28 sweep given the pooled genotyping

For the second set of evolution experiments, with different population sizes, we are interested in determining the probability of a sweep involving the L28 lineage given pooled genotyping data. Let $\mathcal{G}$ denote the set of lineages of interest, which are those lineages that differ from L28 by a value lower than a threshold number of SNPs. We require that $\mathcal{G}$ is non-empty, since at least lineage L28 belongs to it. For the analysis of the experimental data, we used a threshold value of 1, such that all lineages that are indistinguishable from L28, given the 29 SNPs that were assessed via pooled genotyping, are essentially considered as being L28.

Let q_i denote the allele frequency for SNP i, and let $\vec{q} = (q_1, q_2, \ldots, q_{N_S})$ denote the frequencies of N_S SNPs measured in a given population. We define the function $\chi(\cdot)$ to quantify the discrepancy relative to the alleles that define lineage L28:

$$\chi(\vec{q}) = \sqrt{\frac{1}{N_S} \sum_{i=1}^{N_S} (q_i - a_i)^2}, \; 0 \le \chi(\vec{q}) \le 1 \tag{59}$$

$$a_i = \begin{cases} 1, & \text{if L28 has the alternative allele in SNP } i \\ 0, & \text{if L28 has the reference allele in SNP } i \end{cases} \tag{60}$$

We let t denote the generation since the beginning of the second set of evolution experiments; $t = 0$ thus corresponds to generation 35 of the first evolution experiment. Let then $\mathbb{U}(x)$ be the step function:

$$\mathbb{U}(x) = \begin{cases} 1, & \text{if } x > 0 \\ 0, & \text{otherwise} \end{cases} \tag{61}$$

For this analysis, we are interested in the probability of a sweep taking place at a given generation $t > 0$, which is defined as:

$$p^{(t)}_{\text{sweep}} = \mathbb{P}\left[\sum_{k \in \mathcal{G}} \mathbb{U}\left(g^{(t)}_k - g^{(0)}_k\right) > 0 \;\middle|\; \chi^{(t)}_{\text{obs}} \right] \tag{62}$$

where $\chi^{(t)}_{\text{obs}} = \chi(\vec{q}_{\text{obs}})$ is obtained given the allele frequencies that were measured experimentally in generation t.

In order to estimate $p^{(t)}_{\text{sweep}}$ (equation 62), we simulate the dynamics of the lineages, under multiple scenarios. For each such simulation, we calculate the values of $\chi^{(t)}_{\text{sim}} = \chi(\vec{q}_{\text{sim}})$, based on the simulated allele frequencies. With the interval $[0, 1]$ divided into 50 bins, each with width 0.02, we estimate $p^{(t)}_{\text{sweep}}$ as the empirical frequency of simulations in which $\sum_{k \in \mathcal{G}} \mathbb{U}\left(g^{(t)}_k - g^{(0)}_k\right) > 0$ among those simulations in which $\chi^{(t)}_{\text{sim}}$ falls in the same bin as $\chi^{(t)}_{\text{obs}}$.

For multiple simulations, we sampled the parameters Θ of the fitness reaction norms, obtained for each sampled ancestral population (A). From these, we then

obtained the estimates for the fitness of the L lineages in the high salt environment:

$$\vec{\Psi}_H = \begin{bmatrix} \psi_1 & \psi_2 & \dots & \psi_k & \dots & \psi_G \end{bmatrix} \in \Re^G \tag{63}$$

$$\psi_k = \log\left(\lambda_k(x)\right)\big|_{x=1} \tag{64}$$

To start the simulations, the initial frequencies of the lineages also have to be defined. For this, we relied on estimates of the lineage frequencies produced by the model (that was fit to the data on the first experiment) in generation 35 under the gradual regime.

For each simulation, we sampled the lineage fitness values Ψ_k via a multivariate normal distribution centered on the values that were initially estimated:

$$\tilde{\vec{\Psi}}_H \sim \mathcal{N}\left(\Psi_H, 3\,\sigma_\Psi^2\, I_G\right) \tag{65}$$

where $I_G \in \Re^{G,G}$ is the identity matrix, and $\sigma_\Psi^2 \in (0, \infty)$ is given by:

$$\sigma_\Psi^2 = \frac{1}{G-1} \sum_{k=1}^{G} \left(\psi_k - \bar{\psi}\right)^2, \; \bar{\psi} = \frac{1}{G} \sum_{k=1}^{G} \left(\psi_k\right)^2 \tag{66}$$

1.12.2 Principal component analysis

The function `prcomp` in R was used for principal component analysis, applied on the matrix containing the frequency of the alternative allele for each SNP in each sample (population assayed).

2 References for supplementary information

References

Bates, D., Mächler, M., Bolker, B., and Walker, S. (2015). Fitting Linear Mixed-Effects Models Using lme4. *Journal of Statistical Software*, 67(1).

Bradić, M., Costa, J., and Chelo, I. M. (2011). Genotyping with Sequenom. In Orgogozo, V. and Rockman, M. V., editors, *Molecular Methods for Evolutionary Genetics*, pages 193–210. Humana Press.

Bürger, R. (2000). *The Mathematical Theory of Selection, Recombination, and Mutation.* Wiley.

Chelo, I. M. and Teotónio, H. (2013). The opportunity for balancing selection in experimental populations of Caenorhabditis elegans. *Evolution*, 67(1):142–156.

Crow, J. F. and Kimura, M. (1970). *An Introduction to Population Genetics Theory.* Harper and Row, New York.

Dey, S., Proulx, S. R., and Teotonio, H. (2016). Adaptation to Temporally Fluctuating Environments by the Evolution of Maternal Effects. *PLoS Biology*, 14(2):1–29.

Elzhov, T. V., Mullen, K. M., Spiess, A.-N., and Bolker, B. (2016). *minpack.lm: R Interface to the Levenberg-Marquardt Nonlinear Least-Squares Algorithm Found in MINPACK, Plus Support for Bounds.*

Gresham, D., Boer, V. M., Caudy, A., Ziv, N., Brandt, N. J., Storey, J. D., and Botstein, D. (2011). System-level analysis of genes and functions affecting survival during nutrient starvation in Saccharomyces cerevisiae. *Genetics*, 187(1):299–317.

Illingworth, C. J. R., Fischer, A., and Mustonen, V. (2014). Identifying Selection in the Within-Host Evolution of Influenza Using Viral Sequence Data. *PLoS Computational Biology*, 10(7).

Johnson, S. G. (2017). The NLopt nonlinear-optimization package. http://ab-initio.mit.edu/nlopt.

Le Hellard, S., Ballereau, S. J., Visscher, P. M., Torrance, H. S., Pinson, J., Morris, S. W., Thomson, M. L., Semple, C. a. M., Muir, W. J., Blackwood, D. H. R., Porteous, D. J., and Evans, K. L. (2002). SNP genotyping on pooled DNAs: comparison of genotyping technologies and a semi automated method for data storage and analysis. *Nucleic acids research*, 30(15):e74.

Lenth, R. V. (2016). Least-Squares Means: The {R} Package {lsmeans}. *Journal of Statistical Software*, 69(1):1–33.

Liu, D. C. and Nocedal, J. (1989). On the limited memory BFGS method for large scale optimization. *Mathematical Programming*, 45(1-3):503–528.

Matuszewski, S., Hermisson, J., and Kopp, M. (2015). Catch Me if You Can: Adaptation from Standing Genetic Variation to a Moving Phenotypic Optimum. *Genetics*, 200(4):1255–1274.

Maupas, E. (1900). Modes et formes de reproduction des nematodes (Translated from French to English by M.-A. Felix). *Arch. Exp. Gen. Ser.*, 3:463– 624.

Meyer, B. J. (2005). X-Chromosome dosage compensation. *WormBook*, pages 1–14.

Neher, R. A. (2013). Genetic Draft, Selective Interference, and Population Genetics of Rapid Adaptation. *Annual Review of Ecology, Evolution, and Systematics*, 44(1):195–215.

Noble, L., Chelo, I., Guzella, T., Afonso, B., Riccardi, D., Ammerman, P., Dayarian, A., Carvalho, S., Crist, A., Pino-Querido, A., Shraiman, B., Rockman, M., and Teotonio, H. (2017). Polygenicity and epistasis underlie fitness-proximal traits in the Caenorhabditis elegans multiparental experimental evolution (CeMEE) panel. *Preprint at bioRxiv, doi:10.1101/120865*.

Nocedal, J. (1980). Updating Quasi-Newton Matrices with Limited Storage. *Mathematics of Computation*, 35(151):773.

R Core Team (2016). *R: A Language and Environment for Statistical Computing*. R Foundation for Statistical Computing, Vienna, Austria.

Rockman, M. V. and Kruglyak, L. (2009). Recombinational landscape and population genomics of Caenorhabditis elegans. *PLoS Genetics*, 5(3):e1000419.

Scheet, P. and Stephens, M. (2006). A fast and flexible statistical model for large-scale population genotype data: applications to inferring missing genotypes and haplotypic phase. *American Journal of Human Genetics*, 78(4):629–44.

Schiffels, S., Szollosi, G. J., Mustonen, V., and Lässig, M. (2011). Emergent neutrality in adaptive asexual evolution. *Genetics*, 189(4):1361–1375.

Teotonio, H., Carvalho, S., Manoel, D., Roque, M., and Chelo, I. M. (2012). Evolution of outcrossing in experimental populations of Caenorhabditis elegans. *PloS one*, 7(4):e35811.

Teotonio, H., Estes, S., Phillips, P. C., and Baer, C. F. (2017). Experimental Evolution with Caenorhabditis Nematodes. *Genetics*, 206(2):691–716.

Theologidis, I., Chelo, I. M., Goy, C., and Teotónio, H. (2014). Reproductive assurance drives transitions to self-fertilization in experimental Caenorhabditis elegans. *BMC Biology*, 12(1):93.
