# Supplementary figures and images for: Slower environmental change hinders adaptation from standing genetic variation
Source: PLoS Genet. 2018 Nov 1;14(11):e1007731. doi: 10.1371/journal.pgen.1007731 (PMC6233921; doi:10.1371/journal.pgen.1007731)

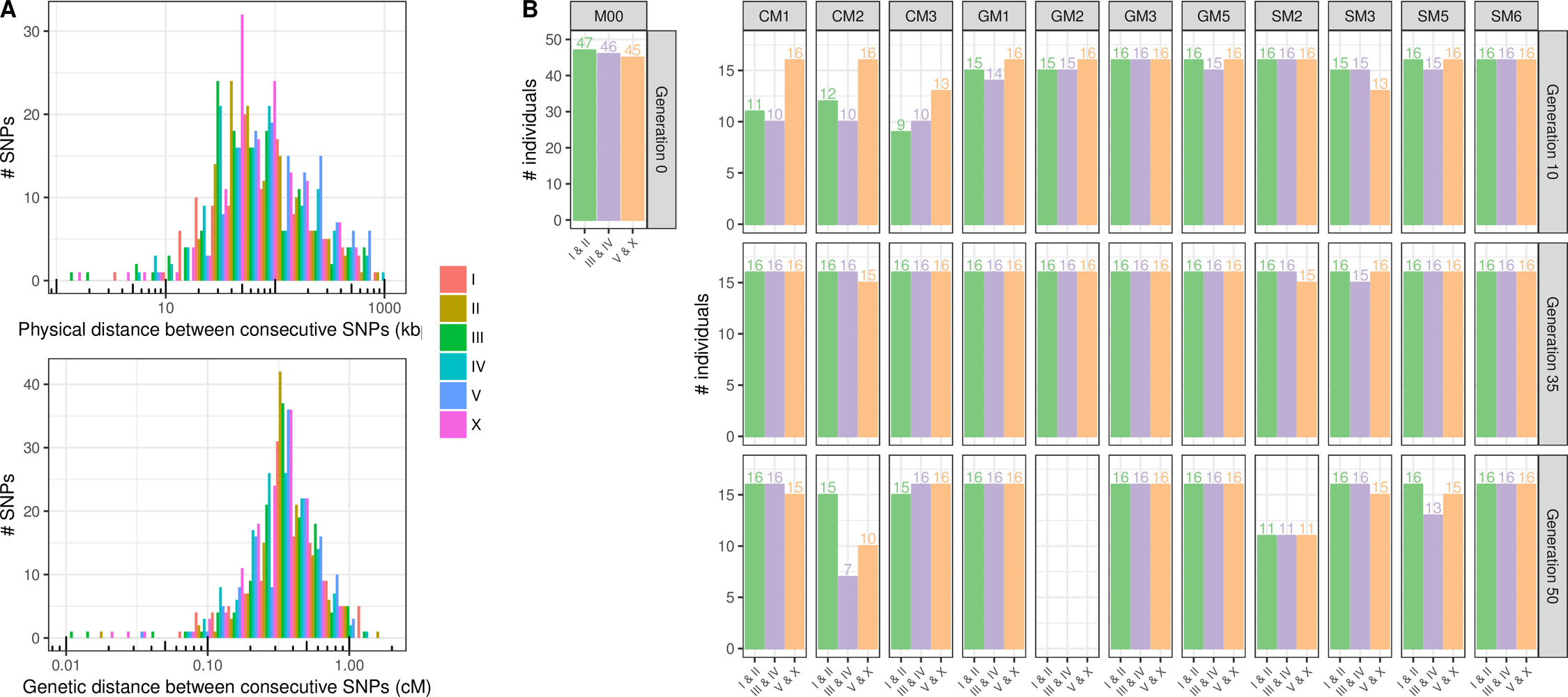

Supplement: S1 Fig — (A) Histograms showing the distance between consecutive SNPs in each of the six chromosomes after quality control. Top figure shows physical distance (in kbp, see [53] for gene sizes and densities in C. elegans), and bottom figure shows genetic distances (in cM, based on C. elegans genetic map of [24], each chromosome with 50cM). (B) Number of individuals for which data is available within the ancestral population (M00) and within each evolved replicate population in the respective time-points; note that gradual population GM2 was not genotyped at generation 50. The numbers shown correspond to those obtained after quality control of the data. For the ancestral population, the sample size is around 3 times larger that of the evolved replicate populations. (PNG) [file pgen.1007731.s002.png]

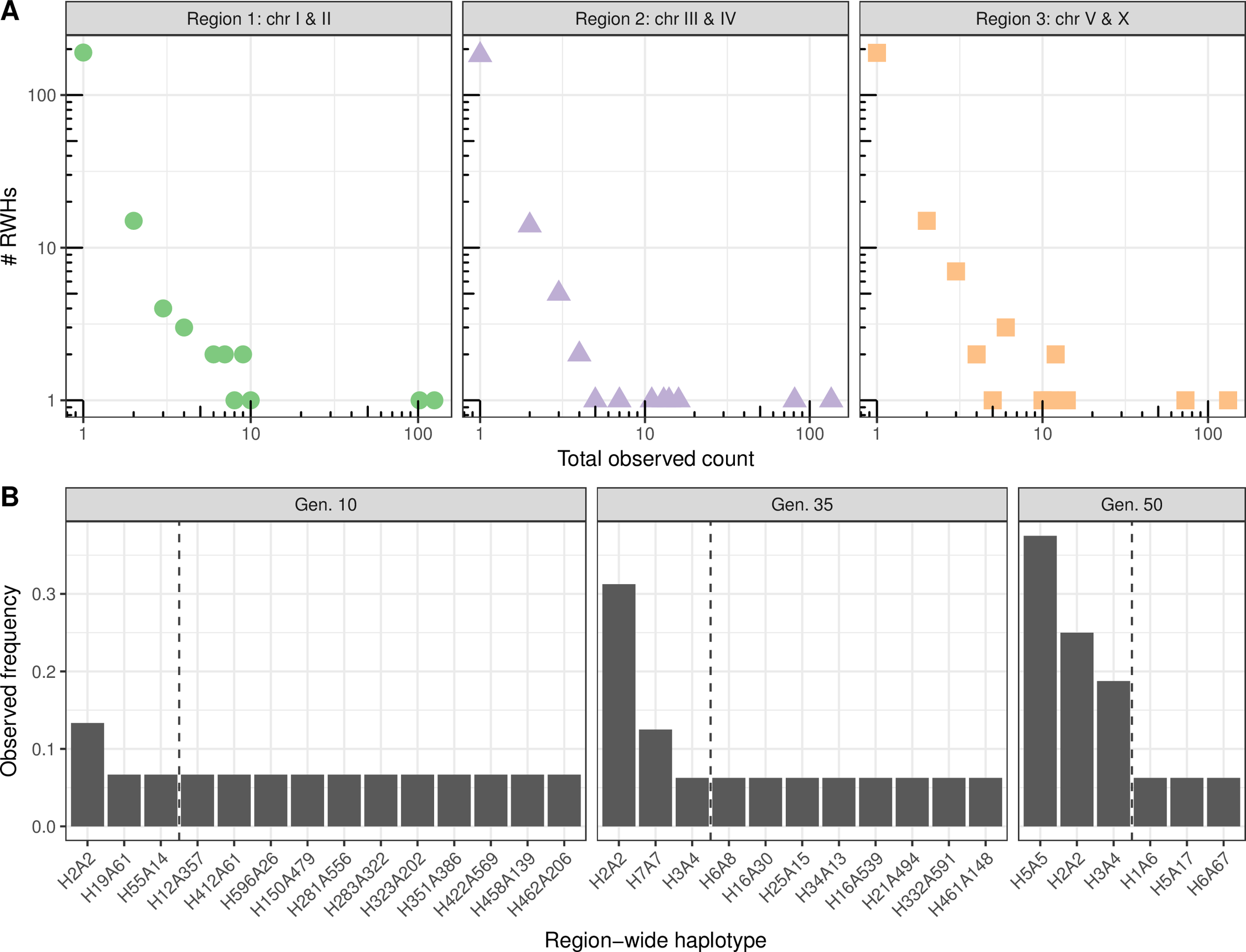

Supplement: S2 Fig — (A) Most of the RWHs observed across all time points and population sampled are relative rare. Each panel shows the histogram with number of RWH relative to all those observed for each sub-genomic region (left to right). (B) Outline of the approach for defining the major and minor RWHs in a given replicate population; data shown for the gradual population GM1). To the left of the vertical dashed line, all those RWH that were grouped into a single “background” RWH (named H0A, H0B or H0C). (PNG) [file pgen.1007731.s003.png]

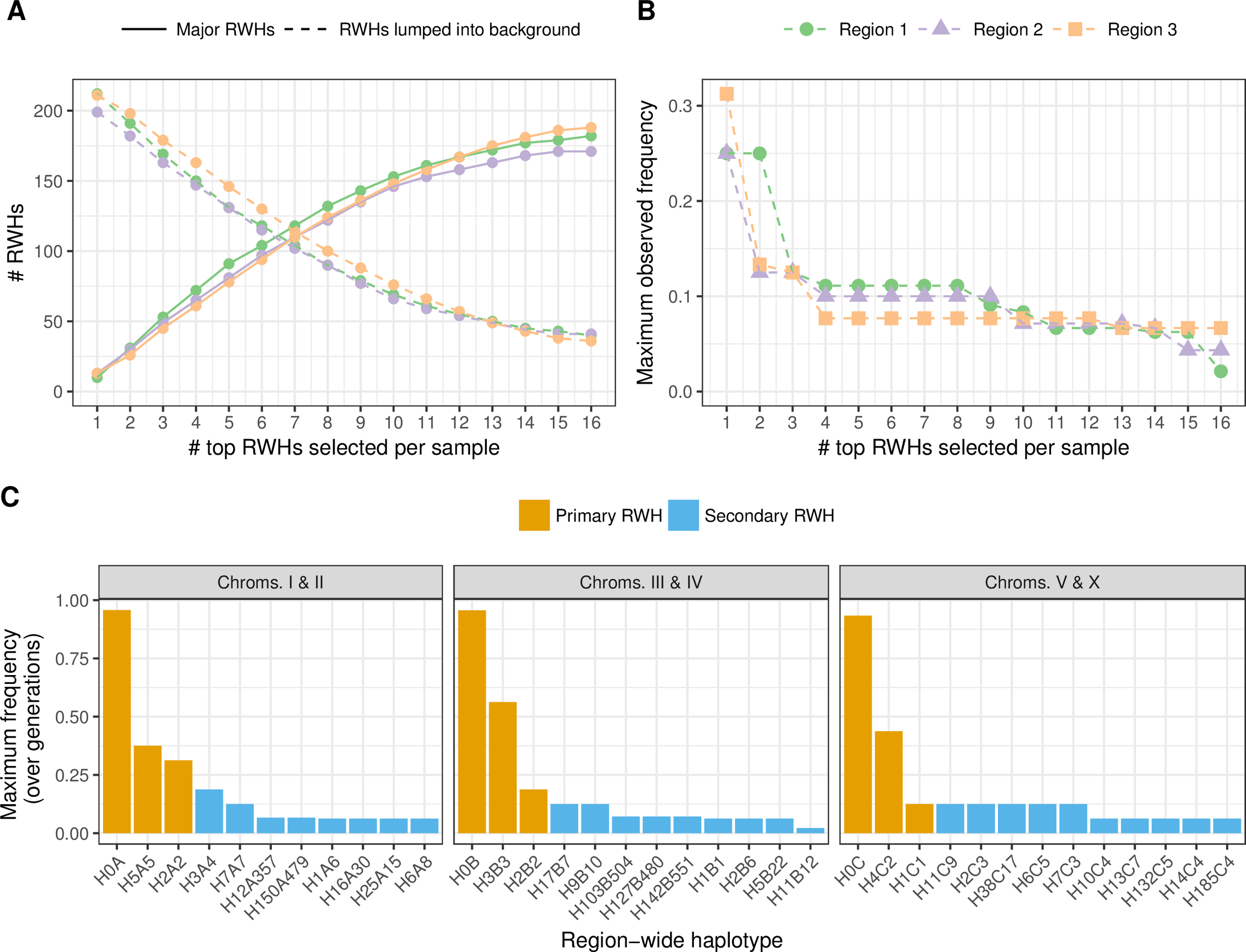

Supplement: S3 Fig — (A) Shown is the number of RWHs classified as major and minor as a function of the number of top RWHs selected per sample. (B) Shown is the maximum observed frequency for the minor RWHs as a function of number of top RWHs selected per sample. For the selected value of 3 top RWHs per sample, the maximum frequency is around 10%. (C) For each replicate population, the RWHs are ranked based on the maximum observed frequency across the generations, with the ones attaining the highest maximum frequencies within each region being defined as primary RWHs. Results are shown for replicate population GM1, with the top 3 RWHs being defined as primary RWHs; H0A, H0B and H0C correspond to the background RWHs in the respective regions. (PNG) [file pgen.1007731.s004.png]

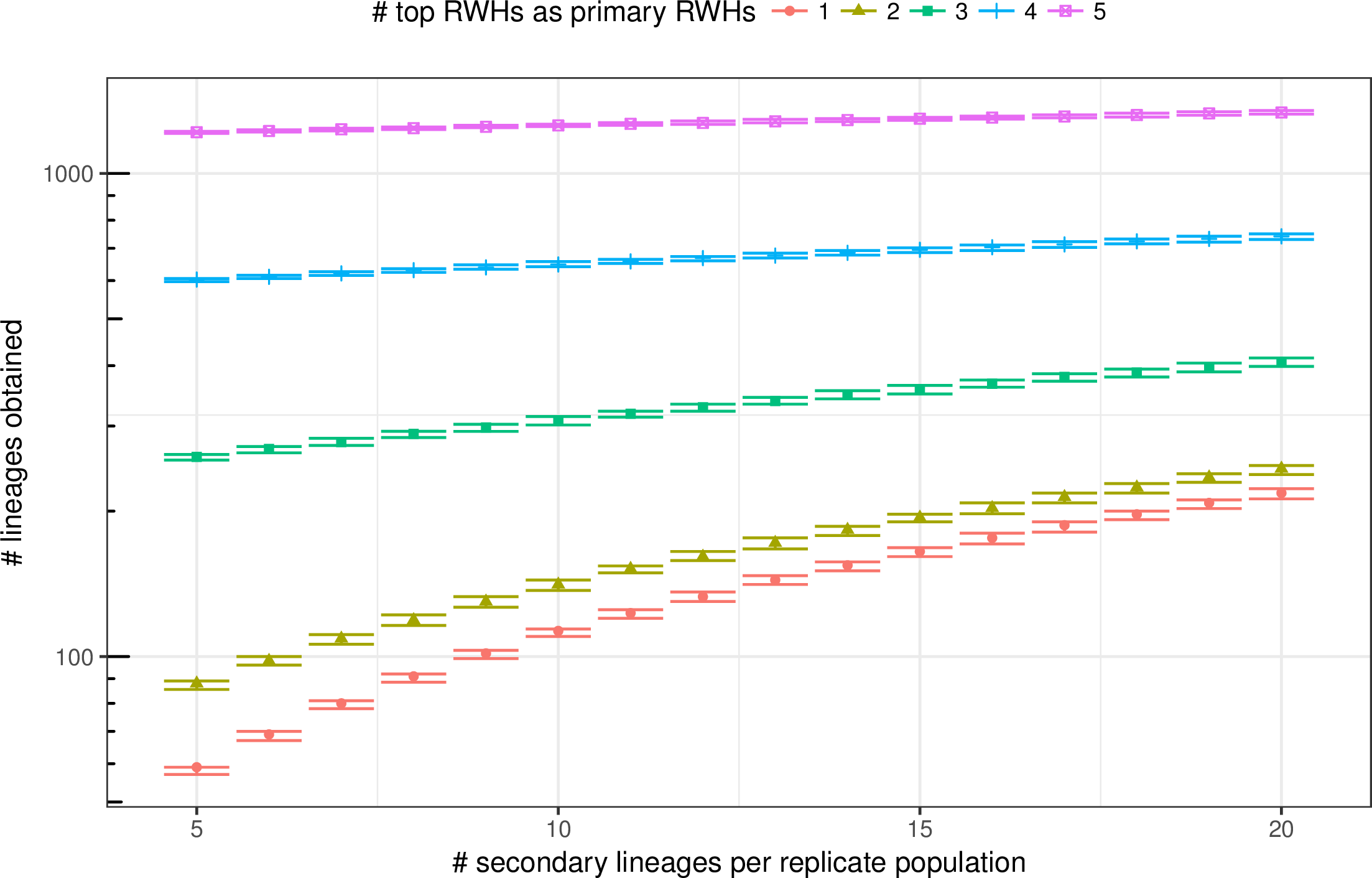

Supplement: S4 Fig — Number of lineages obtained as a function of the number of top RWHs selected as primary RWHs within each replicate population, and the number of secondary lineages sampled per replicate population. (PNG) [file pgen.1007731.s005.png]

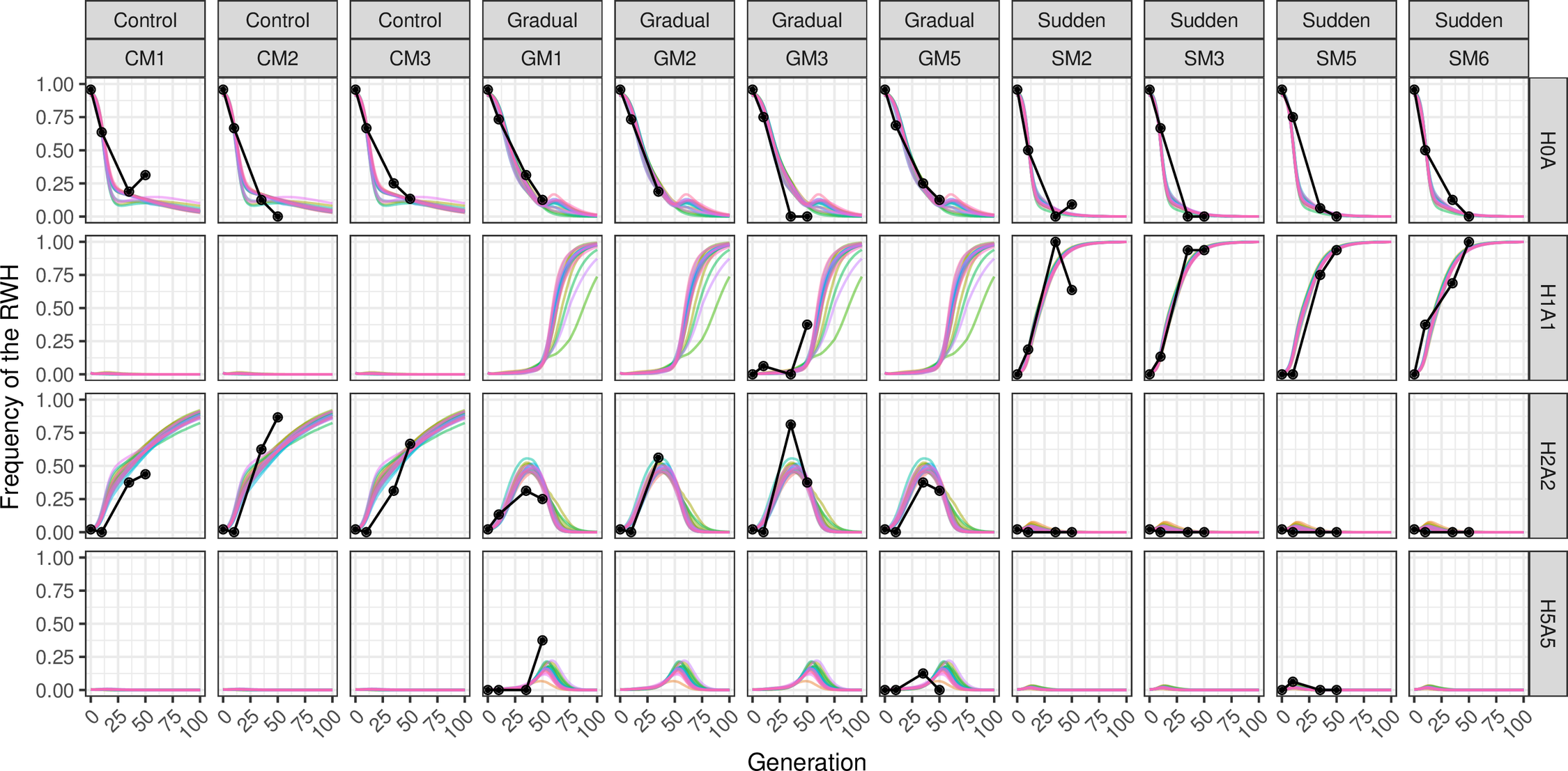

Supplement: S5 Fig — The observed (in part from Fig 3) and predicted frequencies of selected RWHs are shown as a function of the number of generations, in each of the replicate populations. RWHs are shown for region 1 (chromosomes I and II). Predicted frequencies when modeling linear frequency reaction norms are shown for 100 generations (assuming that the gradual populations would be kept at high salt after generation 35). Colored lines correspond to the predicted frequencies for each of the 20 sampled ancestral populations considered for inference. For simplicity, the figures show only the RWHs that are among the 2 RWHs having the largest frequencies per replicate population, considering the observed or predicted frequencies. Each RWH is shown in a single row of a given figure, while each column corresponds to a replicate population. Note that, if a given RWH has not been observed in a replicate population, only the predicted frequencies are shown. (PNG) [file pgen.1007731.s006.png]

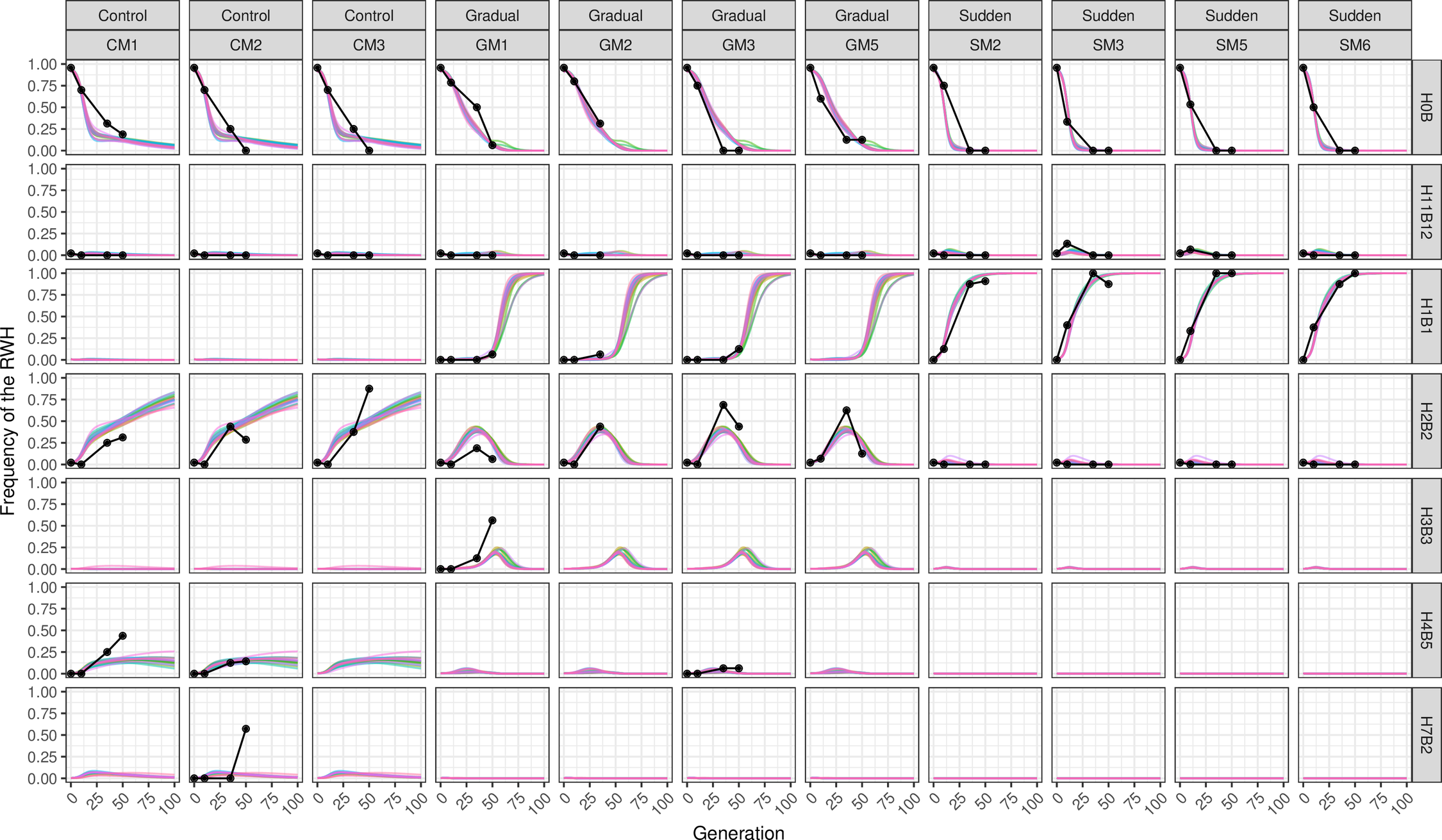

Supplement: S6 Fig — As in S5 Fig, but for RWHs in region 2 (chromosomes III and IV). (PNG) [file pgen.1007731.s007.png]

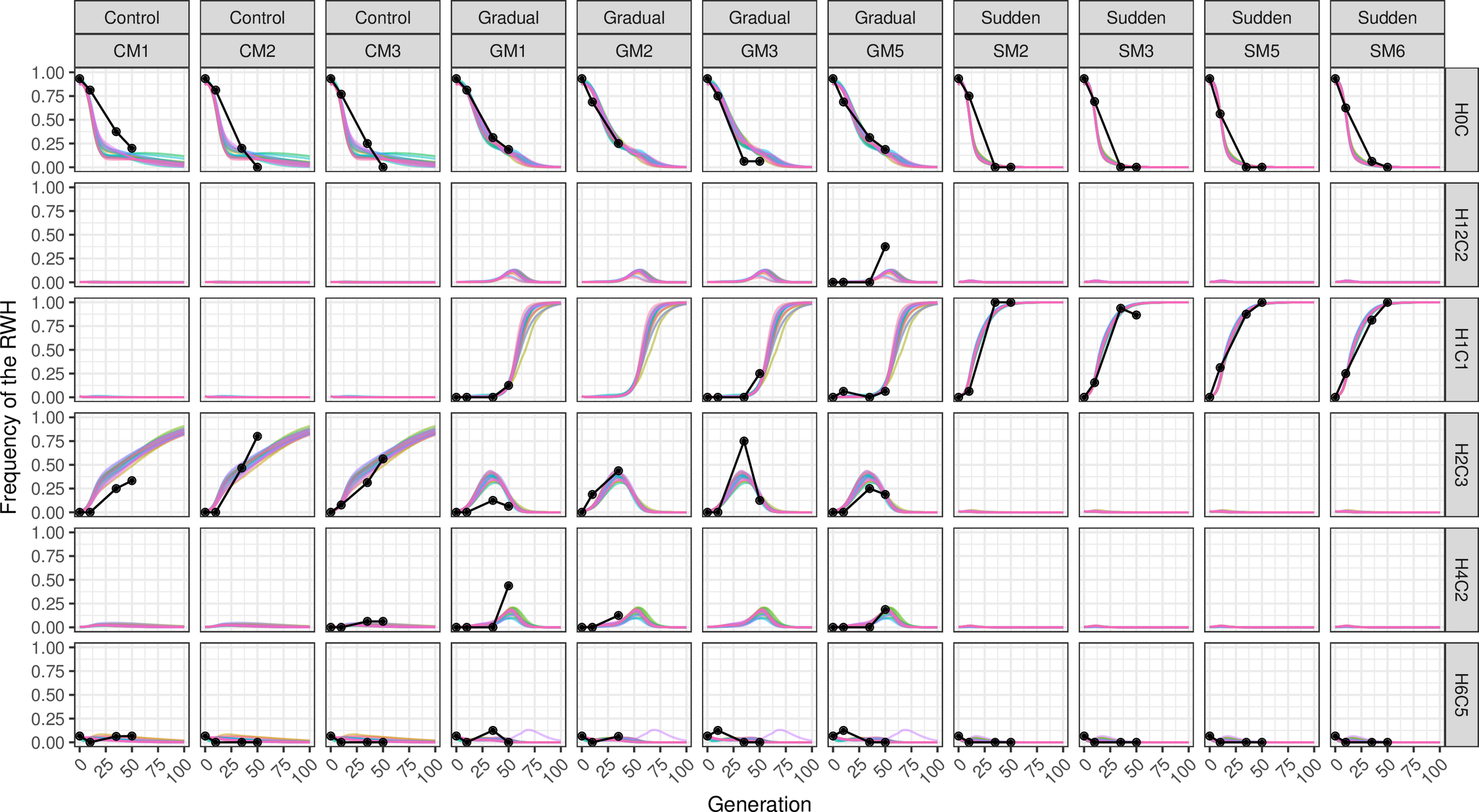

Supplement: S7 Fig — As in S5 Fig, but for RWHs in region 3 (chromosomes V and X). (PNG) [file pgen.1007731.s008.png]

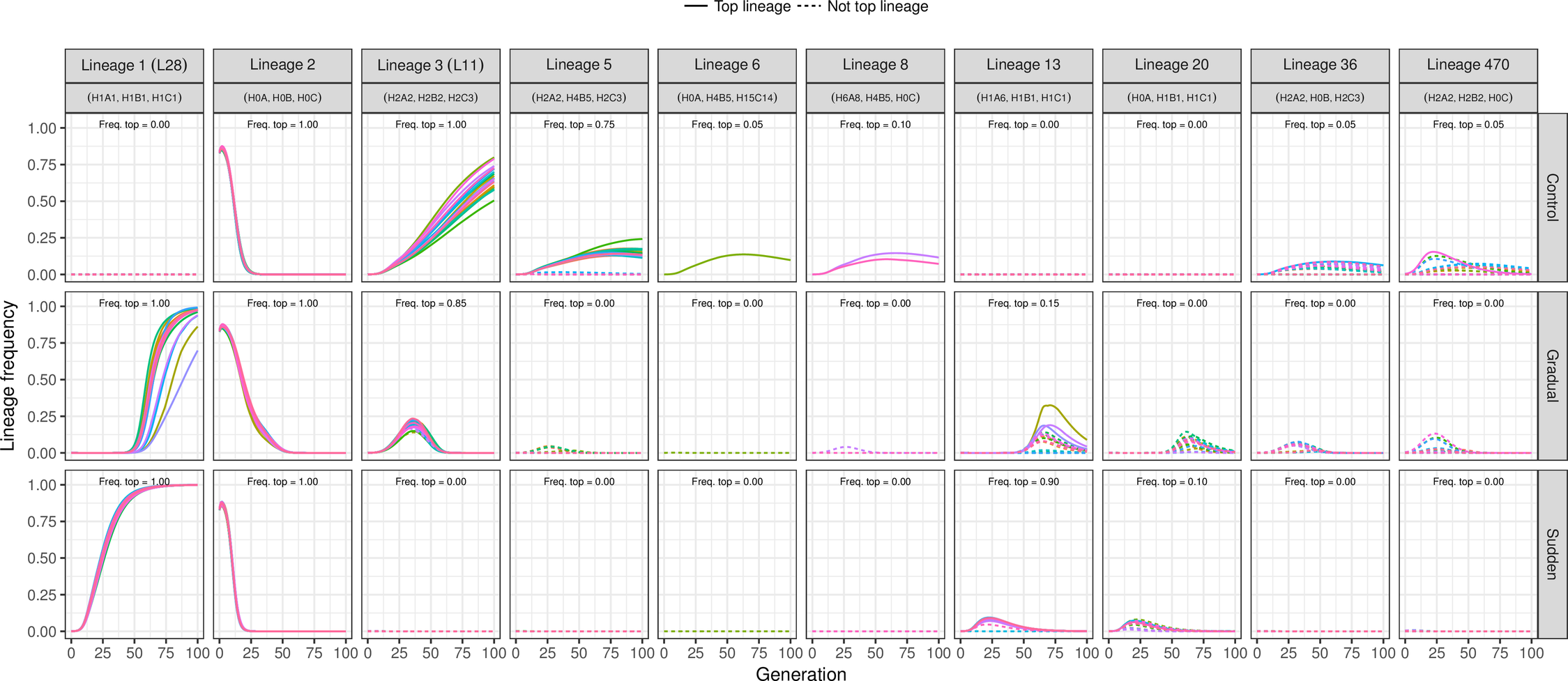

Supplement: S8 Fig — Shown are the predicted frequencies of the main lineages from generation 0 to generation 100 in each of the regimes (as in Fig 5B). Each colored line corresponds to the predicted frequencies for a given sampled ancestral population. For each sampled ancestral population, the lineages within each treatment were ranked based on the maximum frequency observed across the 100 generations. The top 3 lineages per case were then considered, and the frequency of ancestral populations in which each lineage appeared among the top 3 lineages was computed. Note that lineages 1 and 3 correspond, respectively, to L28 and L11. Each main lineage is shown in a single column, while each row corresponds to an experimental evolution regime (assuming gradual population would be kept in high salt conditions from generation 35 onwards). (PNG) [file pgen.1007731.s009.png]

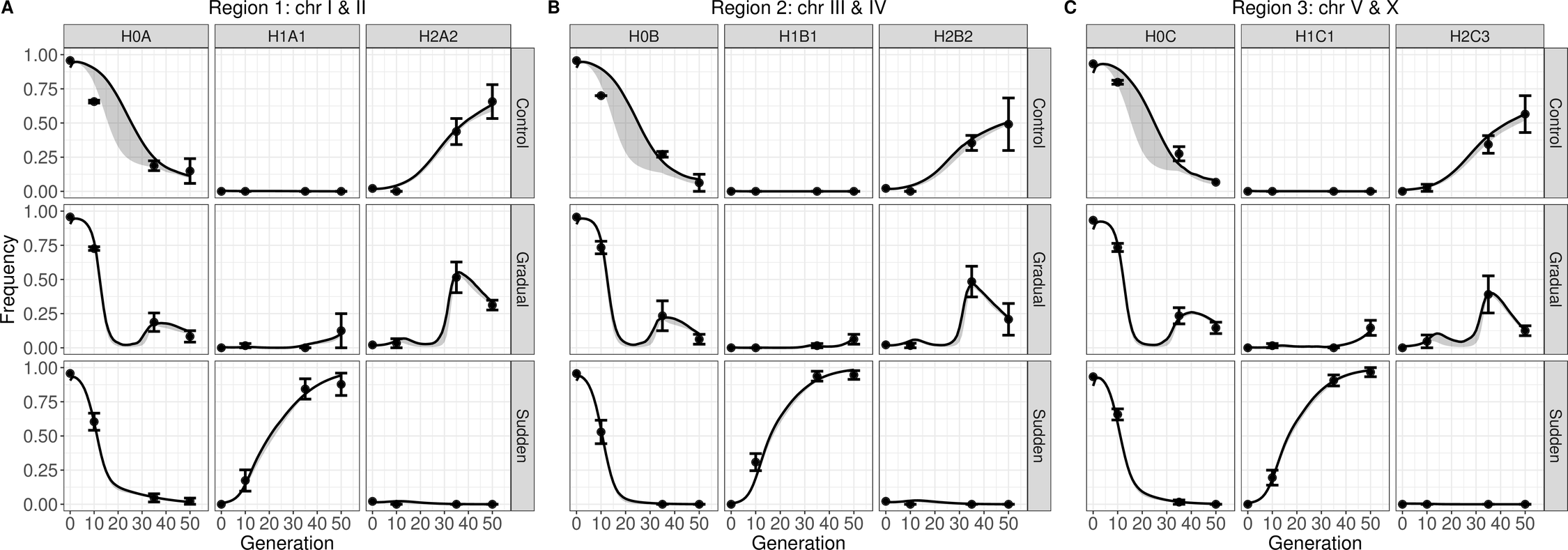

Supplement: S9 Fig — (A-C) Left columns show that the majority of haplotypes are quickly selected against in all experimental evolution regimes (as in Fig 3, background haplotypes H0#)). Middle and right columns show the two specific haplotypes in region 1 (A), region 2 (B) or region 3 (C) showing the greatest frequency change until generation 50. Dots and error bars are the mean and one standard error of the observed haplotype frequencies among replicate populations (same as in Fig 3). Lines show the inferred trajectories when RWH fitness reaction norms are modeled as quadratic, instead of linear as in Fig 3. Shaded grey area corresponds to the trajectories obtained by sampling the ancestral 20 times. (PNG) [file pgen.1007731.s010.png]

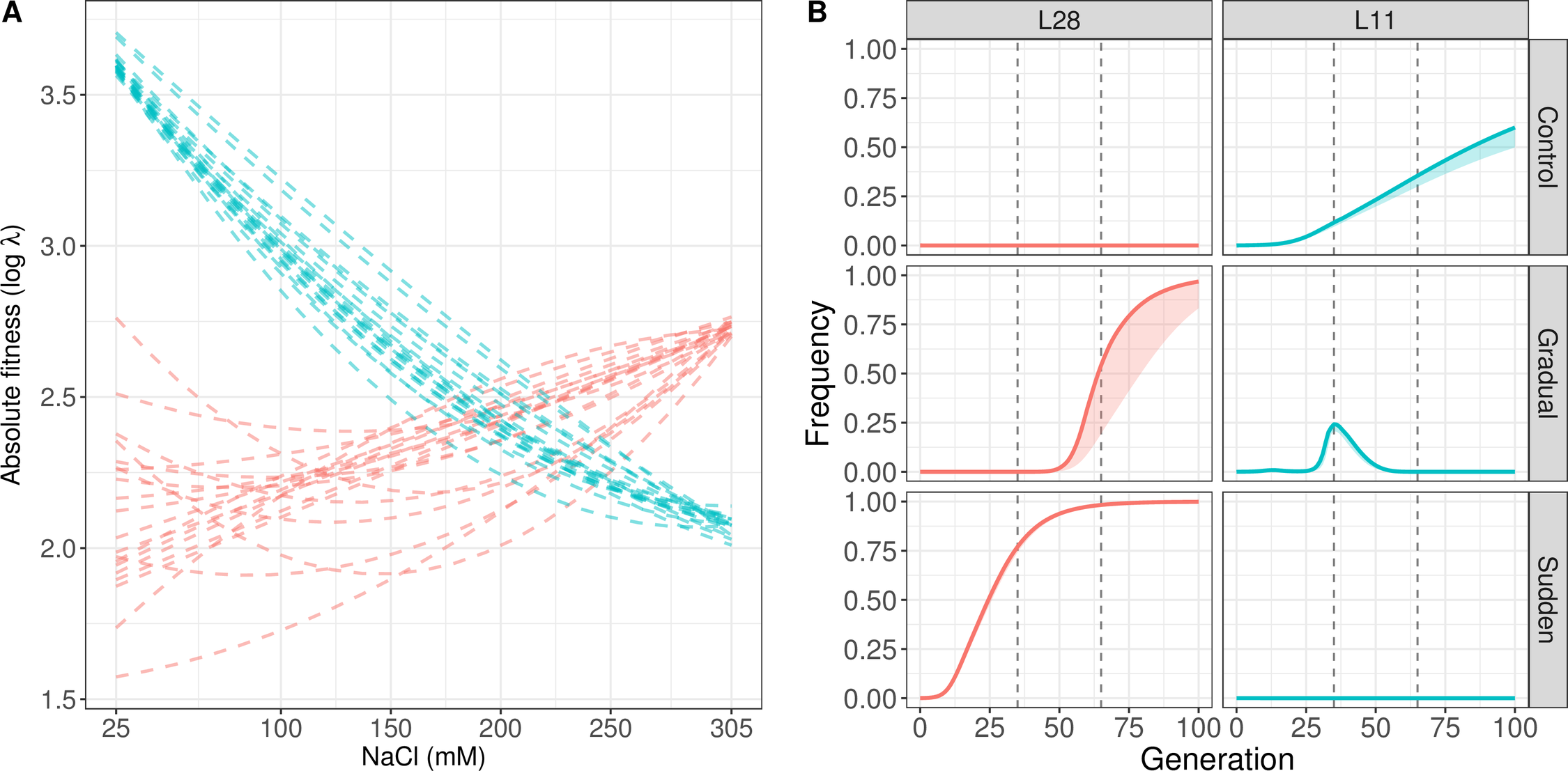

Supplement: S10 Fig — (A) Predicted fitness reaction norms of the L28 (red) and L11 (blue), as in Fig 5A, but modeling quadratic functions. Each line corresponds to one sample done of the ancestral population. (B) Expected L28 and L11 frequency trajectories for 100 generations (with the gradual populations being kept at high salt after generation 35). Shaded colors show the deterministic expectations based on simulating quadratic fitness reaction norms and sampling the ancestor population 20 times, with the line showing the median. (PNG) [file pgen.1007731.s011.png]

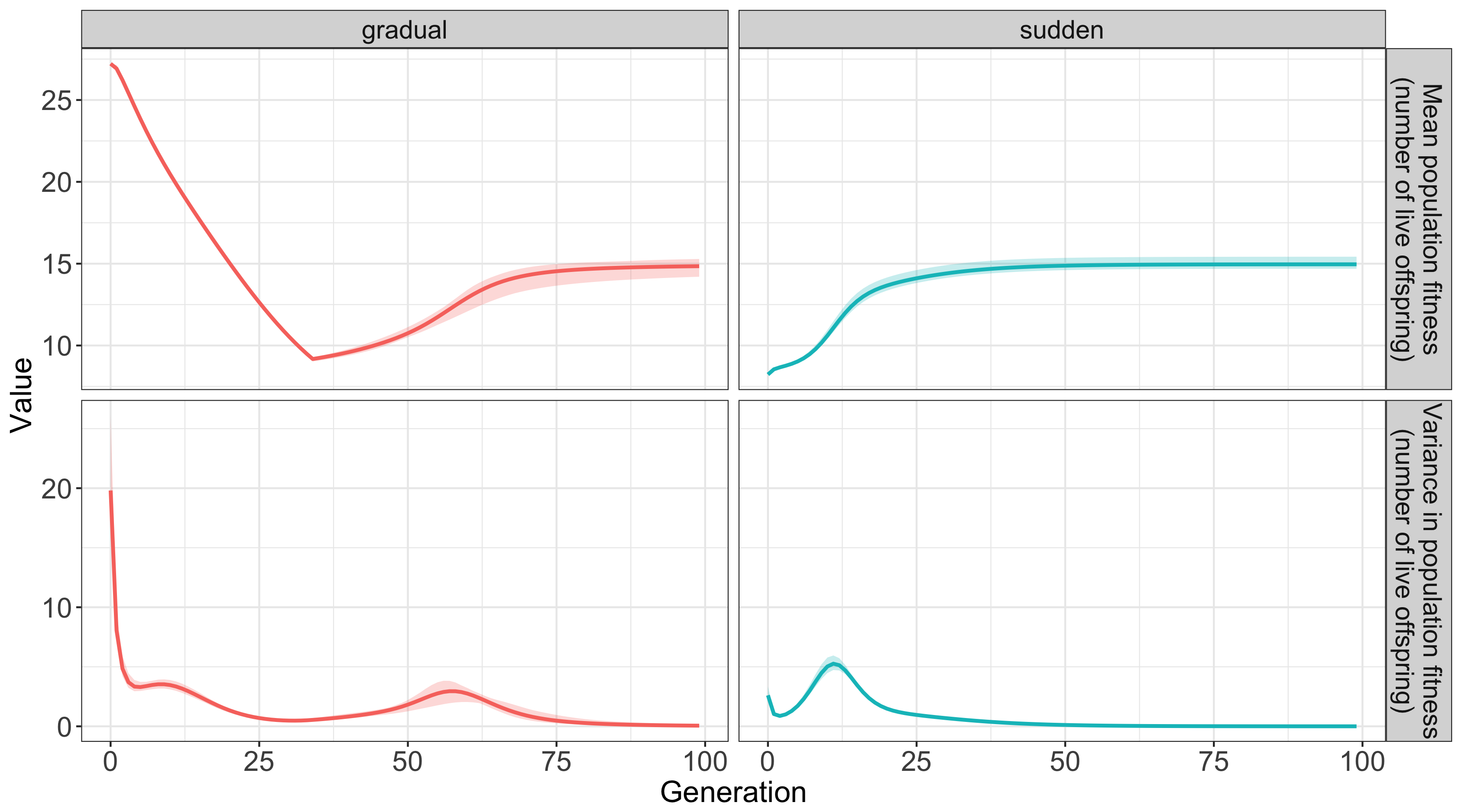

Supplement: S11 Fig — The average population fitness and variance in fitness for 20 samples of the ancestral population, under the sudden or the gradual regimes, when modeling linear reaction norms for each segregating lineage. Lines indicate the mean with shaded areas the 95% credible interval. (PNG) [file pgen.1007731.s012.png]

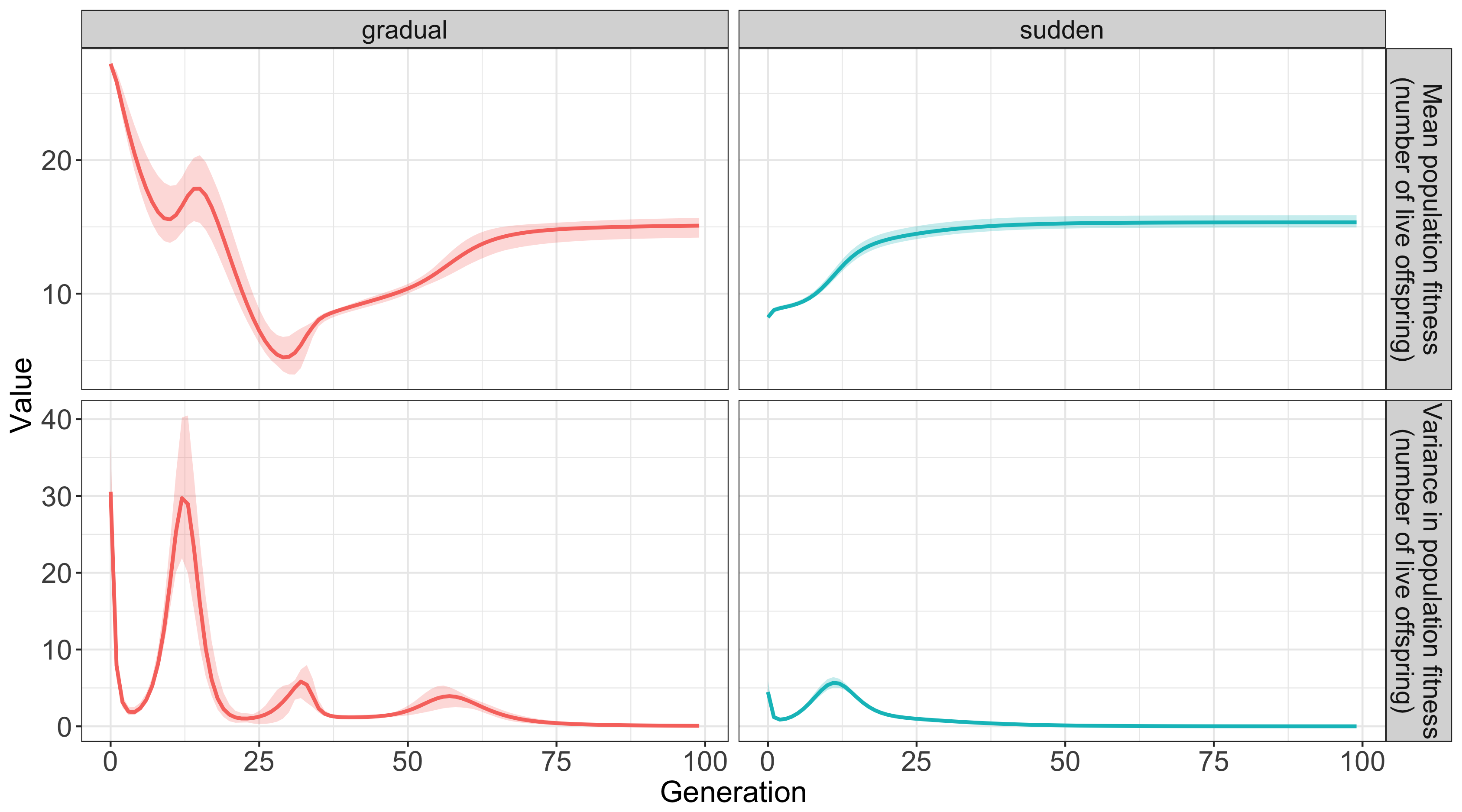

Supplement: S12 Fig — The average population fitness and variance in fitness for 20 samples of the ancestral population, under the sudden or the gradual regimes, when modeling quadratic reaction norms for each segregating lineage. Lines indicate the mean with shaded areas the 95% credible interval. (PNG) [file pgen.1007731.s013.png]

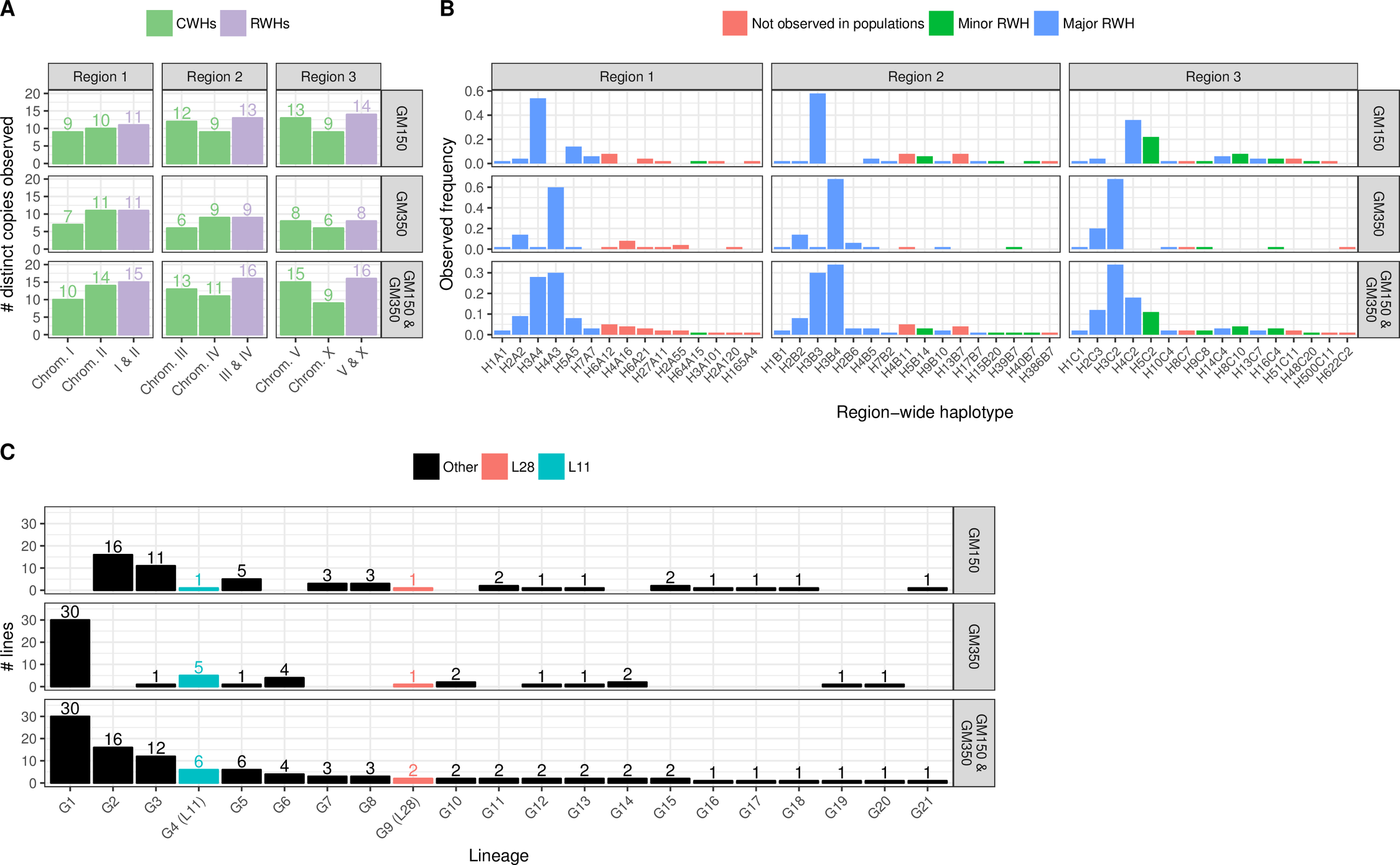

Supplement: S13 Fig — (A) 100 lines were derived from gradual populations (GM1 and GM3) at generation 50 and whole-genome sequenced in [21]. Most of the CWHs and RWHs observed in the present study are observed as well in the lines of [21]. Shown are the number of distinct CWHs and RWHs observed within each region, considering the lines derived from each replicate population (rows 1 and 2), and considering all lines (last row). (B) RWHs detected in high frequencies in the lines were also observed in the populations, having been assigned most often as major RWHs. (C) Overview of the lineages corresponding to the lines that were derived out of replicate populations GM1 and GM3 in generation 50. Shown are the number of lines corresponding to each lineage, considering each replicate population (rows 1 and 2), and both altogether (last row). The lineages are labelled based on their rank in terms of the frequency within the replicate populations. Lineages G4 and G9 correspond to L11 and L28 in [21] (see also S2 Table), respectively, and were observed in lines derived from both replicate populations. (PNG) [file pgen.1007731.s014.png]

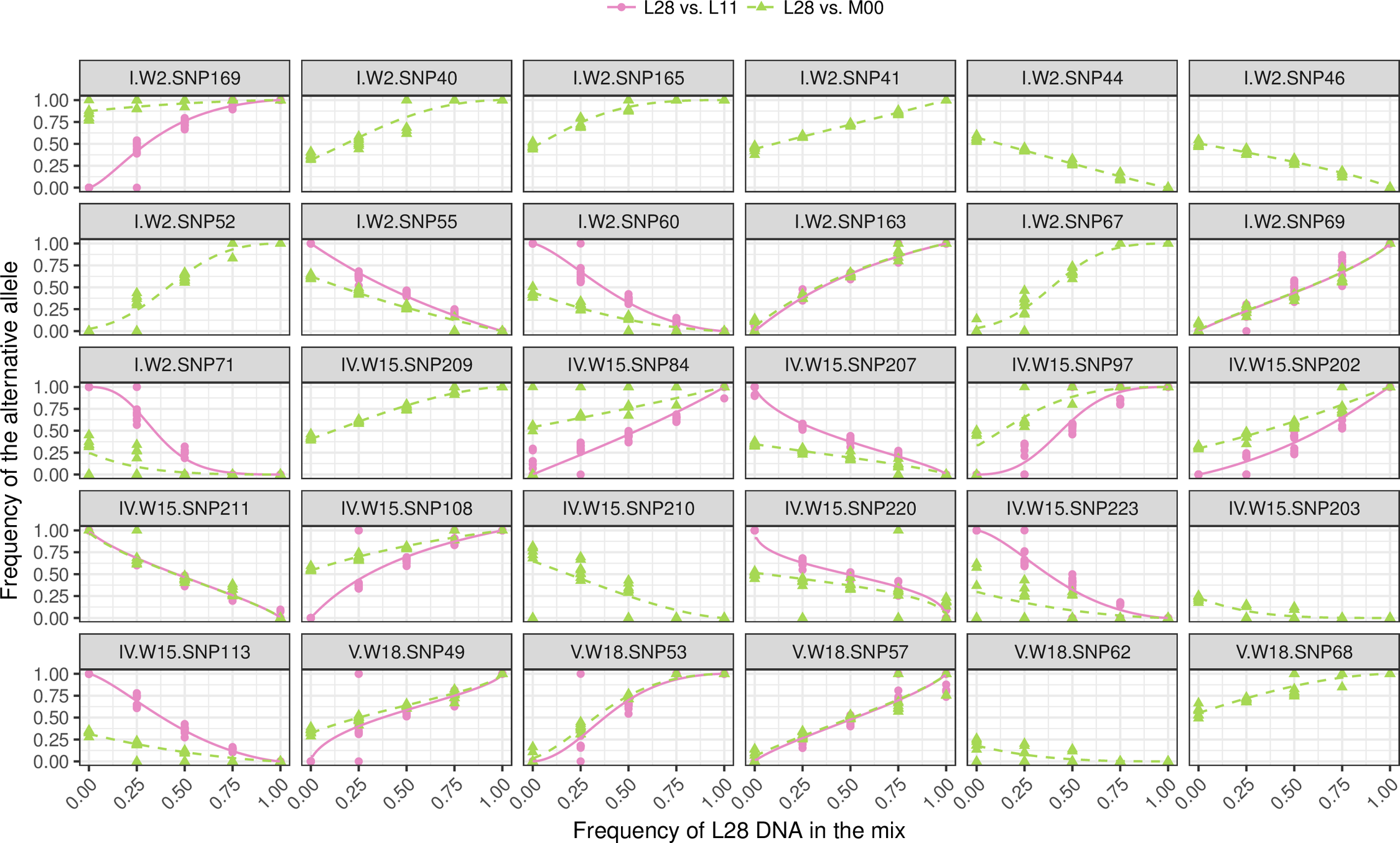

Supplement: S14 Fig — Pooled sequencing data was used to estimate lineage frequencies in the head-to-head competition assays (Fig 6D) and in the second set of evolution experiments (Fig 7). Each entry corresponds to a single SNP analyzed, with the symbols corresponding to the observed values, while the curves correspond to the fitted calibration curves over known ratio of L28 gDNA molarity versus L11 gDNA molarity (magenta) or known ratio of L28 gDNA molarity versus the ancestral population (M00) gDNA molarity (green). The former data has been omitted in the case that L28 and L11 have the same allele for the respective SNP. (PNG) [file pgen.1007731.s015.png]

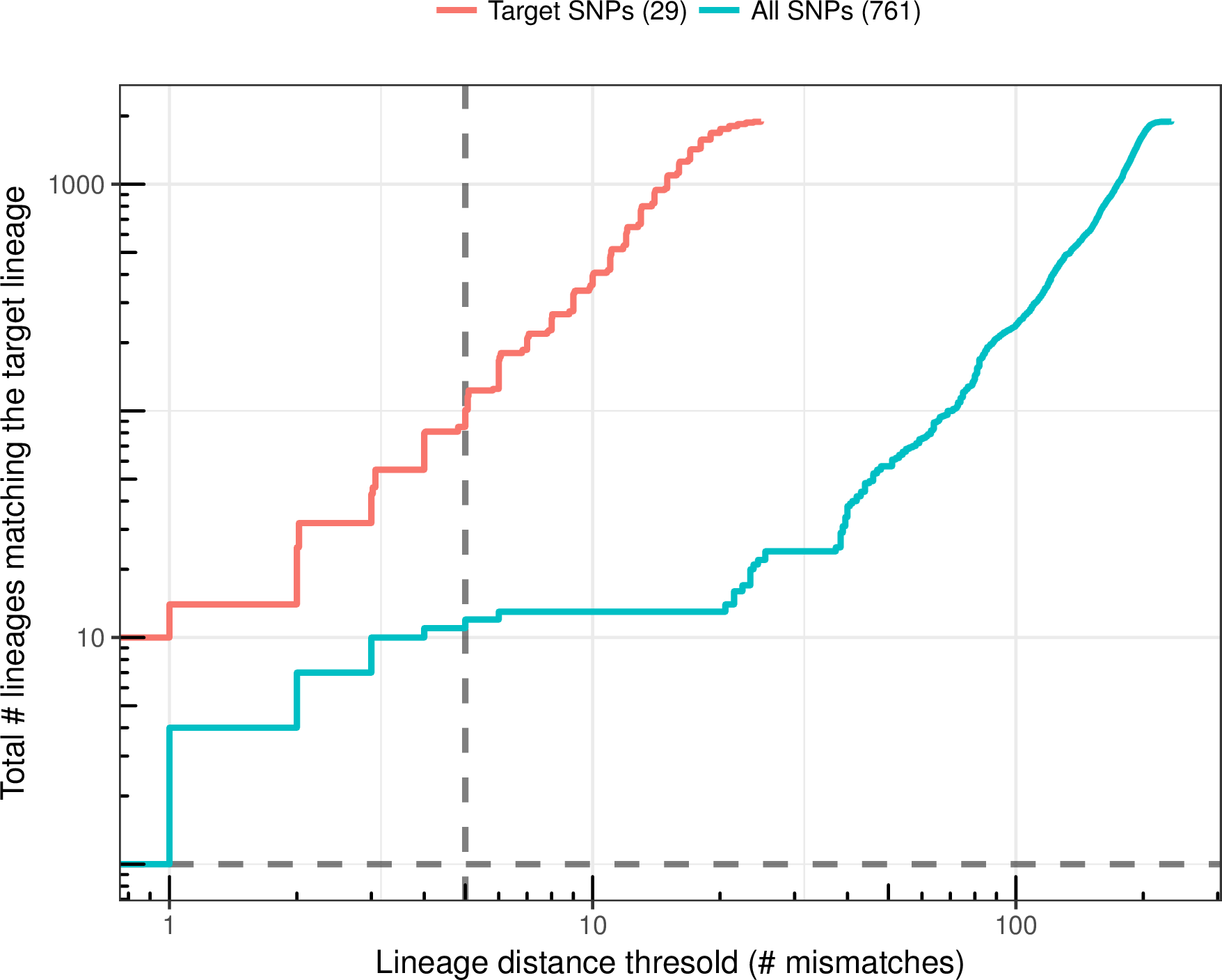

Supplement: S15 Fig — Impact of a subset of SNPs in terms of the ability to distinguish all possible segregating lineages (S4 Fig), contrasting the effect of considering the 29 target SNPs that were assayed in the pooled genotyping data (Fig 6D and Fig 7) with the full set of 761 SNPs used in the first set of evolution experiments. Shown is the number of lineages confounded with the target lineage (L28) as a function of the number of different SNPs (compared with L28). The figure is shown in a logarithmic scale in both axes. The number of lineages matching a threshold value lower than 1 corresponds to the lowest number of confounded lineages: 1 lineage, for all 761 SNPs (L28), and 10 lineages for the 29 target SNPs (L28 and 9 other lineages). (PNG) [file pgen.1007731.s016.png]

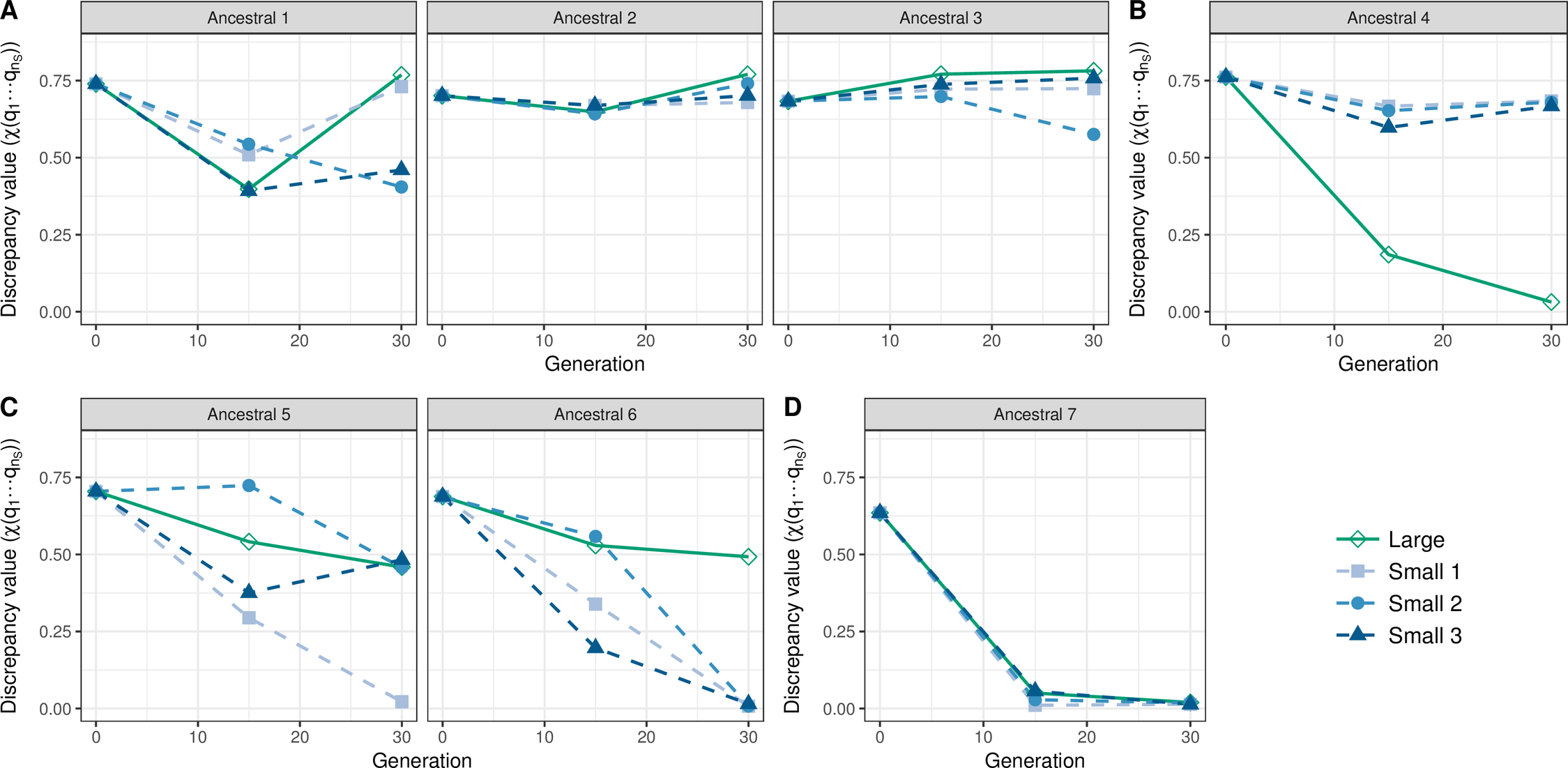

Supplement: S16 Fig — Shown are the discrepancy values for each ancestral population used to seed the continued experimental evolution under each of the experimental evolution regimes (large or small population size); (A) for populations where L28 was lost before continued experimental evolution, (B) for the population where L28 swept in large populations, (C) for the populations where L28 swept in some of the small populations and (D) where L28 swept regardless of population size. (PNG) [file pgen.1007731.s017.png]

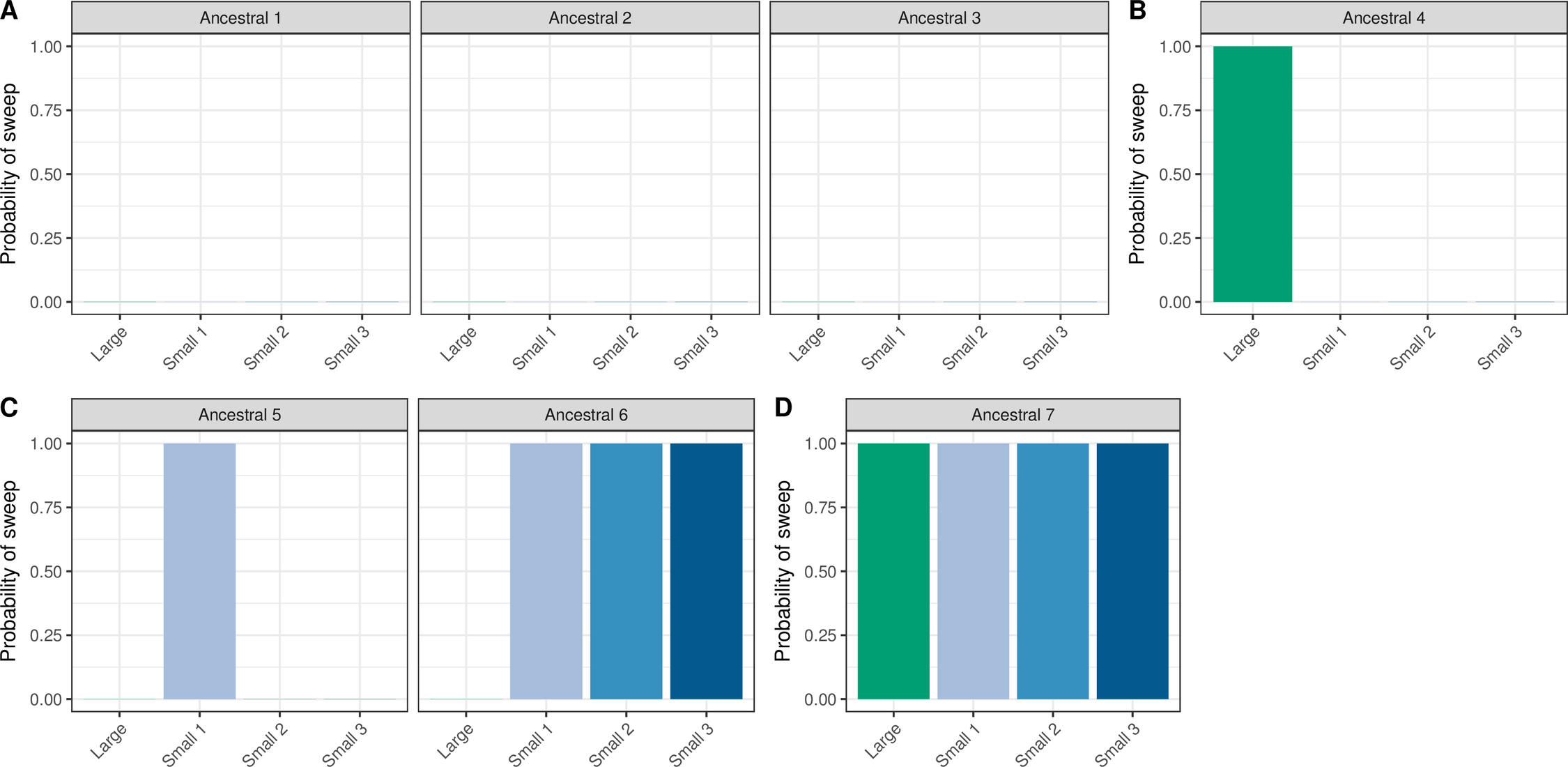

Supplement: S17 Fig — Estimated probability of a L28 sweep for each ancestral population used to seed the continued experimental evolution under each of the evolutionary regime (large or small population size); (A) for populations where L28 was lost before continued experimental evolution, (B) for the population where L28 swept in large populations, (C) for the populations where L28 swept in some of the small populations and (D) where L28 swept regardless of population size. (PNG) [file pgen.1007731.s018.png]
