# Supplementary material for: Slower environmental change hinders adaptation from standing genetic variation
Source: PLoS Genet. 2018 Nov 1;14(11):e1007731. doi: 10.1371/journal.pgen.1007731 (PMC6233921; doi:10.1371/journal.pgen.1007731)
Supplement: S2 Table — (PDF) [file pgen.1007731.s020.pdf]

				# inbred lines (and line ids)	
**Lineage**	**I & II**	**III & IV**	**V & X**	**GM150**	**GM350**
**G1**	H4A3	H3B4	H3C2		**30** (1,2,3,4,5,6,7,8,9,13,14, 15,17,18,19,21,24,26,27,30,32, 34,35,36,39,41,44,46,48,49)
**G2**	H3A4	H3B3	H4C2	**16** (4,5,10,14,16,19,20,22, 27,29,30,31,41,45,46,50)	
**G3**	H3A4	H3B3	H5C2	**11** (2,3,7,9,13,15,18, 21,25, 28,33)	
			H622C2		**1** (45)
**G4 (L11)**	H2A2	H2B2	H2C3	**1** (24)	**5** (11,12,23,25,50)
**G5**	H5A5	H13B7	H8C10	**4** (23,26,38,44)	
		H39B7	H8C7		**1** (37)
		H40B7		**1** (36)	
**G6**	H4A16	H3B4	H3C2		**4** (20,22,29,38)
**G7**	H6A21	H4B11	H16C4	**2** (37,42)	
	H64A15	H15B20	H48C20	**1** (40)	
**G8**	H7A7	H5B14	H51C11	**2** (8,39)	
			H500C11	**1** (6)	
**G9 (L28)**	H1A1	H1B1	H1C1	**1** (1)	**1** (28)
**G10**	H2A2	H2B6	H2C3		**2** (16,40)
**G11**	H6A12	H4B5	H14C4	**2** (11,47)	
**G12**	H6A12	H4B11	H10C4	**1** (35)	**1** (43)
**G13**	H27A11	H9B10	H9C8	**1** (32)	**1** (10)
**G14**	H2A55	H2B2	H2C3		**1** (47)
	H2A120				**1** (42)
**G15**	H3A101	H3B3	H4C2	**1** (12)	
	H165A4			**1** (49)	
**G16**	H2A2	H7B2	H2C3	**1** (48)	
**G17**	H6A12	H4B11	H14C4	**1** (17)	
**G18**	H5A5	H17B7	H13C7	**1** (43)	
**G19**	H6A21	H4B5	H16C4		**1** (31)
**G20**	H2A55	H2B6	H2C3		**1** (33)
**G21**	H5A5	H386B7	H13C7	**1** (34)	

Not observed in populations

Minor RWH
